# Author Correction: LINE-1 RNA triggers matrix formation in bone cells via a PKR-mediated inflammatory response

**DOI:** 10.1038/s44318-024-00243-w

**Published:** 2024-09-20

**Authors:** Arianna Mangiavacchi, Gabriele Morelli, Sjur Reppe, Alfonso Saera-Vila, Peng Liu, Benjamin Eggerschwiler, Huoming Zhang, Dalila Bensaddek, Elisa A Casanova, Carolina Medina Gomez, Vid Prijatelj, Francesco Della Valle, Nazerke Atinbayeva, Juan Carlos Izpisua Belmonte, Fernando Rivadeneira, Paolo Cinelli, Kaare Morten Gautvik, Valerio Orlando

**Affiliations:** 1 King Abdullah University of Science and Technology (KAUST), Biological Environmental Science and Engineering Division, Thuwal, 23500-6900 Kingdom of Saudi Arabia; 2 Oslo University Hospital, Department of Medical Biochemistry, Oslo, Norway; 3 Lovisenberg Diaconal Hospital, Unger-Vetlesen Institute, Oslo, Norway; 4 Oslo University Hospital, Department of Plastic and Reconstructive Surgery, Oslo, Norway; 5 Sequentia Biotech, Carrer Comte D’Urgell 240, Barcelona, 08036 Spain; 6 Department of Trauma, University Hospital Zurich, Sternwartstrasse 14, 8091 Zurich, Switzerland; 7 Life Science Zurich Graduate School, University of Zurich, Winterthurerstrasse 190, 8057 Zurich, Switzerland; 8 Core Labs, King Abdullah University of Science and Technology (KAUST), Thuwal, 23500-6900 Kingdom of Saudi Arabia; 9 Department of Internal Medicine, Erasmus Medical Centre, Rotterdam, the Netherlands; 10 Altos Labs, San Diego, CA USA; 11 Center for Applied Biotechnology and Molecular Medicine, University of Zurich, Winterthurerstrasse 190, 8057 Zurich, Switzerland

## Abstract

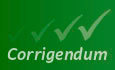

**Correction to:** *The EMBO Journal* (2024) 43:3587–3603. 10.1038/s44318-024-00143-z | Published online 1 July 2024

**Two author affiliations are corrected**.

**The acknowledgement section is corrected**.

**One additional reference is added**.

The author affiliations for authors, Elisa A Casanova and Paolo Cinelli are corrected.

The correct affiliation for Elisa A Casanova is: ^6^Department of Trauma, University Hospital Zurich, Sternwartstrasse 14, 8091, Zurich, Switzerland

The correct affiliations for Paolo Cinelli are:

^6^Department of Trauma, University Hospital Zurich, Sternwartstrasse 14, 8091, Zurich, Switzerland

&

^11^Center for Applied Biotechnology and Molecular Medicine, University of Zurich, Winterthurerstrasse 190, 8057 Zurich, Switzerland.

The Acknowledgement section is corrected from:

Funding: We are grateful for support from: KAUST BAS/1/1037-01-01, the European Union project OSTEOGENE (no. FP6-502491); ZonMw VIDI 016.136.367 grant, for funding the creation of the RNA-Seq dataset of hip primary bone and Oslo University Hospital, Ullevål, project #29750104.

To: (Changes in bold)

Funding: We are grateful for support from: KAUST BAS/1/1037-01-01, **Hevolution Foundation**, the European Union project OSTEOGENE (no. FP6-502491); ZonMw VIDI 016.136.367 grant, for funding the creation of the RNA-Seq dataset of hip primary bone and Oslo University Hospital, Ullevål, project #29750104.

One additional reference is added to the manuscript.

**Angileri K, Nornubari A Bagia, Feschotte C (2022) Transposons control as a checkpoint for tissue regeneration. Development 149(22):dev191957**

The reference is added to the beginning of the discussion section

(Insertion is in bold)

Discussion

The concerted co-option of TEs markedly changed whole regulatory networks and integrated new functions into the eukaryote genome. (Cosby et al, 2019) (Mangiavacchi et al, 2021) (Carelli et al, 2022). However, while the role of TEs as evolutionary drivers is today well-established, their contribution to cell physiology, particularly in somatic cells, remains to be elucidated. Indeed, aside from their role in the nucleus, (Della Valle et al, 2022) (Jachowicz et al, 2017) (Lu et al, 2021) (Percharde et al, 2018) TEs represent a major source of endogenous dsRNA whose **essential role in inflammatory and innate immune responses involved in various physiological processes** has increasingly gained attention (Sadeq et al, 2021) (Chen and Hur, 2022) (**Angileri et al, 2022**).

