# Supplementary material for: LINE-1 RNA triggers matrix formation in bone cells via a PKR-mediated inflammatory response
Source: EMBO J. 2024 Jul 1;43(17):3587–603. doi: 10.1038/s44318-024-00143-z (PMC11377738; doi:10.1038/s44318-024-00143-z)
Supplement: Supplementary file 1 — Appendix [file 44318_2024_143_MOESM1_ESM.pdf]

LINE-1 RNA triggers matrix formation in bone cells via a PKR-mediated inflammatory response

Arianna Mangiavacchi*, Gabriele Morelli, Sjur Reppe, Alfonso Saera-Vila, Peng Liu, Benjamin Eggerschwiler, Huoming Zhang, Dalila Bensaddek, Elisa A. Casanova, Carolina Medina Gomez, Vid Prijatelj, Francesco Della Valle, Nazerke Atinbayeva, uan Carlos Izpisua Belmonte, Fernando Rivadeneira, Paolo Cinelli, Kaare Morten Gautvik, Valerio Orlando*

*corresponding authors

Table of content

Appendix Figure S1: Timeline expression of late differentiation markers in L1 treated osteoblasts……3

Appendix Figure S2: ALPL and ENPP1 activity in L1 treated osteoblasts…………………………………..4

Appendix Figure S3: Number of DEG between L1 and RFP (RNAseq data)………………………………..4

Appendix Figure S4: Colocalization between exogenous L1 RNA and DNA:RNA hybrid IF signal…5

Appendix Figure S5: ORF1p expression in L1 transfected cells…………………………………………………5

Appendix Figure S6: Effect of PKR and cGAS knockdown on L1 RNA-induced mineralization……..6

Appendix Table S1: Cohort of femoral bone biopsies……………………………………………………………..7

Appendix Table S2: Cohort of iliac bone biopsies……………………………………………………………………8

Appendix Table S3: qPCR primers used in this study……………………………………………………………9

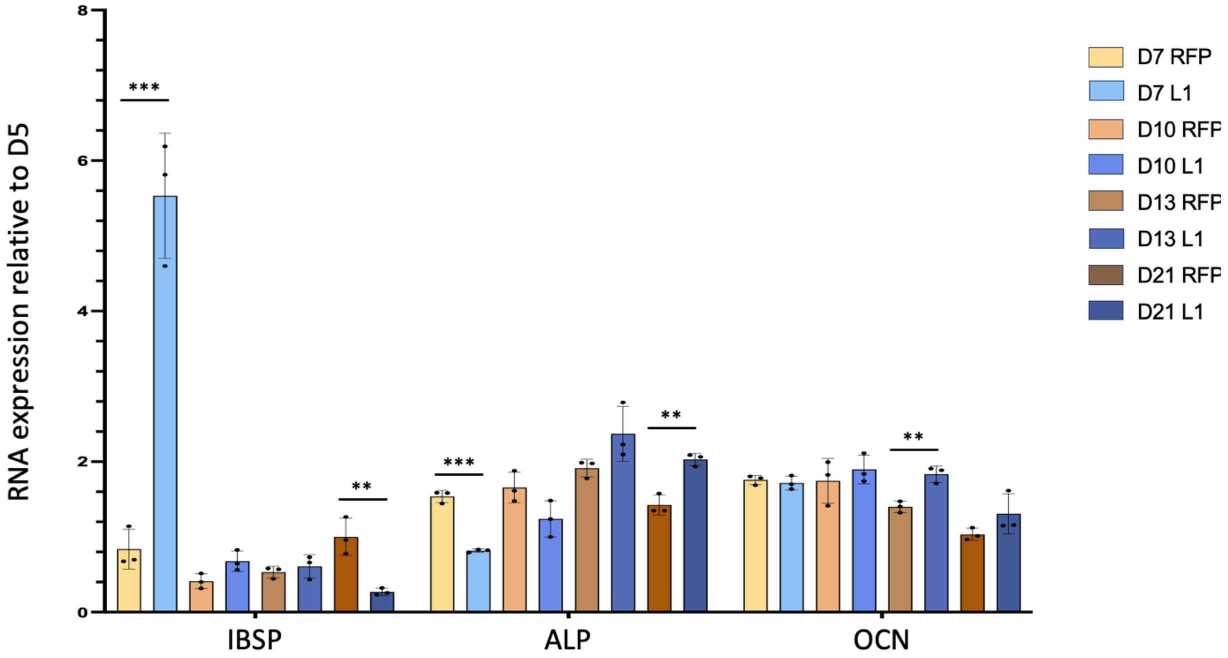

Appendix Figure S1: Timeline expression of late differentiation markers in L1 treated osteoblasts

qRT-PCR of osteogenic genes in RFP and L1 transfected osteoblasts at different time points of osteogenic differentiation. Expression level is normalized on day 5 (not transfected osteoblasts). The graph is shown as mean ± s.d. of n = 3 independent experiments. *P < 0.05; **P < 0.005, ***P < 0.0005 in Student's *t* test.

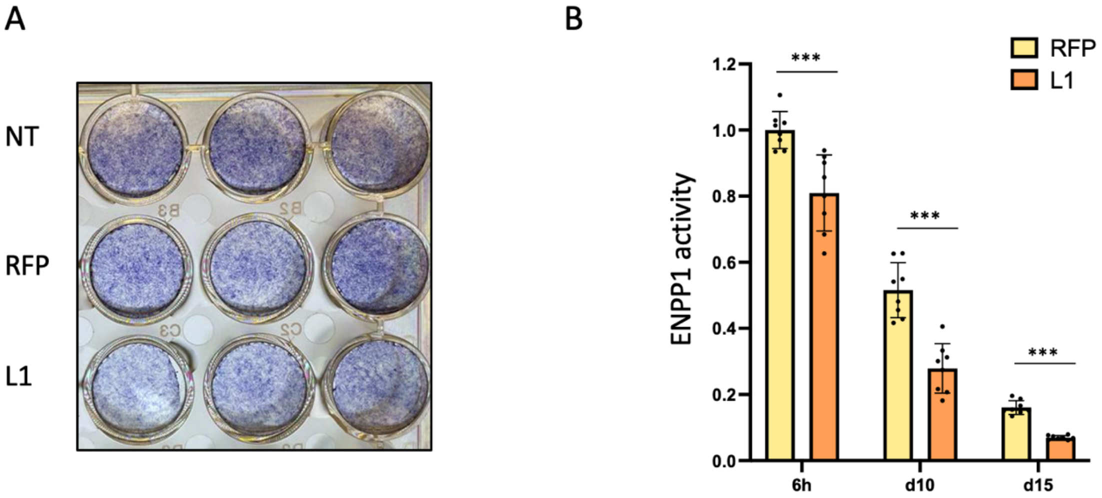

Appendix Figure S2: ALPL and ENPP1 activity in L1 treated osteoblasts

A) Colorimetric ALPL activity assay of osteoblasts 5 days post-L1 transfection. B) ENPP1 activity of osteoblasts 6h, 5 days (d10) and 10 days (d15) after RFP and L1 transfection. The graph is shown as mean ± s.d. of n = 3 independent experiments. ***P < 0.0005 in Student's t test.

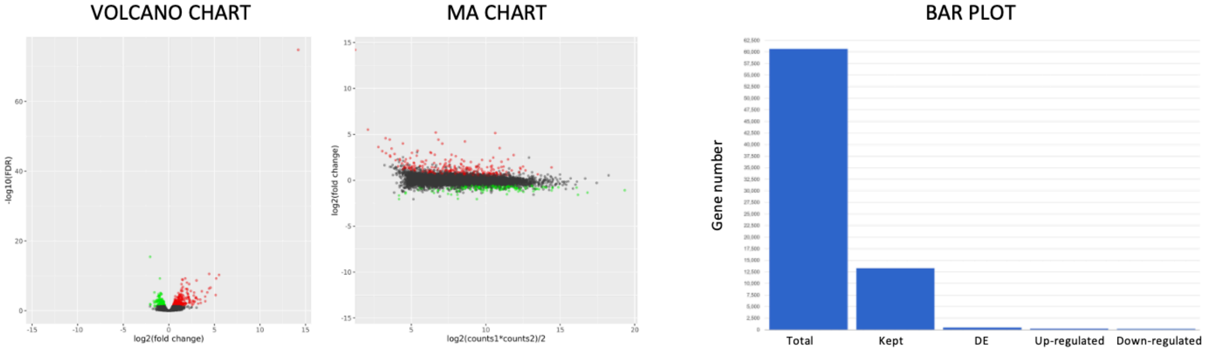

Appendix Figure S3: Number of DEG between L1 and RFP (RNAseq data)

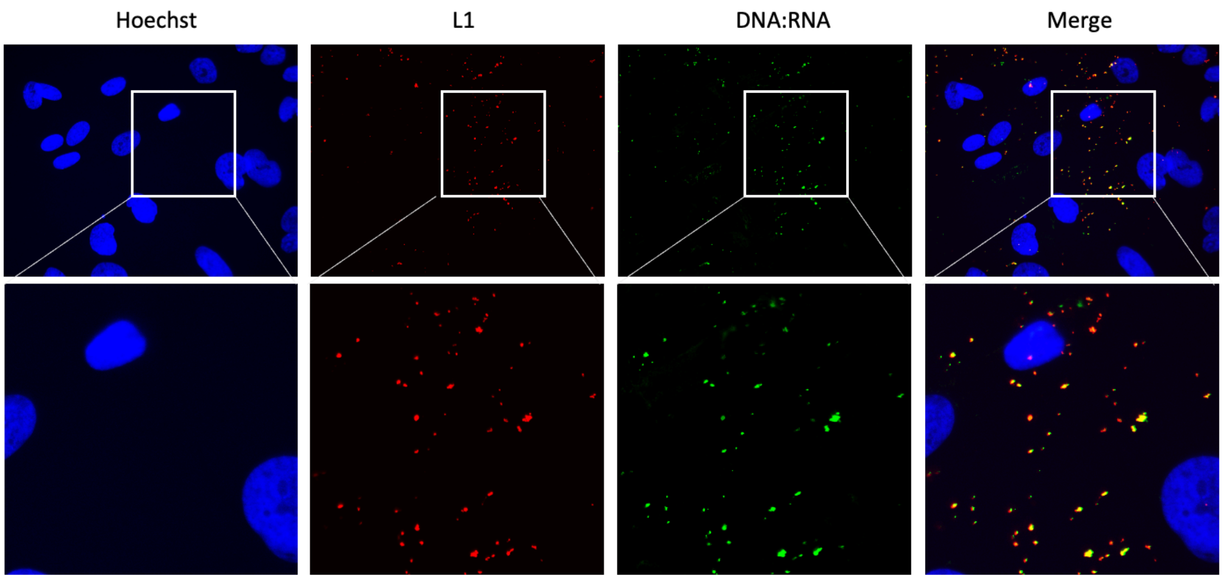

Appendix Figure S4: Colocalization between exogenous L1 RNA and DNA:RNA hybrid IF signal
Immunofluorescence of DNA:RNA hybrids (green) in osteoblasts 24h after the transfection of cy5-coniugated L1 RNA (red).

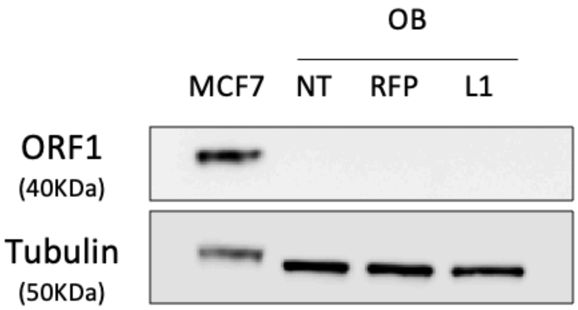

Appendix Figure S5: ORF1p expression in L1 transfected cells
Western Blot of ORF1 in untransfected (NT), RFP- and L1-transfected differentiating osteoblasts (OB). Tubulin is used as endogenous calibrator. Human breast cancer cell line (MCF7) extract is used as positive control.

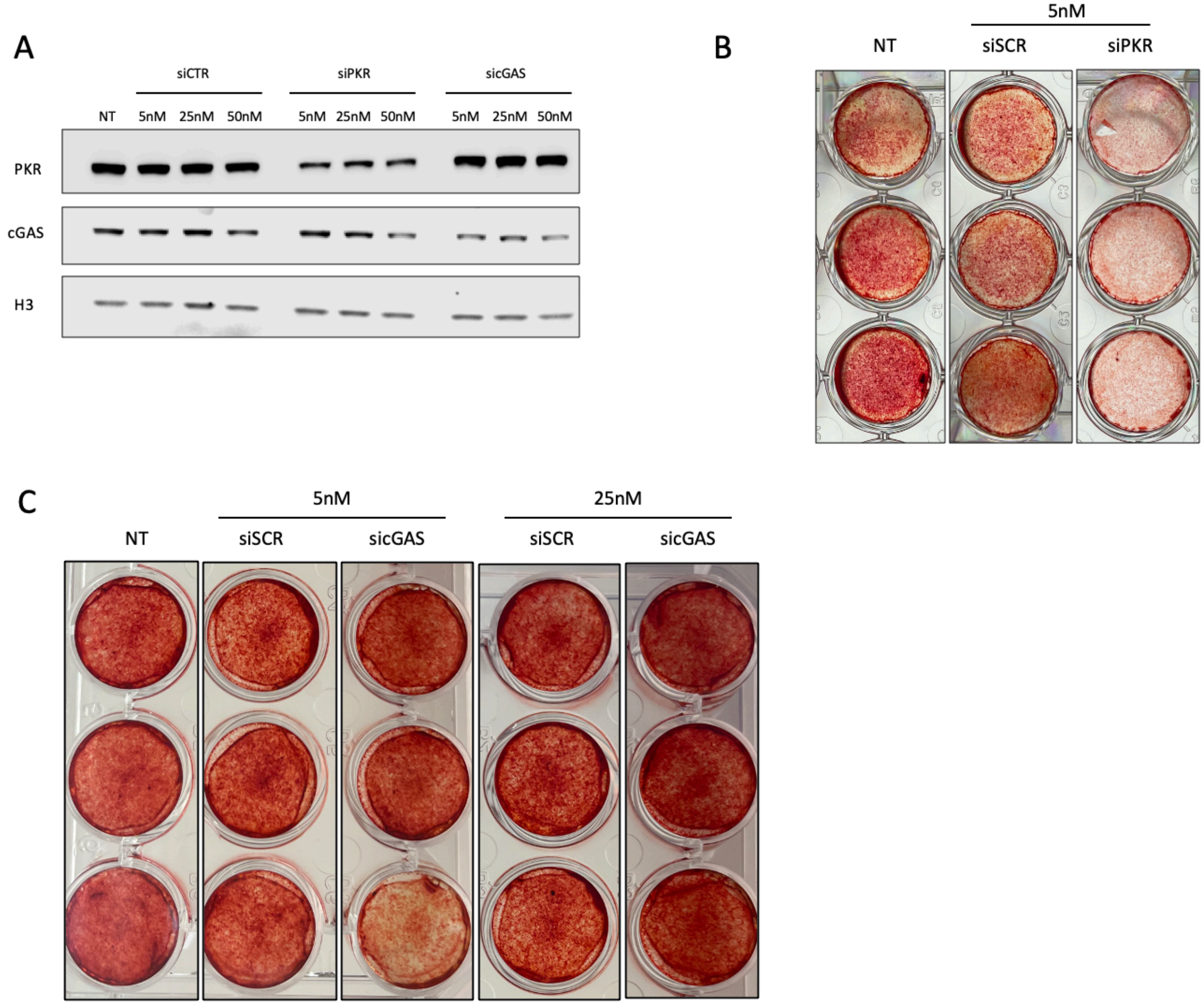

Appendix Figure S6: Effect of PKR and cGAS knockdown on L1 RNA-induced mineralization

A) Western Blot of PKR and cGAS in L1-expressing osteobalsts transfected with three different doses of siRNA. Histone H3 is used as endogenous calibrator. B) Alizarin red staining on L1-expressing osteoblasts transfected with a 5nM pool of PKR targeting siRNAs. C) Alizarin red staining on L1-expressing osteoblasts transfected with a 5nM and 25nM pool of cGAS targeting siRNAs.

Sample	Age	BMI	Sex	FN_TotTScore	group
**LDS119A**	62,3	32,1144642839595	F	0,85	normal
**LDS121A**	75,1	29,8287149306521	F	-0,50	normal
**LDS122A**	81,6	29,6296296296296	F	0,40	normal
**LDS124A**	52,4	28,7295259628216	F	0,20	normal
**DH205A**	76,4	26,2487601666336	M	-1,20	osteopenic
**DH317A**	72,2	24,0187668499002	F	-2,70	osteoporotic
**LDS6A**	74,0	20,6174477392520	M	-0,65	normal
**LDS105A**	73,7	26,3979773944081	F	-2,60	osteoporotic
**DH318A**	67,8	25,5102040816327	F	-2,60	osteoporotic
**LDS110A**	66,4	30,3757724007630	F	-0,05	normal
**LDS3A**	75,0	35,1328623076425	M	-0,05	normal
**LDS112A**	78,5	25,3391251333638	F	-1,40	osteopenic
**DH301A**	77,7	27,1044021415824	F	-1,90	osteopenic
**LDS111A**	66,7	28,1359044995409	F	-0,50	normal
**LDS106A**	53,1	21,1072664359862	F	-1,10	osteopenic
**LDS8A**	68,4	29,1815509347440	M	1,00	normal
**LDS113A**	73,6	20,7152052357976	F	-2,30	osteopenic
**LDS17A**	74,2	24,3227508283679	M	0,45	normal
**LDS114A**	77,1	28,7403331350387	F	-1,65	osteopenic
**LDS4A**	74,9	23,9463601532567	M	-0,60	normal
**LDS107A**	77,5	30,9688581314879	F	0,75	normal
**LDS103A**	73,2	22,9118356260567	F	-0,85	normal
**DH304A**	71,4	25,0399187109885	F	-3,00	osteoporotic
**LDS9A**	66,9	25,6360518617950	M	1,25	normal
**LDS115A**	63,3	34,7222222222222	F	0,50	normal
**DH309A**	77,3	25,1038781163435	F	-1,20	osteopenic
**LDS118A**	69,4	23,1472551213302	F	-3,10	osteoporotic
**DH310A**	80,5	23,3976124885216	F	-1,80	osteopenic
**DH203A**	49,9	28,2072728109821	M	-2,60	osteoporotic
**LDS128A**	89,9	27,0538027739674	F	-1,45	osteopenic
**DH315A**	63,9	18,2278307855409	F	-2,20	osteopenic
**LDS116A**	57,6	34,0733811660978	F	0,60	normal
**DH303A**	80,0	21,8517157272211	F	-2,30	osteopenic
**LDS15A**	63,6	22,0931700542861	M	-2,60	osteoporotic
**LDS1A**	59,0	27,2853485703280	M	0,10	normal
**DH306A**	58,6	29,3788860043791	F	-2,10	osteopenic
**DH311A**	73,8	16,6888957286003	F	-2,80	osteoporotic
**DH308A**	69,9	21,8846068166630	F	-0,80	normal
**LDS123A**	77,4	28,4608265581307	F	-2,50	osteoporotic
**LDS16A**	88,4	22,4711928245396	M	0,10	normal
**LDS14A**	75,9	23,4425078599554	M	-0,80	normal
**LDS125A**	78,3	24,3764172335601	F	-0,90	normal
**DH313A**	72,1	25,9959123668830	F	-0,30	normal
**LDS2A**	59,1	27,8547633044988	M	0,90	normal
**DH314A**	74,6	24,3646292795631	F	-0,80	normal
**LDS19A**	74,8	25,2831641734658	M	-0,30	normal
**LDS109A**	44,8	26,1793740810754	F	0,90	normal
**LDS13A**	73,6	27,2618444341625	M	0,25	normal

Appendix Table S1: Cohort of femoral bone biopsies

Sample	Age	BMI	Sex	FN_TotTScore	group
**i99**	80,7	27,2562498	F	-1,10	osteopenic
**i100**	76,9	19,2708497	F	-3,70	osteoporotic
**i86**	52,9	21,3040032	F	1,70	normal
**i93**	62,5	22,7286571	F	-2,80	osteoporotic
**i98**	65,1	22,9118356	F	1,20	normal
**i97**	79,0	22,2331151	F	0,60	normal
**i66**	49,7	24,4039074	F	-0,55	normal
**i80**	72,2	30,7480666	F	-0,90	normal
**i2**	82,8	22,2107438	F	-4,10	osteoporotic
**i24**	51,8	28,7334594	F	-0,10	normal
**i40**	86,1	20,0350293	F	-3,95	osteoporotic
**i67**	60,5	21,6622416	F	-0,05	normal
**i81**	60,2	23,5000000	F	0,30	normal
**i6**	57,7	16,1941777	F	-3,10	osteoporotic
**i59**	56,0	24,5000000	F	-0,20	normal
**i25**	64,4	22,5069252	F	1,90	normal
**i12**	66,8	21,5598502	F	-2,90	osteoporotic
**i38**	67,5	23,3217993	F	-1,95	osteopenic
**i27**	83,0	20,8209399	F	-3,75	osteoporotic
**i42**	70,4	33,1571023	F	0,50	normal
**i65**	57,1	29,2011019	F	0,50	normal
**i69**	66,2	27,5802003	F	0,60	normal
**i82**	71,1	26,2030738	F	0,20	normal
**i10**	56,9	23,4935752	F	-2,05	osteopenic
**i68**	54,9	27,4285714	F	-0,10	normal
**i43**	69,4	23,2520710	F	-1,60	osteopenic
**i28**	53,5	20,1408042	F	-0,60	normal
**i44**	67,3	22,8643755	F	0,60	normal
**i57**	74,8	27,0144141	F	-2,20	osteopenic
**i71**	52,6	27,7318641	F	-0,70	normal
**i70**	71,9	29,0174472	F	-2,70	osteoporotic
**i87**	55,5	25,8199104	F	-0,10	normal
**i19**	51,6	19,0617914	F	-2,30	osteopenic
**i84**	62,5	36,3372093	F	0,95	normal
**i78**	59,5	23,0915415	F	-1,60	osteopenic
**i20**	57,2	23,4606914	F	-2,90	osteoporotic
**i29**	84,1	24,0234375	F	-2,60	osteoporotic
**i45**	64,3	26,3043392	F	-1,75	osteopenic
**i72**	53,7	22,8000000	F	-1,40	osteopenic
**i90**	65,1	24,9035813	F	-0,80	normal
**i26**	60,8	28,3930211	F	1,55	normal
**i89**	80,7	26,0633551	F	-0,80	normal
**i15**	77,9	26,0789715	F	-2,40	osteopenic
**i34**	54,8	23,3843537	F	1,10	normal
**i32**	58,4	19,7742023	F	-1,30	osteopenic
**i56**	59,7	21,3227890	F	0,10	normal
**i46**	57,2	22,0580691	F	0,30	normal
**i60**	57,6	22,4913495	F	-1,65	osteopenic
**i74**	68,8	26,0950917	F	1,70	normal
**i91**	54,3	24,0776773	F	2,00	normal
**i30**	61,5	24,4739330	F	-2,60	osteoporotic
**i16**	73,9	21,8299522	F	-2,20	osteopenic
**i75**	67,5	24,7474352	F	-2,20	osteopenic
**i92**	76,7	20,9300000	F	-2,70	osteoporotic
**i33**	54,6	20,7736572	F	-0,90	normal
**i35**	59,0	25,8000000	F	0,20	normal
**i49**	56,6	28,0622837	F	1,75	normal
**i62**	60,0	20,6350387	F	-2,10	osteopenic
**i79**	65,9	22,4673868	F	-1,30	osteopenic
**i22**	81,3	27,2215779	F	-2,75	osteoporotic
**i36**	54,9	26,8122187	F	-1,40	osteopenic
**i76**	77,9	19,1326531	F	-1,70	osteopenic
**i94**	59,6	23,7166718	F	-1,50	osteopenic
**i41**	68,8	21,5138585	F	-1,40	osteopenic
**i50**	57,7	22,8373702	F	0,30	normal
**i95**	70,6	20,6250000	F	0,80	normal
**i37**	68,3	19,2096838	F	-3,55	osteoporotic
**i64**	58,1	30,0621020	F	0,10	normal
**i55**	83,3	25,8853816	F	-2,95	osteoporotic
**i47**	53,7	18,8277447	F	-2,70	osteoporotic
**i21**	70,6	18,8581315	F	-3,20	osteoporotic

Appendix Table S2: Cohort of iliac bone biopsies

PRIMER NAME	FW SEQUENCE	REV SEQUENCE
L1 5'UTR-ORF1	GAATGATTTTGACGAGCTGAGAGAA	GTCCTCCCGTAGCTCAGAGTAATT
RPL13A	GAAAGCCAAGATCCACTACC	TGGGTCTTGAGGACCTCTGT
CXCL3	CGCCCAAACCGAAGTCATAG	GCTCCCCTTGTTCAGTATCTTTT
CXCL8	GAGAGTGATTGAGAGTGGACCAC	CACAACCCTCTGCACCCAGTTT
CCL20	TGCTGTACCAAGAGTTTGCTC	CGCACACAGACAACTTTTTCTTT
CXCL6	AGAGCTGCGTTGCACTTGTT	GCAGTTTACCAATCGTTTTGGGG
CCL3	AGTTCTCTGCATCACTTGCTG	CGGCTTCGCTTGGTTAGGAA
CCL2	CAGCCAGATGCAATCAATGCC	TGGAATCCTGAACCCACTTCT
CXCL5	AGCTGCGTTGCGTTTGTTTAC	TGGCGAACACTTGCAGATTAC
CXCL10	GTGGCATTCAAGGAGTACCTC	TGATGGCCTTCGATTCTGGATT
PTGS2	TAAGTGCGATTGTACCCGGAC	TTTGTAGCCATAGTCAGCATTGT
TLR2	ATCCTCCAATCAGGCTTCTCT	GGACAGGTCAAGGCTTTTTACA
CXCL2	GGCAGAAAGCTTGTCTCAACCC	CTCCTTCAGGAACAGCCACCAA
CXCL1	AGCTTGCCTCAATCCTGCATCC	TCCTTCAGGAACAGCCACCAGT
BMP2	ACTACCAGAAACGAGTGGGAA	GCATCTGTTCTGGAAAACCT
RUNX2	TCAACGATCTGAGATTTGTGGG	GGGGAGGATTTGTGAAGACGG
MSX2	CACCCTGAGGAAACACAAGAC	TGCACGCTCTGAATGGAG
ALPL	ACTGGTACTCAGACAACGAGAT	ACGTCAATGTCCCTGATGTTATG
OCN	GGCGCTACCTGTATCAATGG	GTGGTCAGCCAACTCGTCA
IBSP	CACTGGAGCCAATGCAGAAGA	TGGTGGGGTTGTAGGTTCAAA

Appendix Table S3: qPCR primers used in this study
