# Supplementary material for: LINE-1 RNA triggers matrix formation in bone cells via a PKR-mediated inflammatory response
Source: EMBO J. 2024 Jul 1;43(17):3587–603. doi: 10.1038/s44318-024-00143-z (PMC11377738; doi:10.1038/s44318-024-00143-z)
Supplement: Supplementary file 4 — Source data Fig. 3 [file 44318_2024_143_MOESM4_ESM.zip › Figure 3/3E/3E README.docx]

These images were taken with the camera of an iPhone through the ocular of the microscope.
