# Supplementary figures and images for: LINE-1 RNA triggers matrix formation in bone cells via a PKR-mediated inflammatory response
Source: EMBO J. 2024 Jul 1;43(17):3587–603. doi: 10.1038/s44318-024-00143-z (PMC11377738; doi:10.1038/s44318-024-00143-z)

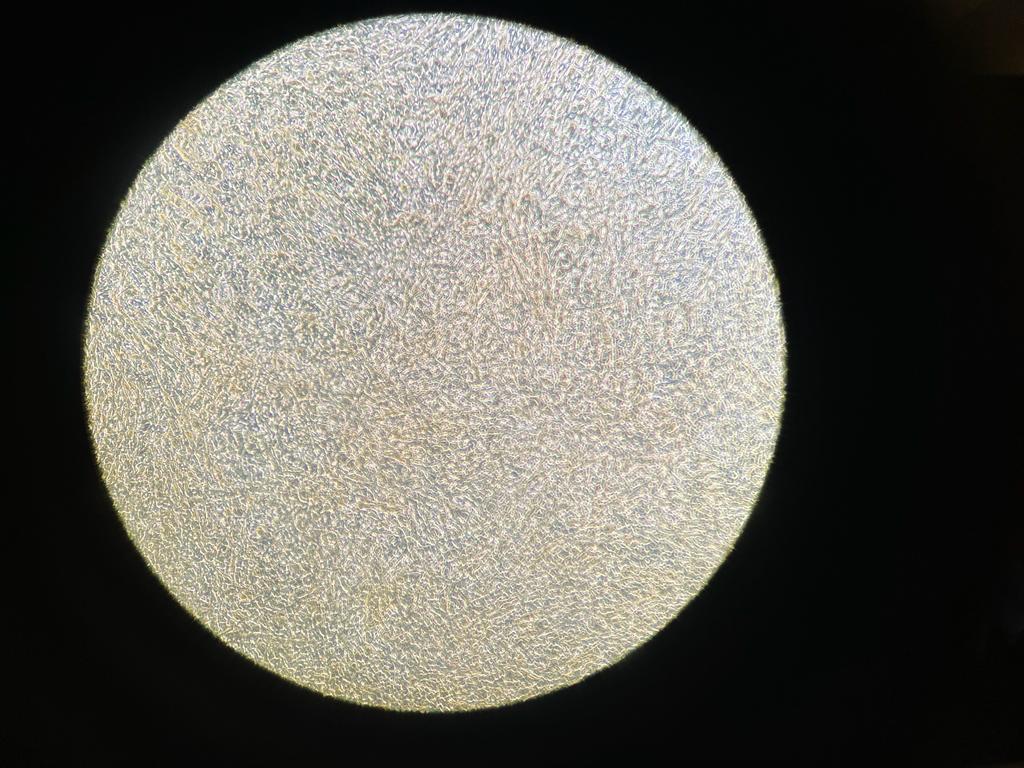

Supplement: Supplementary file 4 — Source data Fig. 3 [file 44318_2024_143_MOESM4_ESM.zip › Figure 3/3E/RFP 10,6 .jpg]

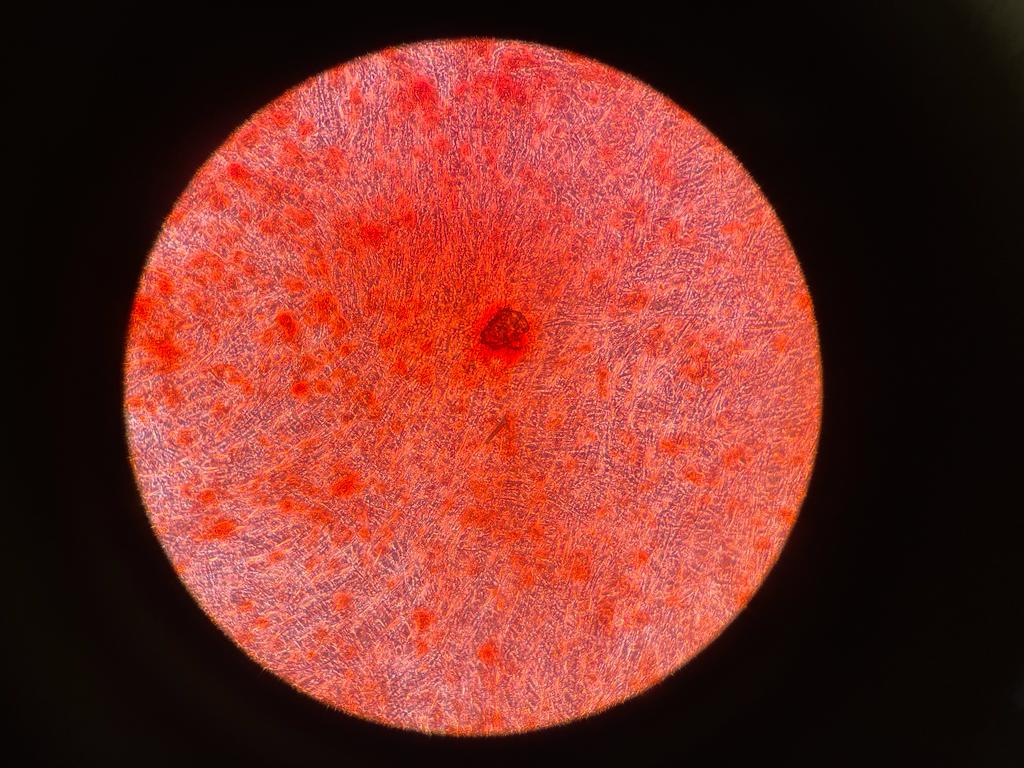

Supplement: Supplementary file 4 — Source data Fig. 3 [file 44318_2024_143_MOESM4_ESM.zip › Figure 3/3E/L1 70.jpg]

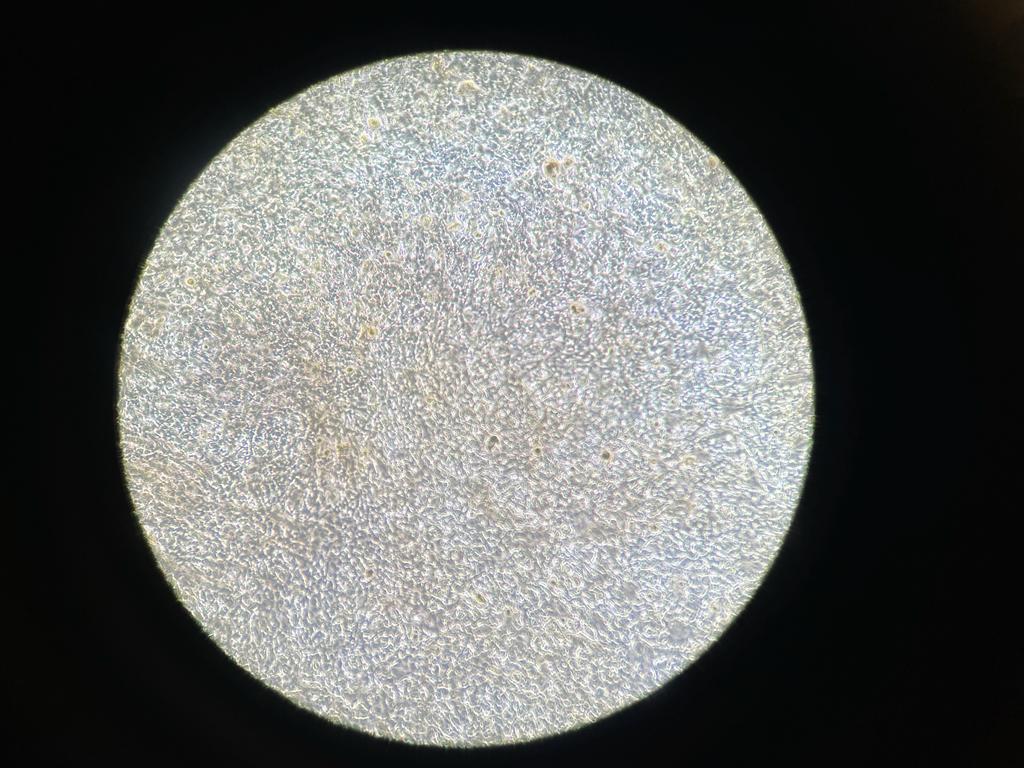

Supplement: Supplementary file 4 — Source data Fig. 3 [file 44318_2024_143_MOESM4_ESM.zip › Figure 3/3E/RFP 116.jpg]

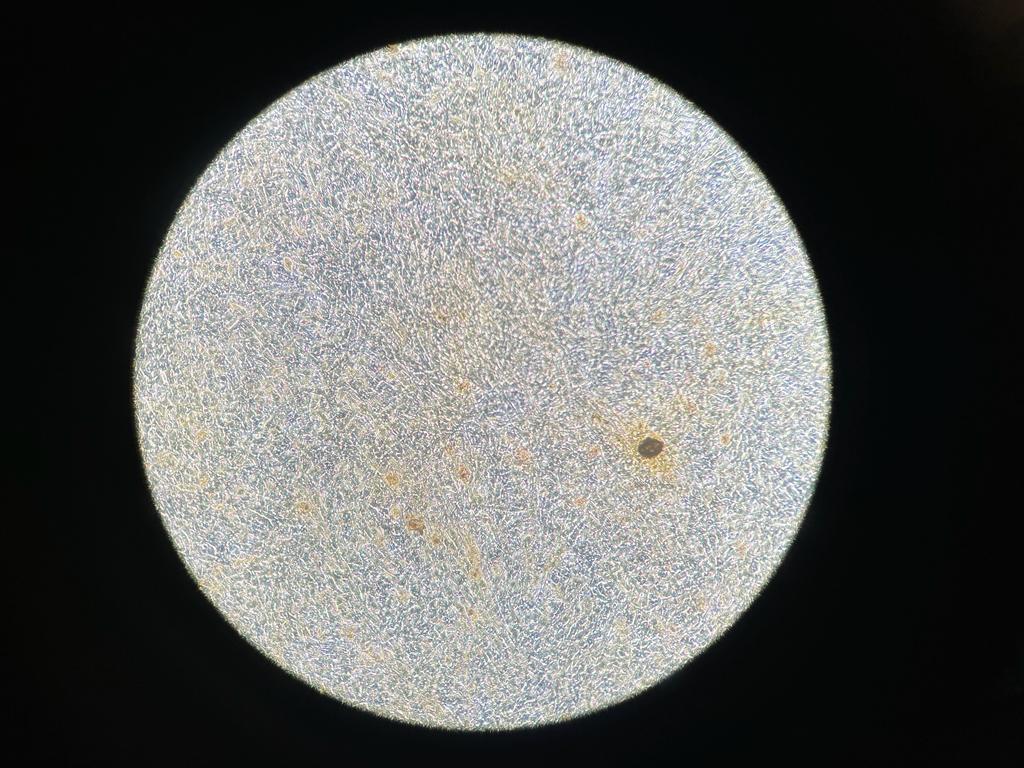

Supplement: Supplementary file 4 — Source data Fig. 3 [file 44318_2024_143_MOESM4_ESM.zip › Figure 3/3E/RFP 70.jpg]

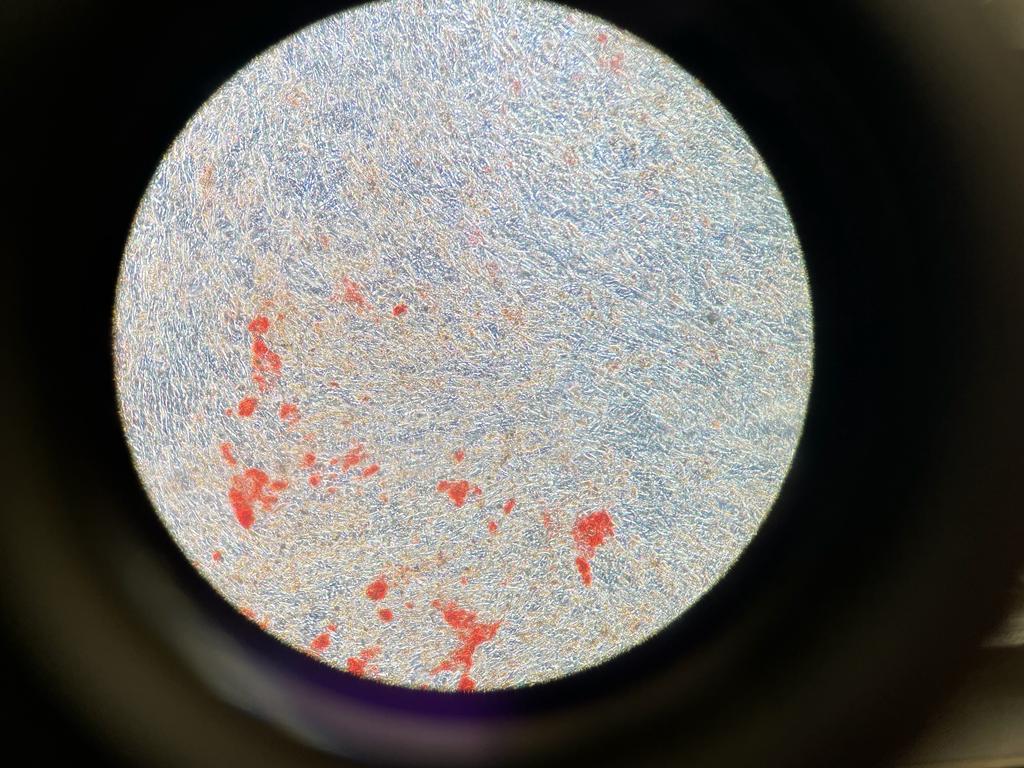

Supplement: Supplementary file 4 — Source data Fig. 3 [file 44318_2024_143_MOESM4_ESM.zip › Figure 3/3E/RFP770.jpg]

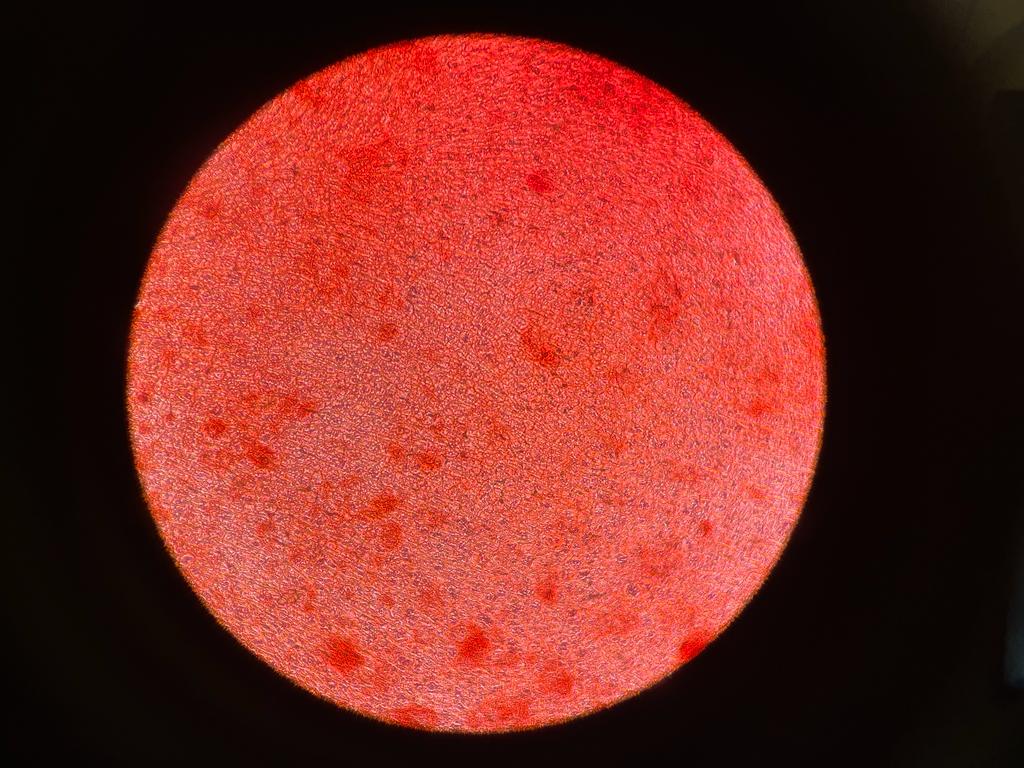

Supplement: Supplementary file 4 — Source data Fig. 3 [file 44318_2024_143_MOESM4_ESM.zip › Figure 3/3E/L1 32.jpg]

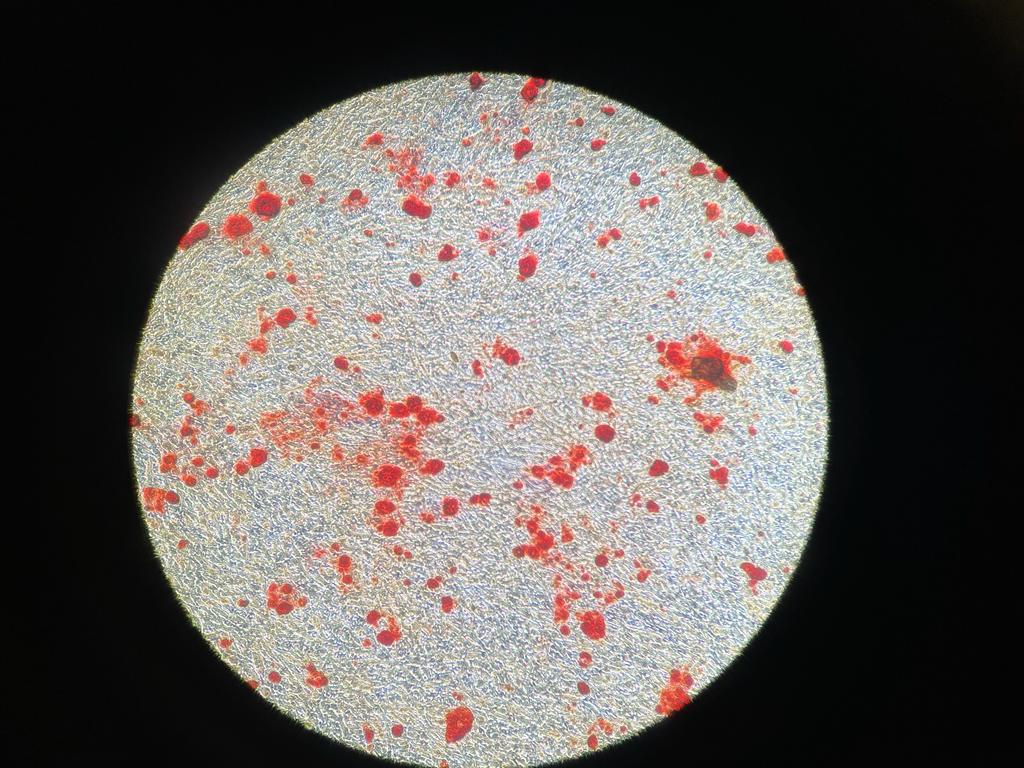

Supplement: Supplementary file 4 — Source data Fig. 3 [file 44318_2024_143_MOESM4_ESM.zip › Figure 3/3E/L1 3,5.jpg]

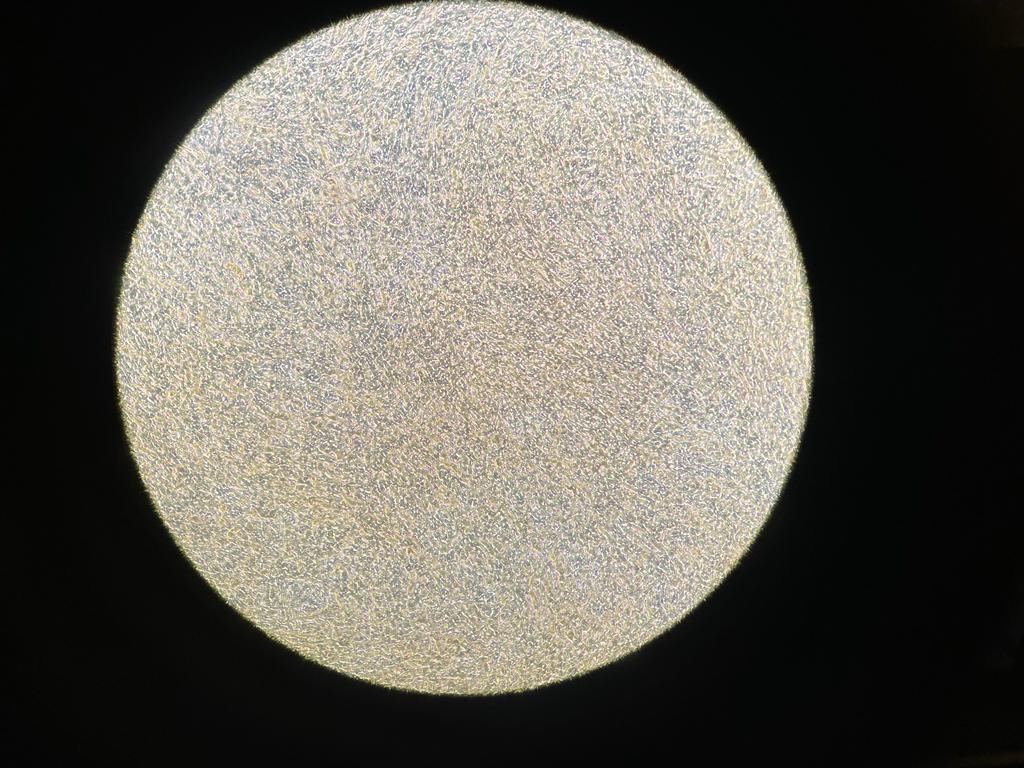

Supplement: Supplementary file 4 — Source data Fig. 3 [file 44318_2024_143_MOESM4_ESM.zip › Figure 3/3E/RFP 3,5.jpg]

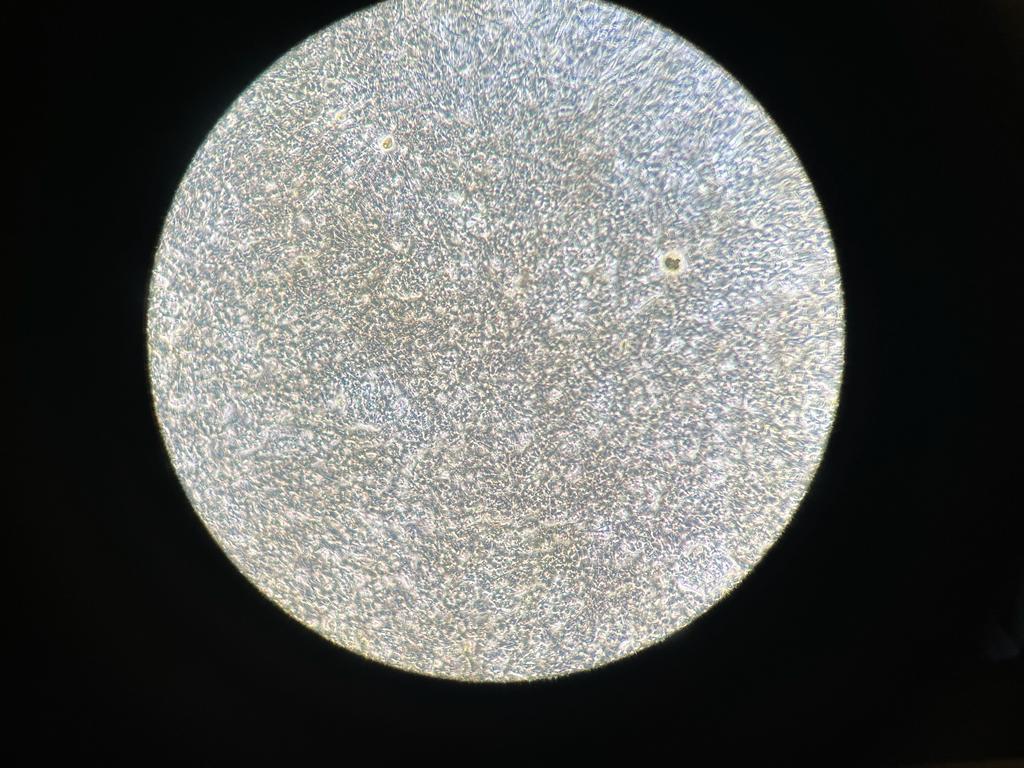

Supplement: Supplementary file 4 — Source data Fig. 3 [file 44318_2024_143_MOESM4_ESM.zip › Figure 3/3E/RFP 32.jpg]

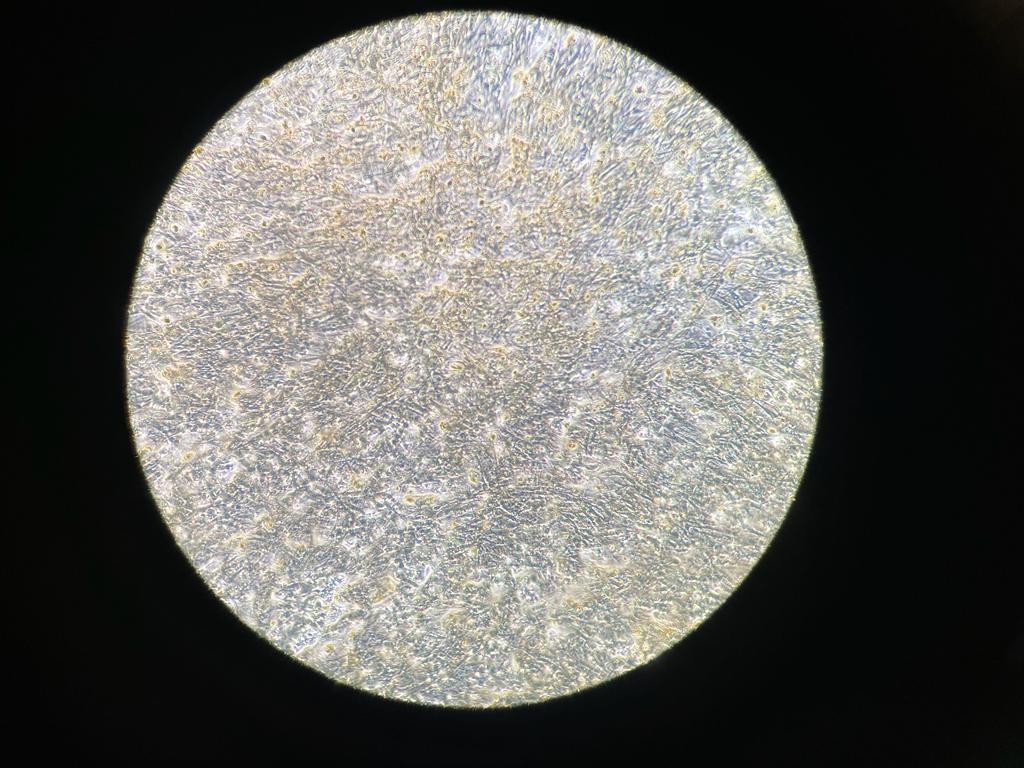

Supplement: Supplementary file 4 — Source data Fig. 3 [file 44318_2024_143_MOESM4_ESM.zip › Figure 3/3E/RFP 350.jpg]

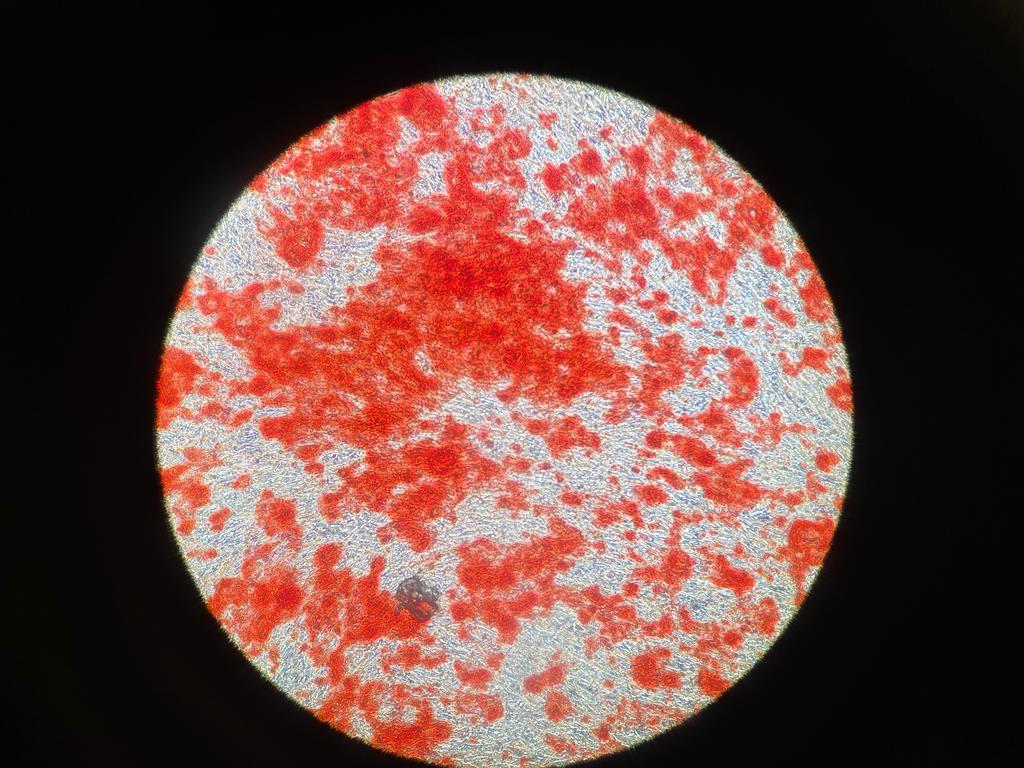

Supplement: Supplementary file 4 — Source data Fig. 3 [file 44318_2024_143_MOESM4_ESM.zip › Figure 3/3E/L1 10,6.jpg]

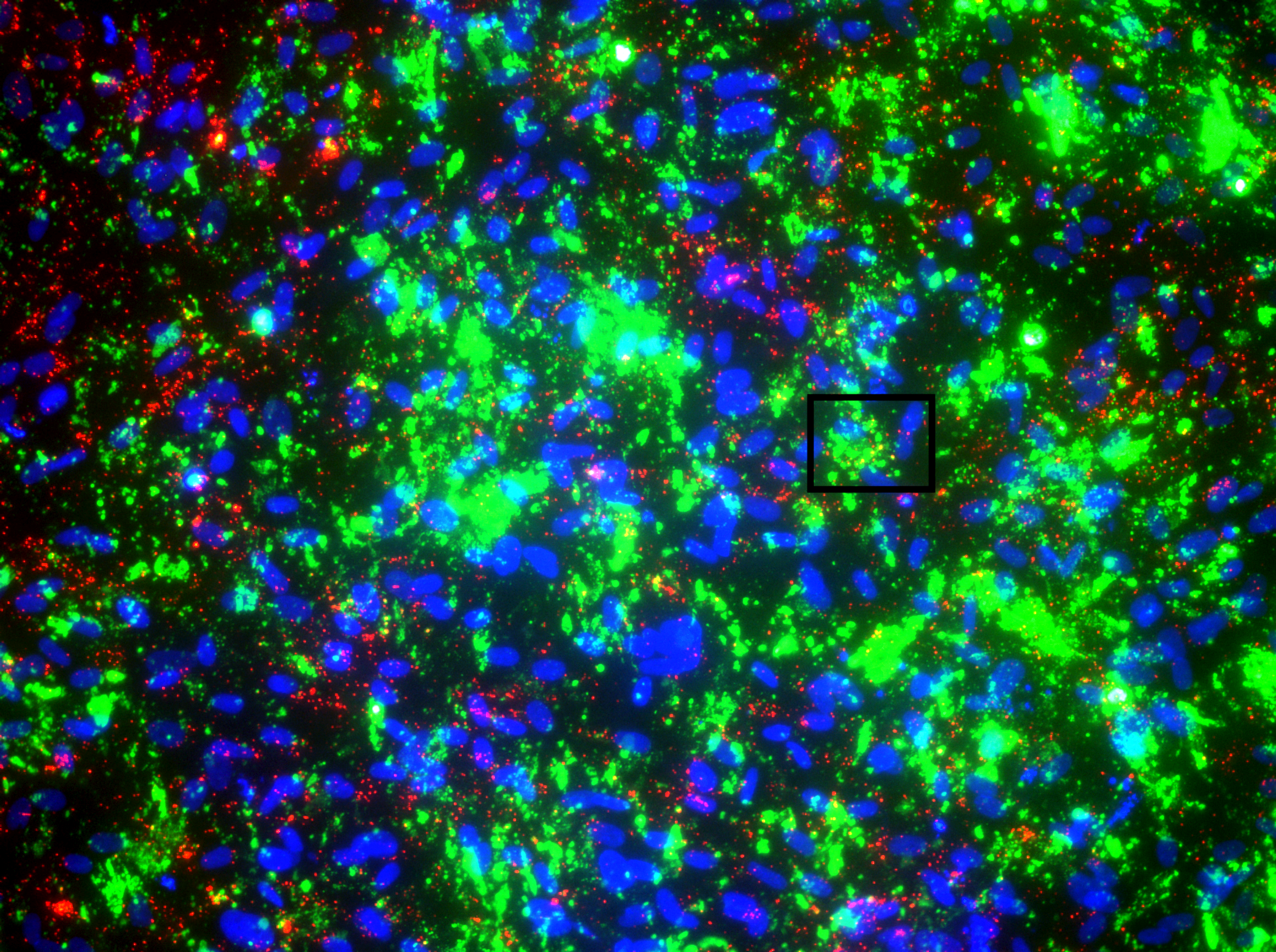

Supplement: Supplementary file 4 — Source data Fig. 3 [file 44318_2024_143_MOESM4_ESM.zip › Figure 3/3C/3C micr image.TIF]

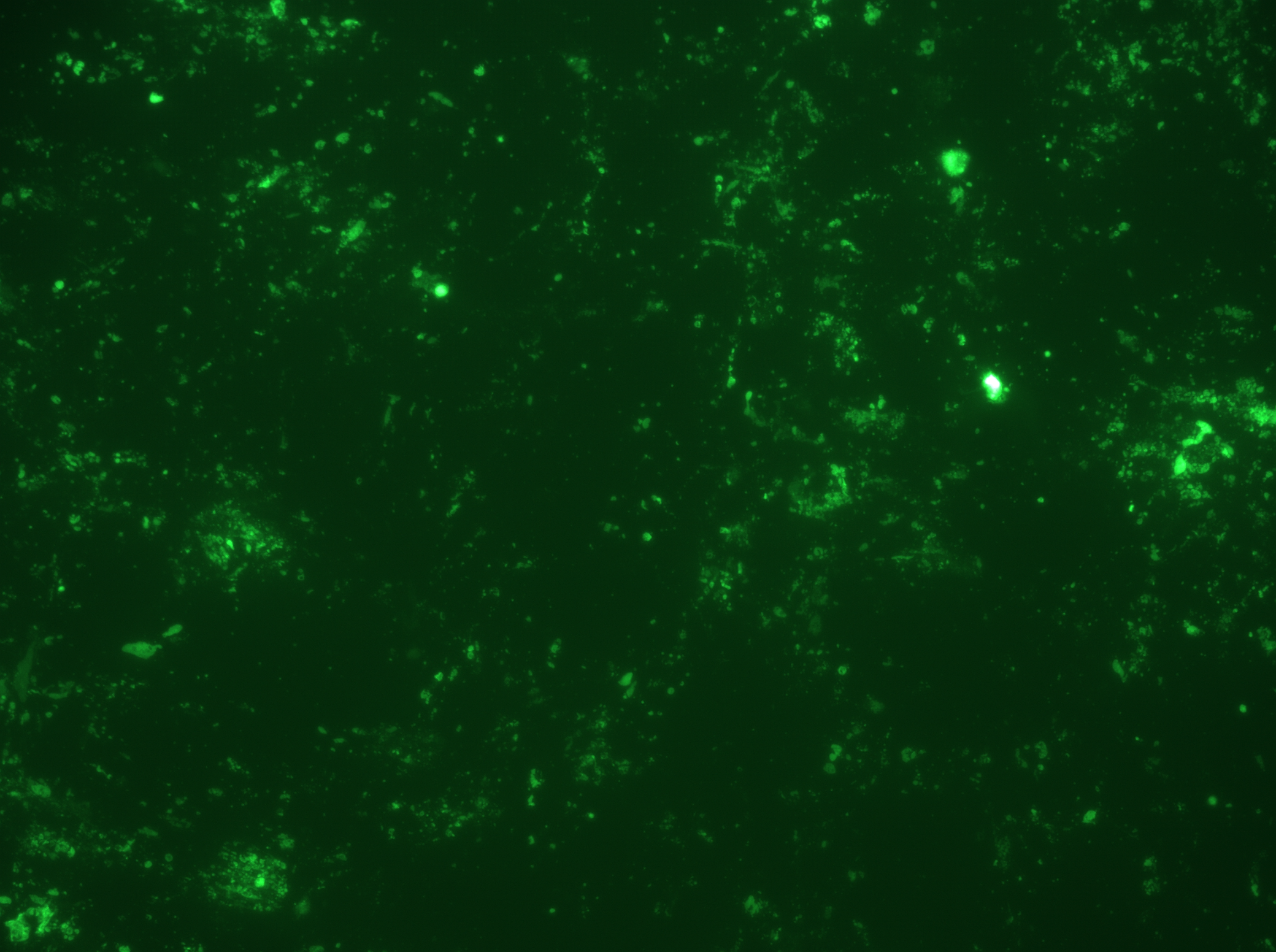

Supplement: Supplementary file 4 — Source data Fig. 3 [file 44318_2024_143_MOESM4_ESM.zip › Figure 3/3D/3D osteoimages/D170 osteo RFP.TIF]

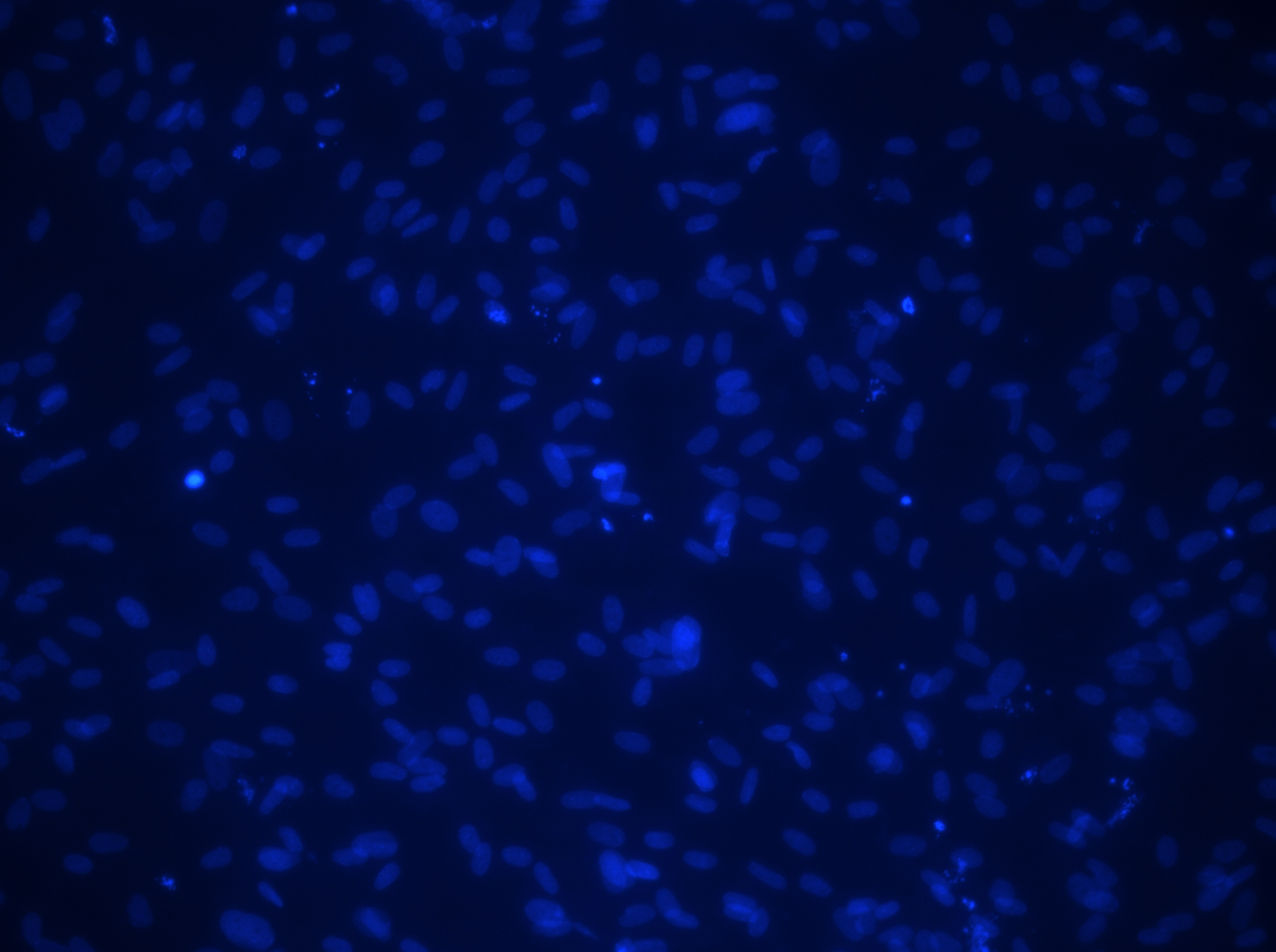

Supplement: Supplementary file 4 — Source data Fig. 3 [file 44318_2024_143_MOESM4_ESM.zip › Figure 3/3D/3D osteoimages/D188 dapi L1.TIF]

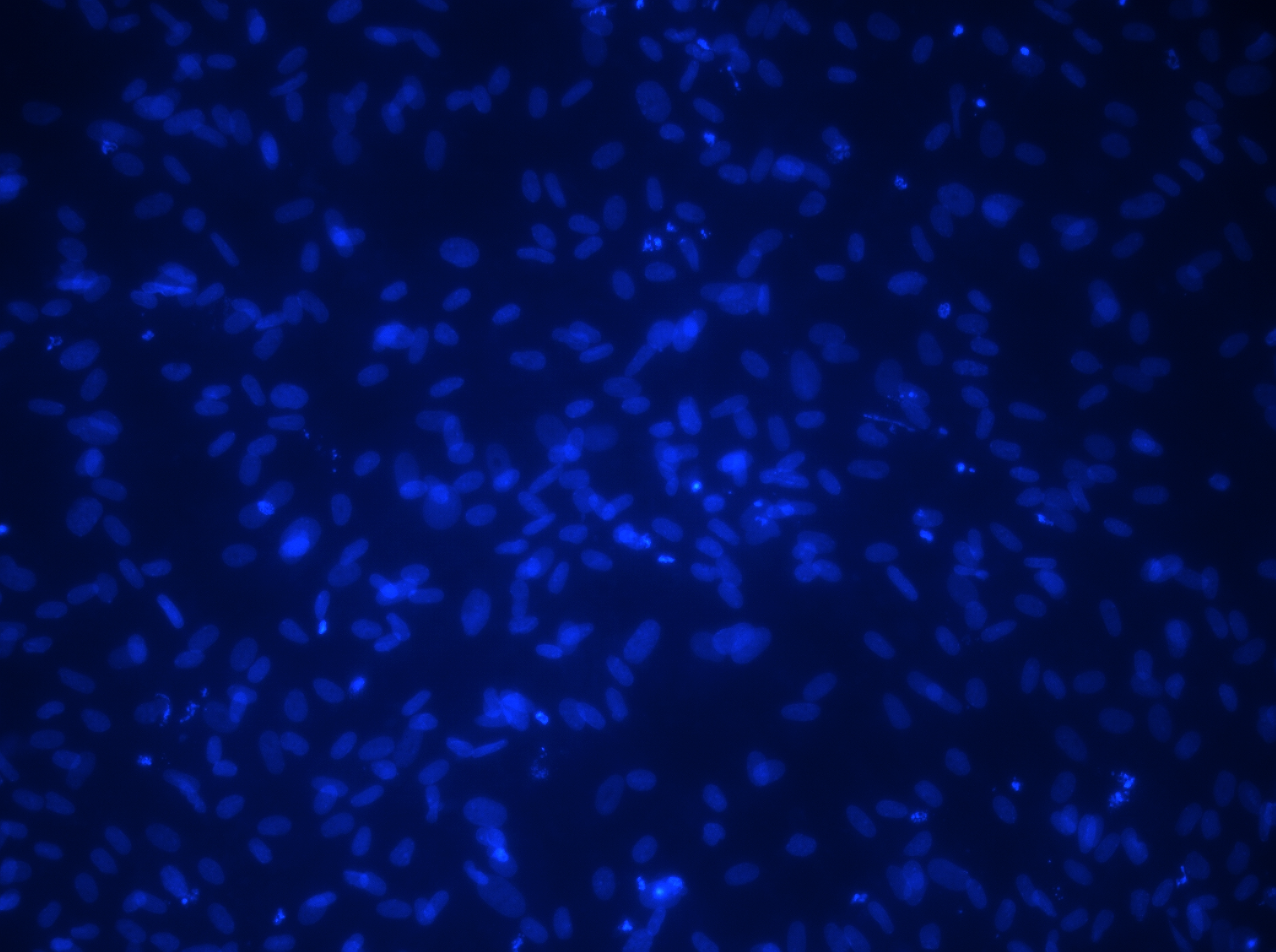

Supplement: Supplementary file 4 — Source data Fig. 3 [file 44318_2024_143_MOESM4_ESM.zip › Figure 3/3D/3D osteoimages/D170 dapi L1.TIF]

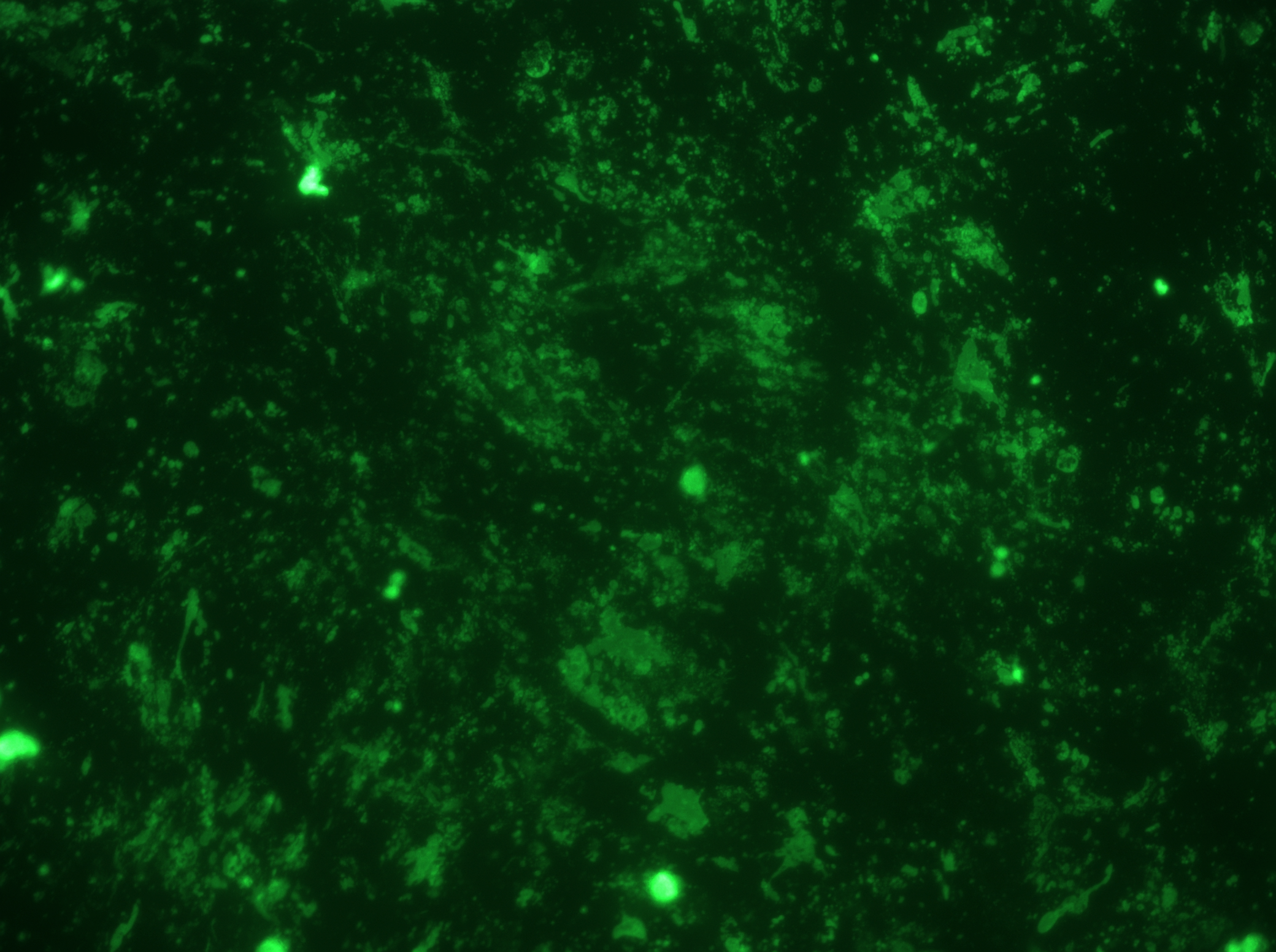

Supplement: Supplementary file 4 — Source data Fig. 3 [file 44318_2024_143_MOESM4_ESM.zip › Figure 3/3D/3D osteoimages/D170 osteo L1.TIF]

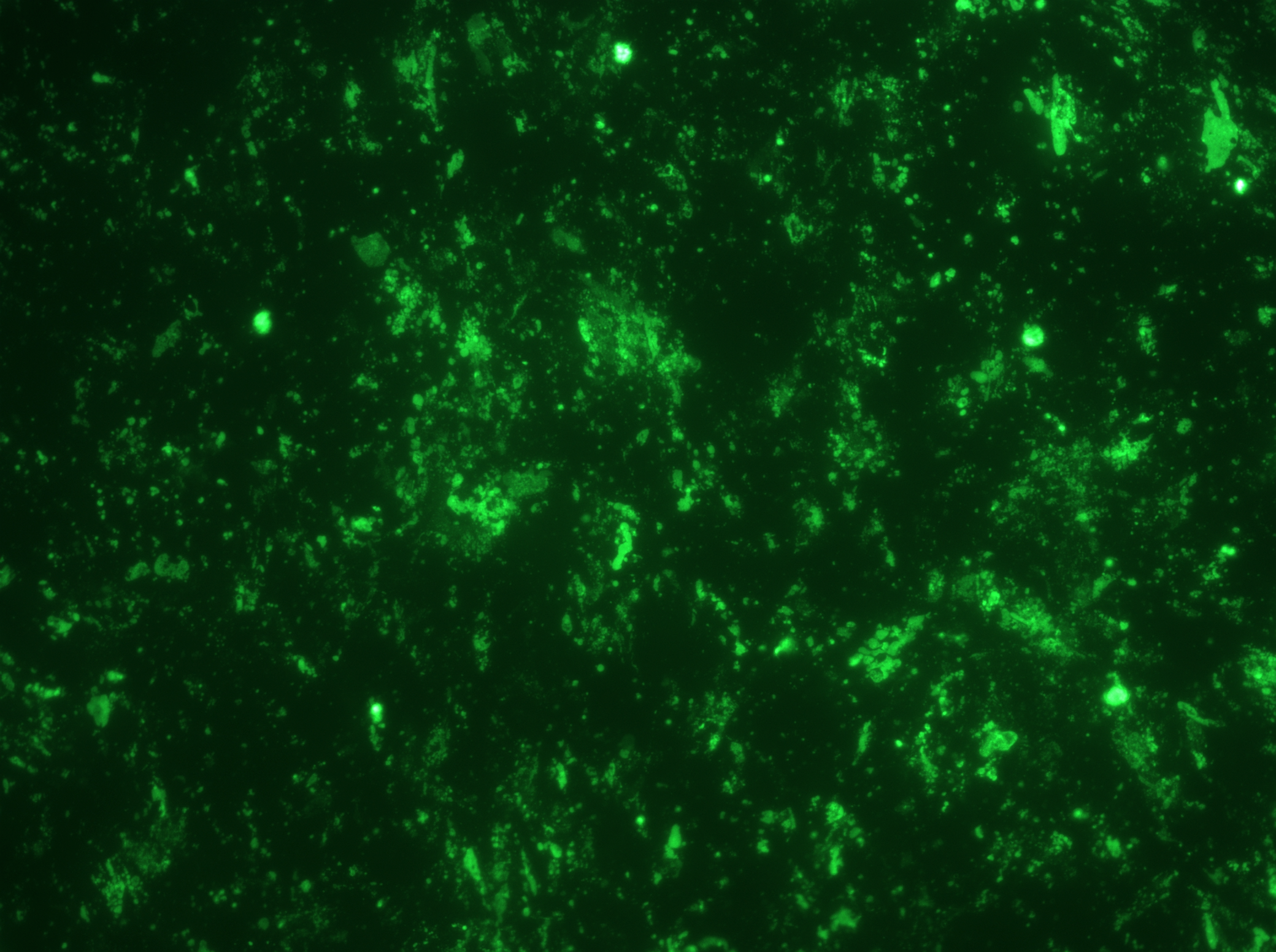

Supplement: Supplementary file 4 — Source data Fig. 3 [file 44318_2024_143_MOESM4_ESM.zip › Figure 3/3D/3D osteoimages/D188 osteo L1.TIF]

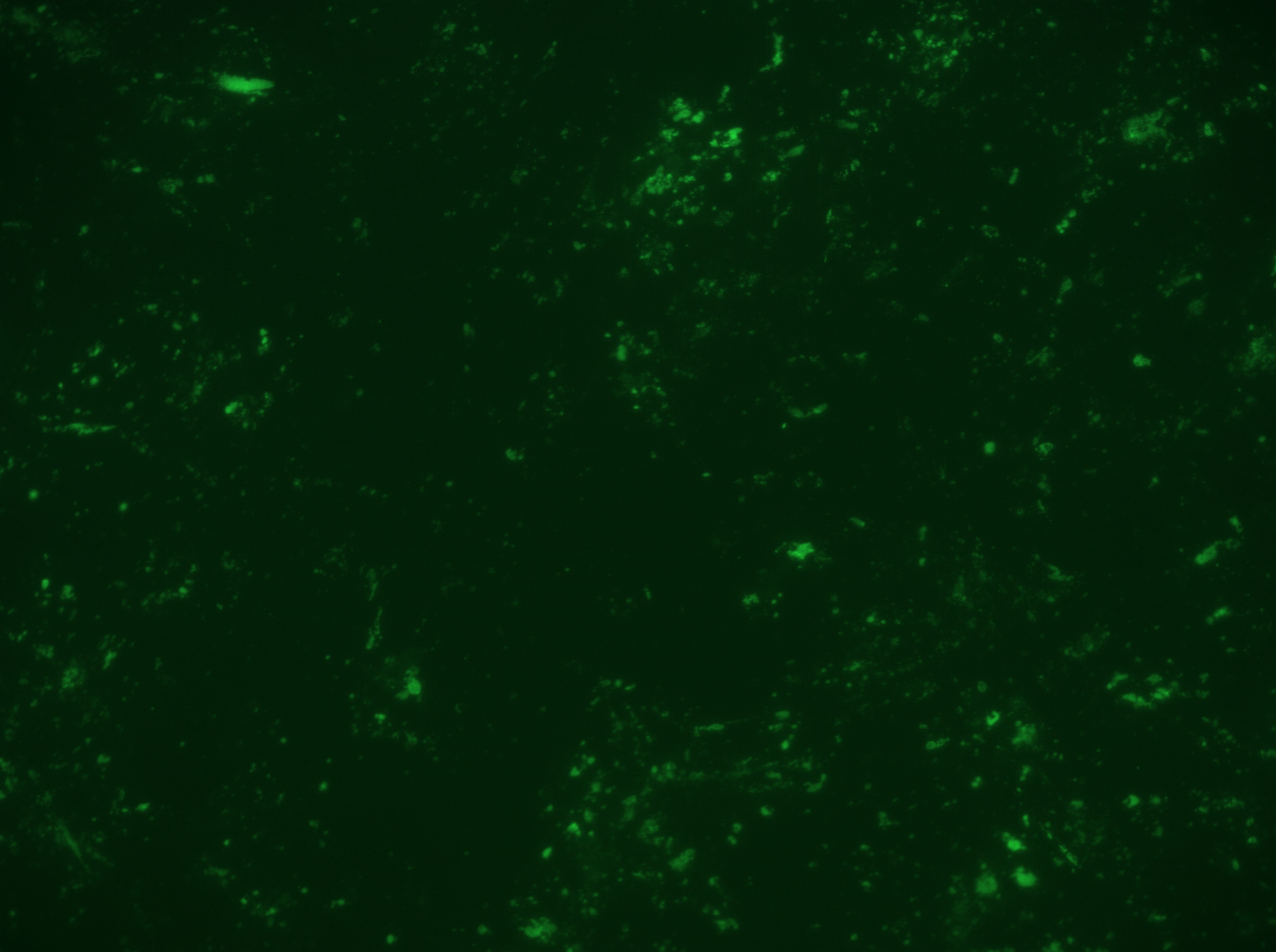

Supplement: Supplementary file 4 — Source data Fig. 3 [file 44318_2024_143_MOESM4_ESM.zip › Figure 3/3D/3D osteoimages/D188 osteo RFP.TIF]

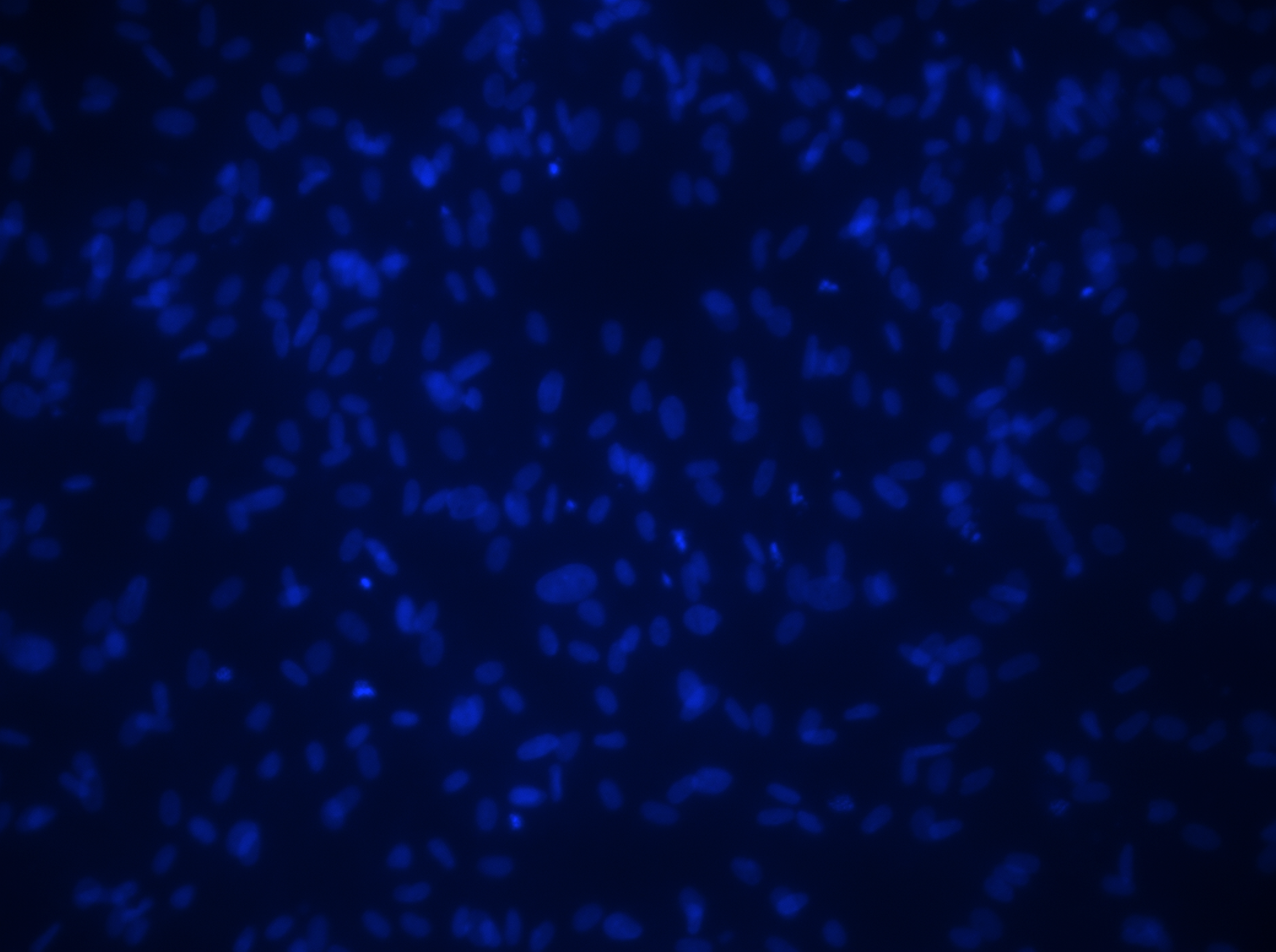

Supplement: Supplementary file 4 — Source data Fig. 3 [file 44318_2024_143_MOESM4_ESM.zip › Figure 3/3D/3D osteoimages/D170 dapi RFP.TIF]

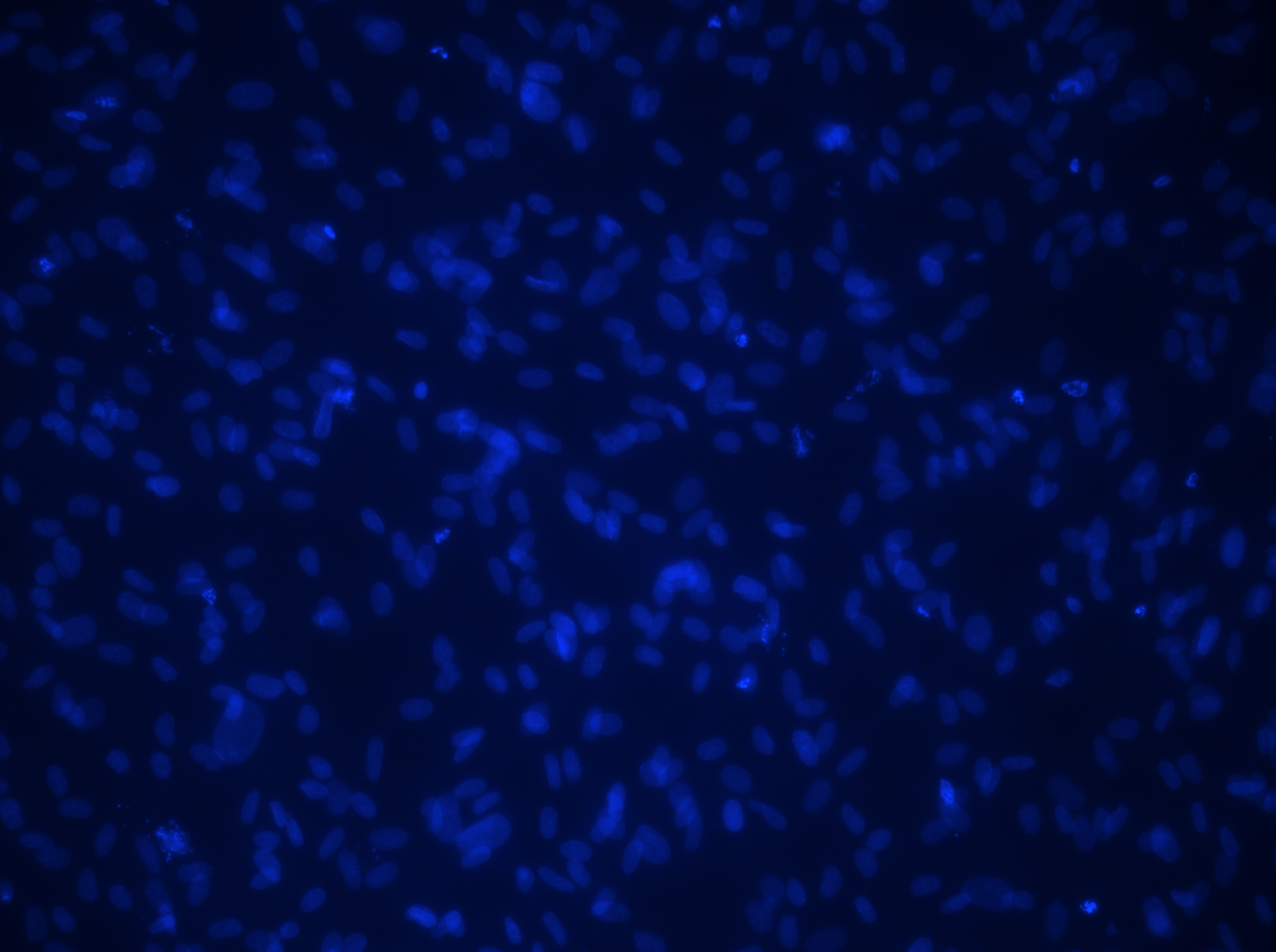

Supplement: Supplementary file 4 — Source data Fig. 3 [file 44318_2024_143_MOESM4_ESM.zip › Figure 3/3D/3D osteoimages/D188 dapi RFP.TIF]

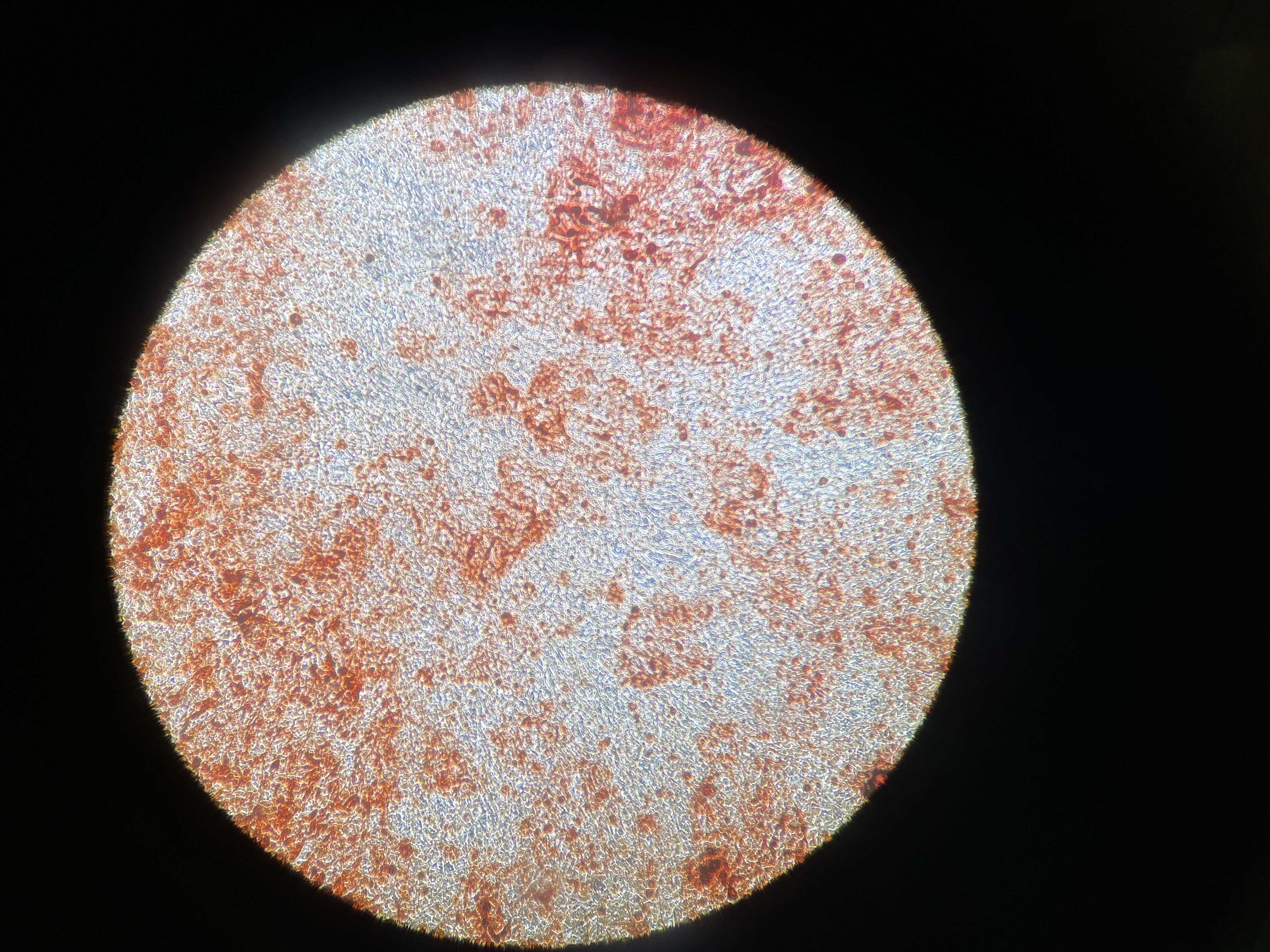

Supplement: Supplementary file 4 — Source data Fig. 3 [file 44318_2024_143_MOESM4_ESM.zip › Figure 3/3D/3D Alizarin images/D170 RFP.jpg]

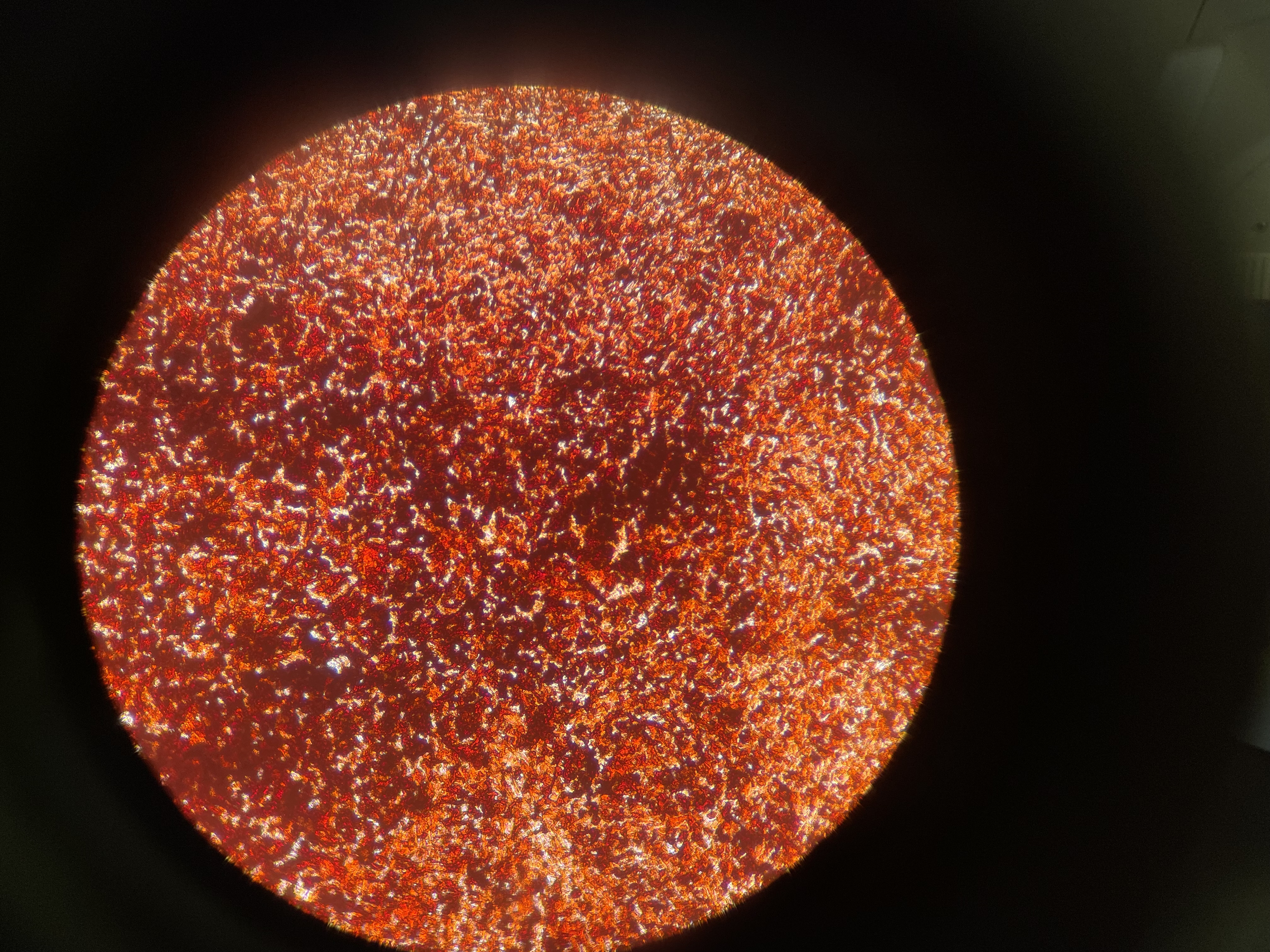

Supplement: Supplementary file 4 — Source data Fig. 3 [file 44318_2024_143_MOESM4_ESM.zip › Figure 3/3D/3D Alizarin images/D188 L1.jpg]

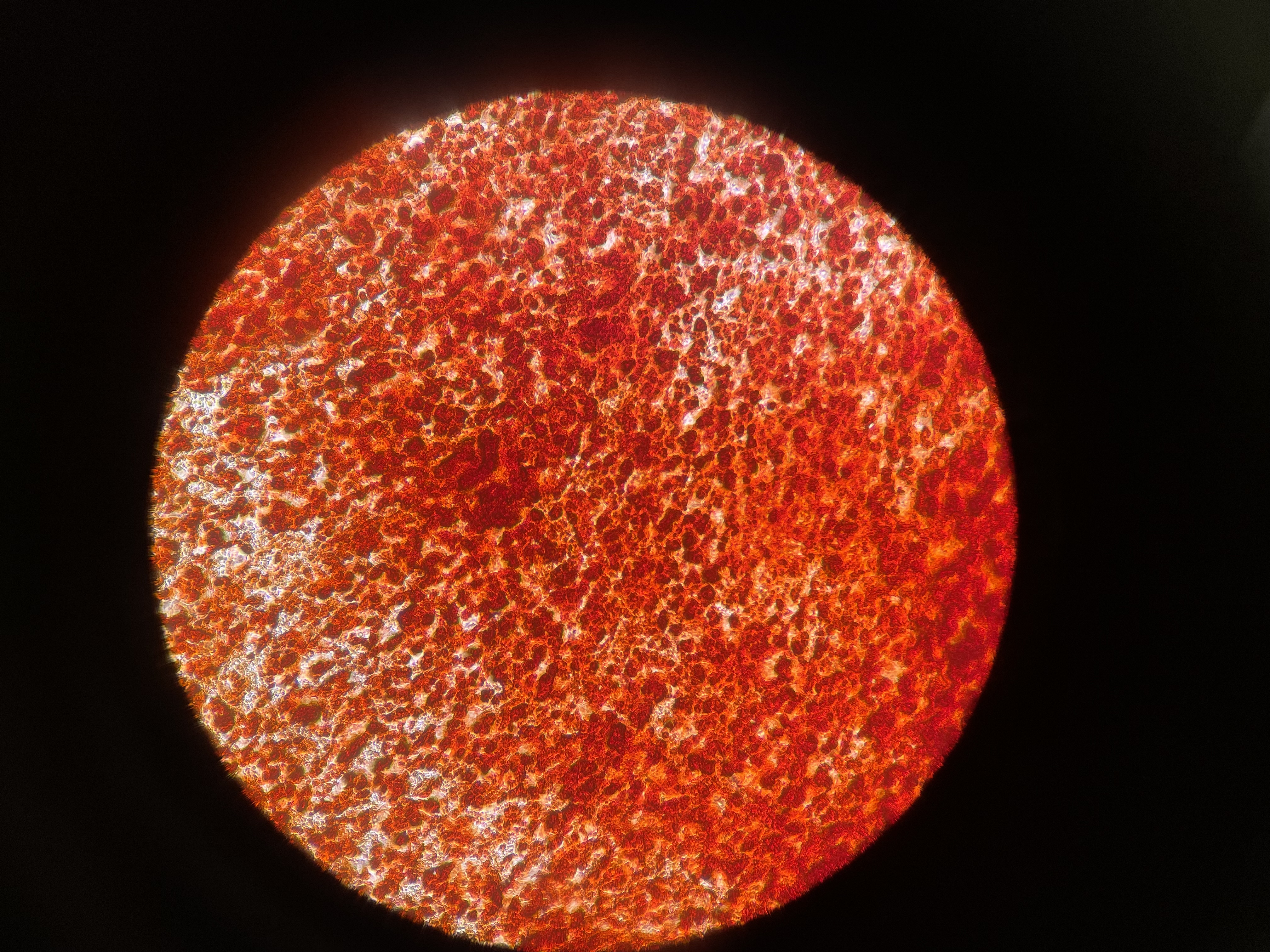

Supplement: Supplementary file 4 — Source data Fig. 3 [file 44318_2024_143_MOESM4_ESM.zip › Figure 3/3D/3D Alizarin images/D170 L1.jpg]

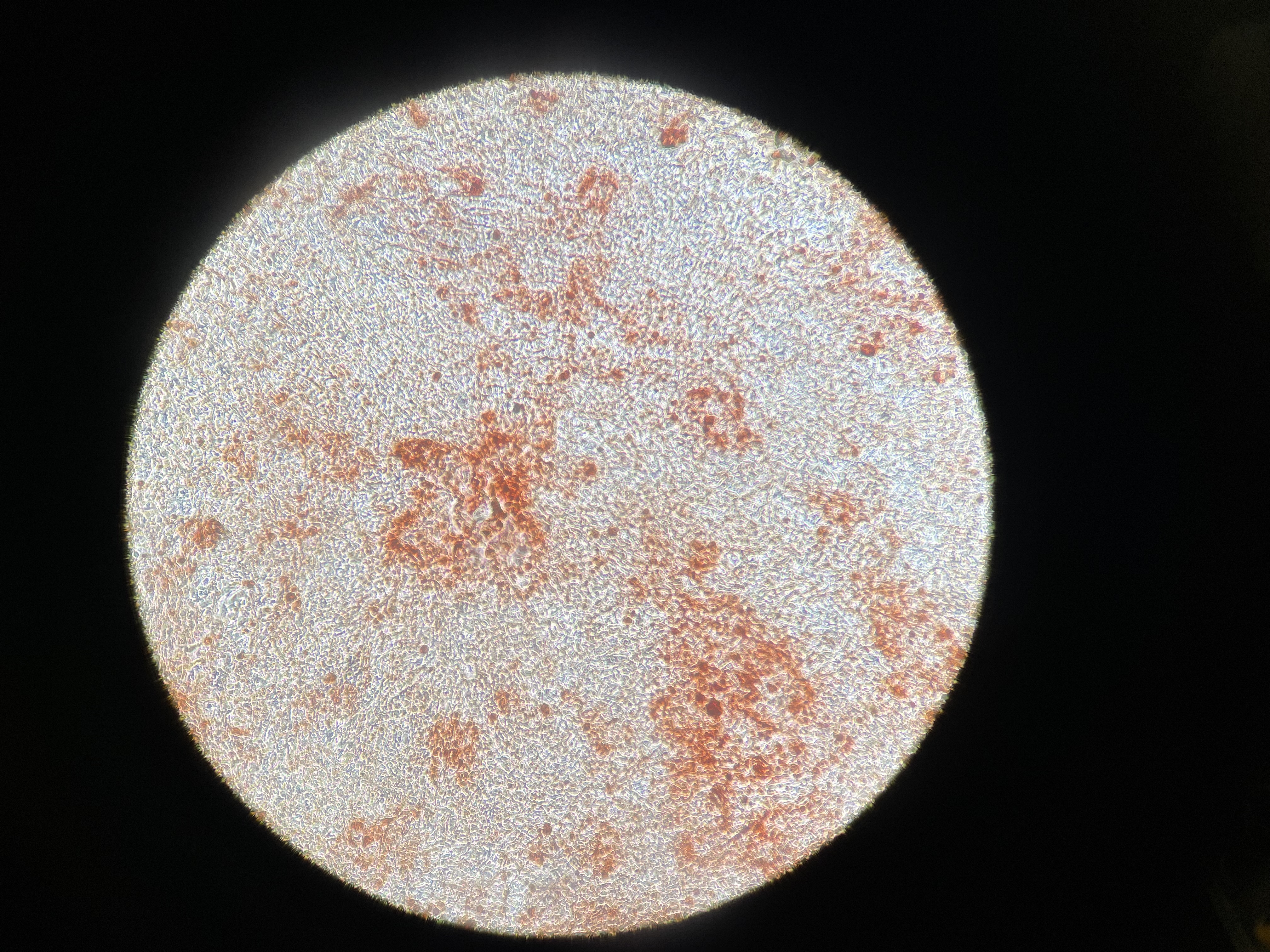

Supplement: Supplementary file 4 — Source data Fig. 3 [file 44318_2024_143_MOESM4_ESM.zip › Figure 3/3D/3D Alizarin images/D188 RFP.jpg]

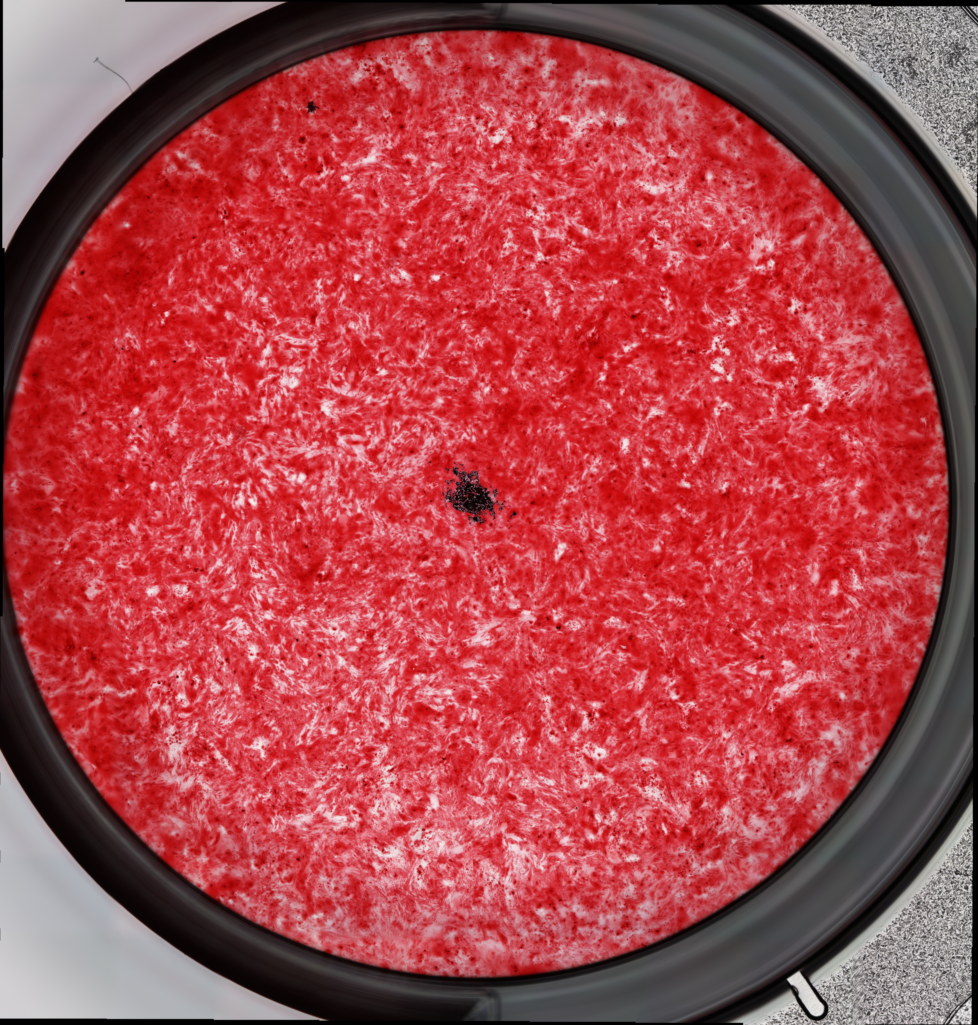

Supplement: Supplementary file 4 — Source data Fig. 3 [file 44318_2024_143_MOESM4_ESM.zip › Figure 3/3F/3F micr image/HUK7_d21.png]

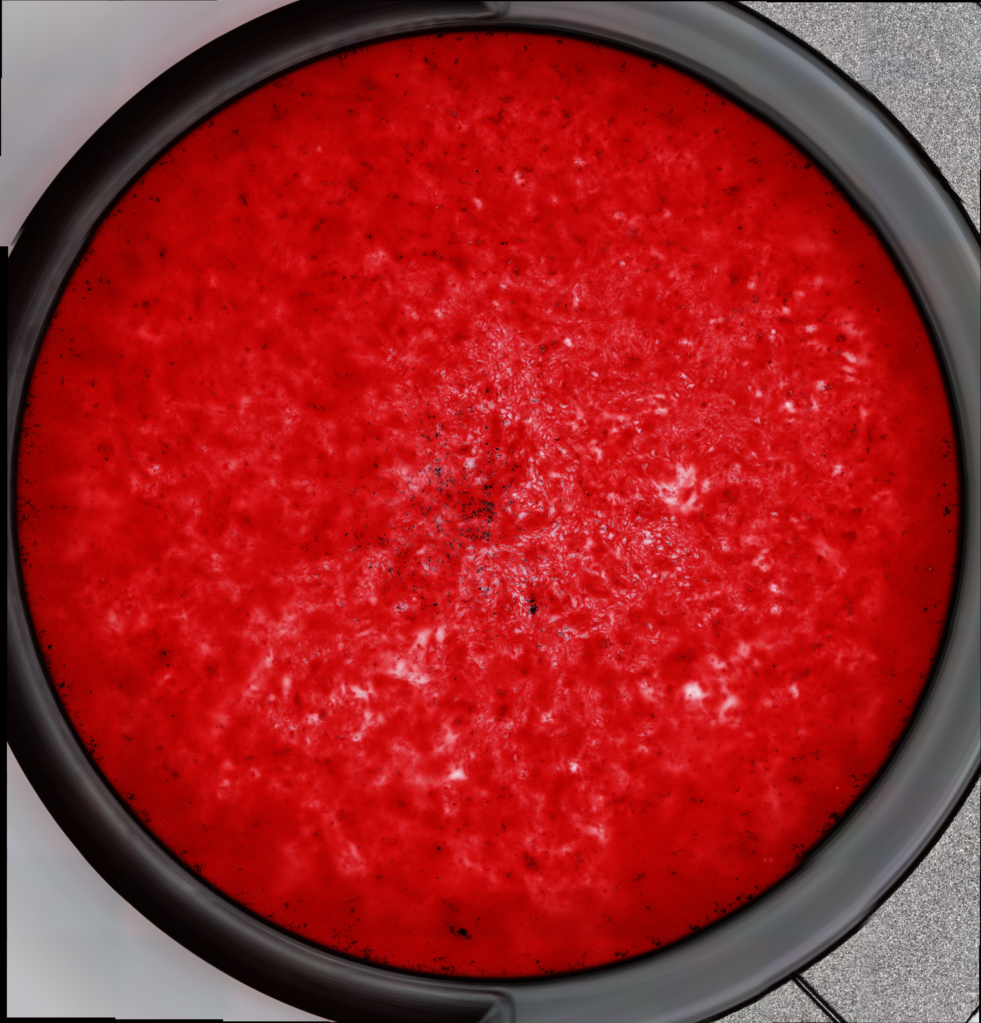

Supplement: Supplementary file 4 — Source data Fig. 3 [file 44318_2024_143_MOESM4_ESM.zip › Figure 3/3F/3F micr image/D188_d21.png]

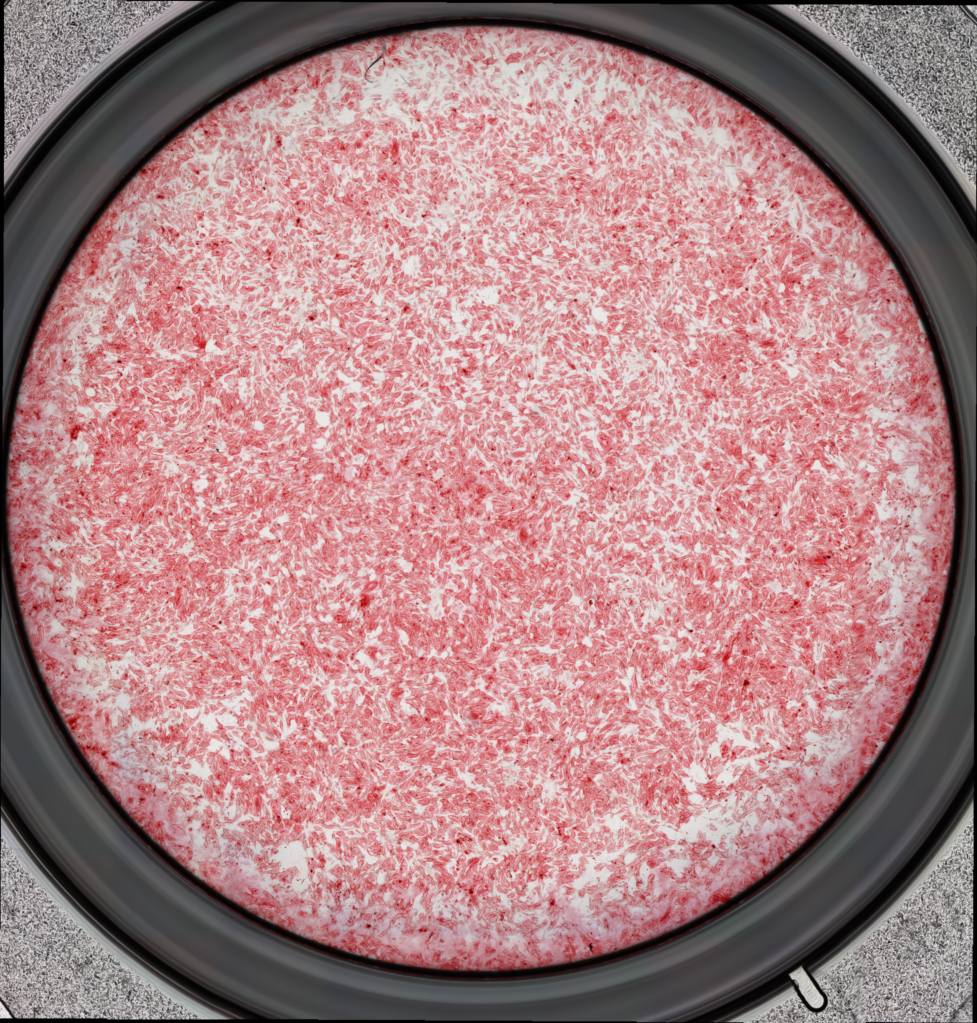

Supplement: Supplementary file 4 — Source data Fig. 3 [file 44318_2024_143_MOESM4_ESM.zip › Figure 3/3F/3F micr image/HUK12_d14.png]

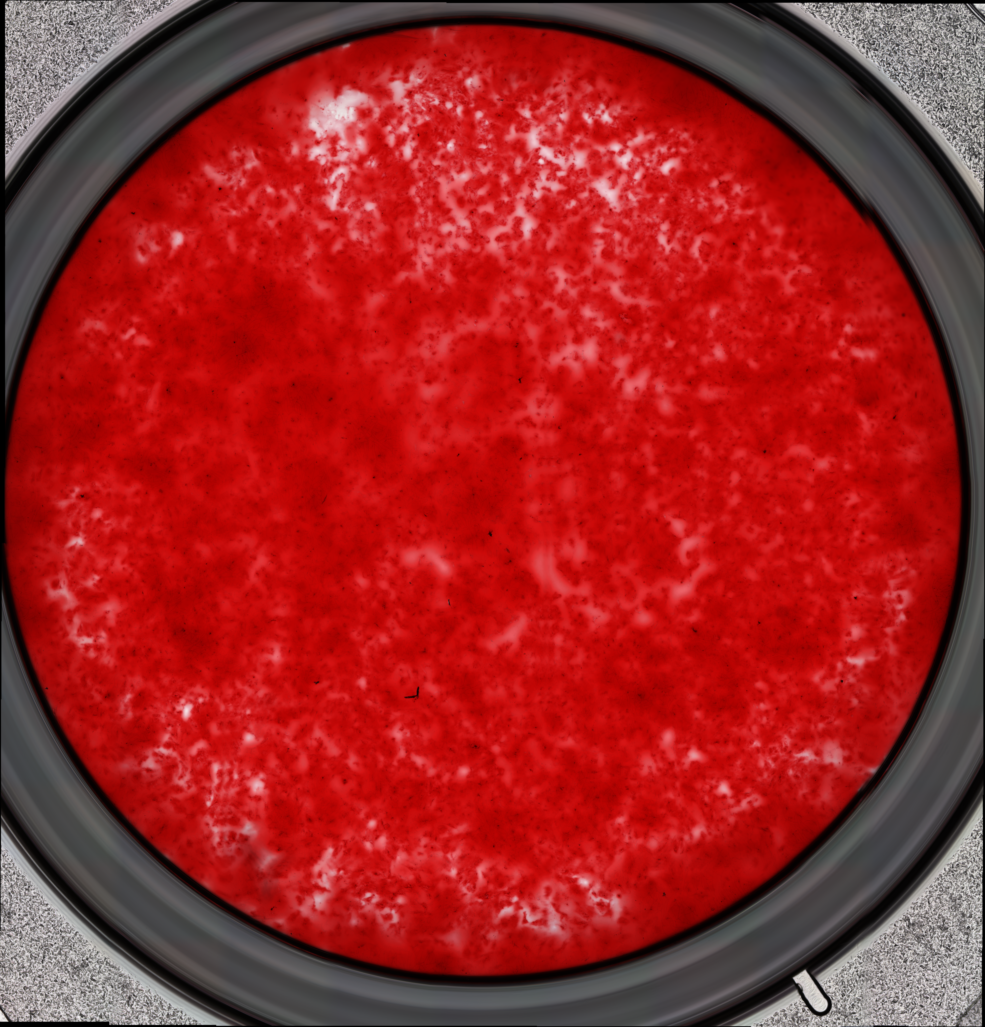

Supplement: Supplementary file 4 — Source data Fig. 3 [file 44318_2024_143_MOESM4_ESM.zip › Figure 3/3F/3F micr image/D247_d21.png]

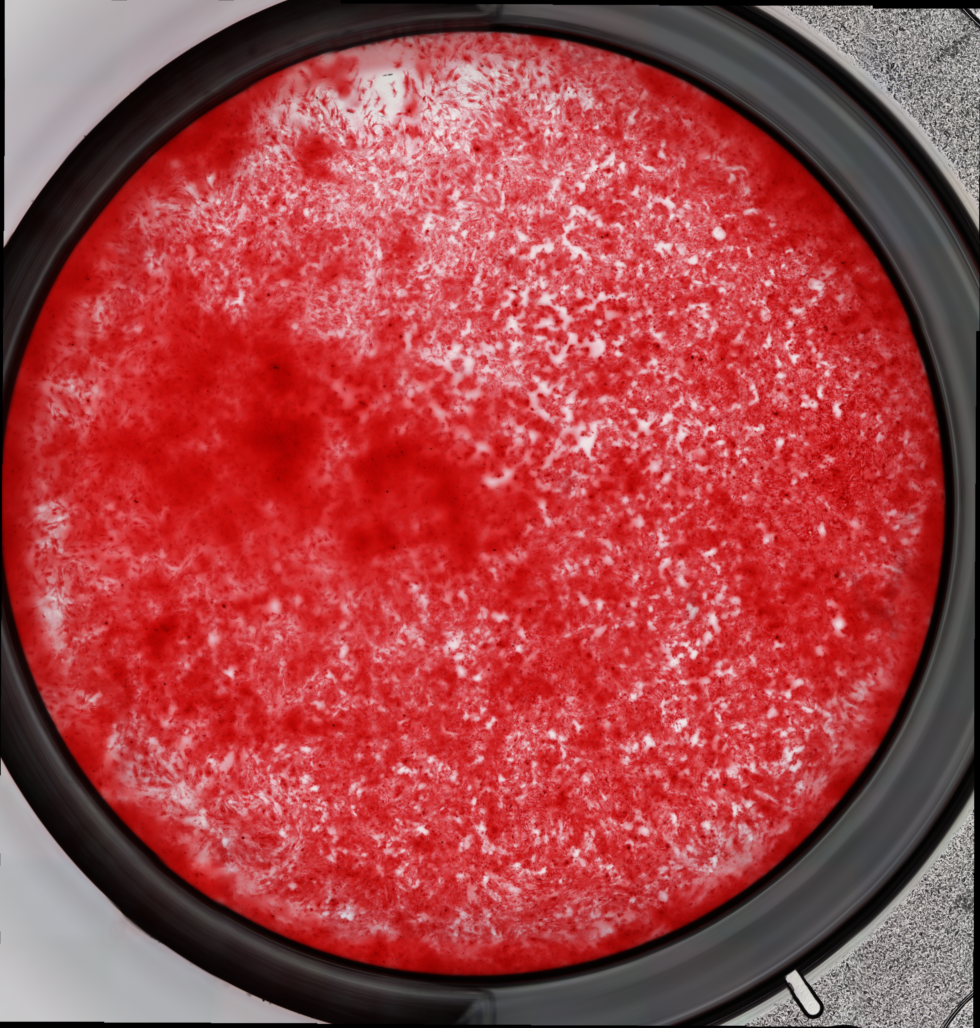

Supplement: Supplementary file 4 — Source data Fig. 3 [file 44318_2024_143_MOESM4_ESM.zip › Figure 3/3F/3F micr image/HUK16_d21.png]

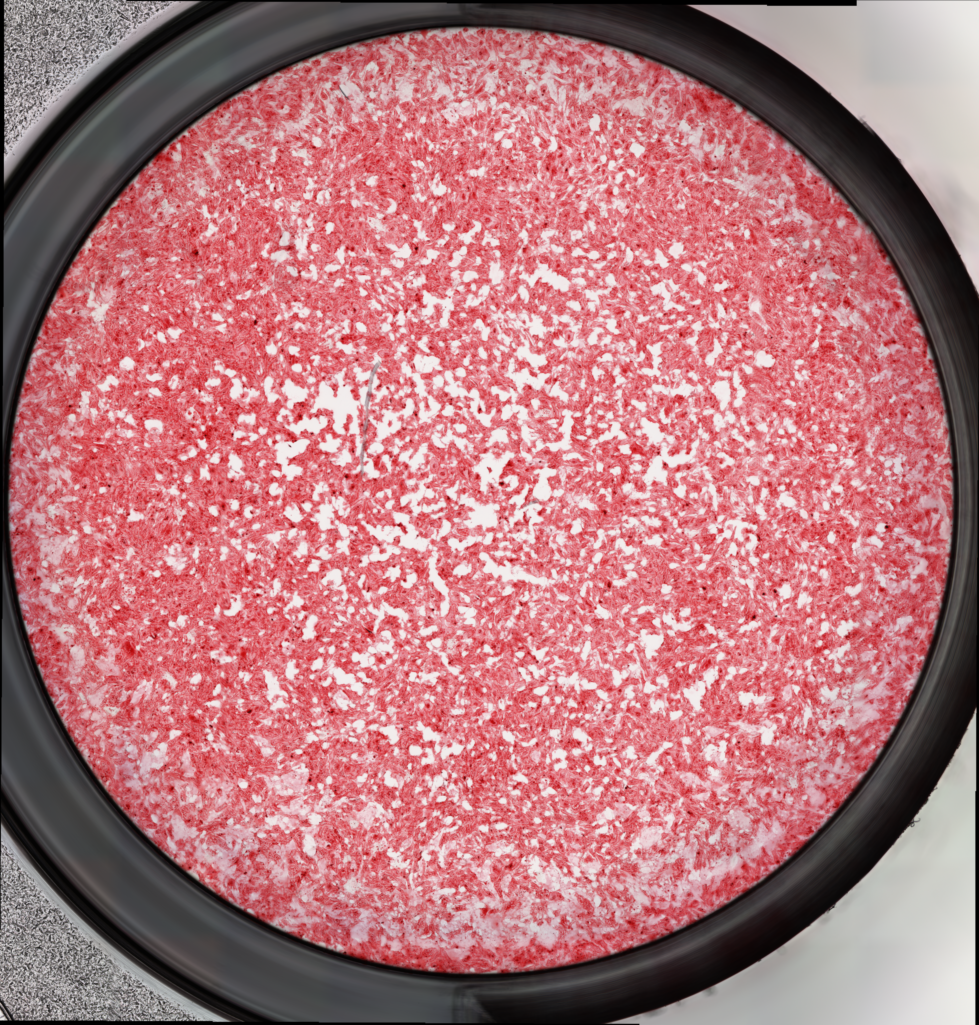

Supplement: Supplementary file 4 — Source data Fig. 3 [file 44318_2024_143_MOESM4_ESM.zip › Figure 3/3F/3F micr image/HUK12_d17.png]

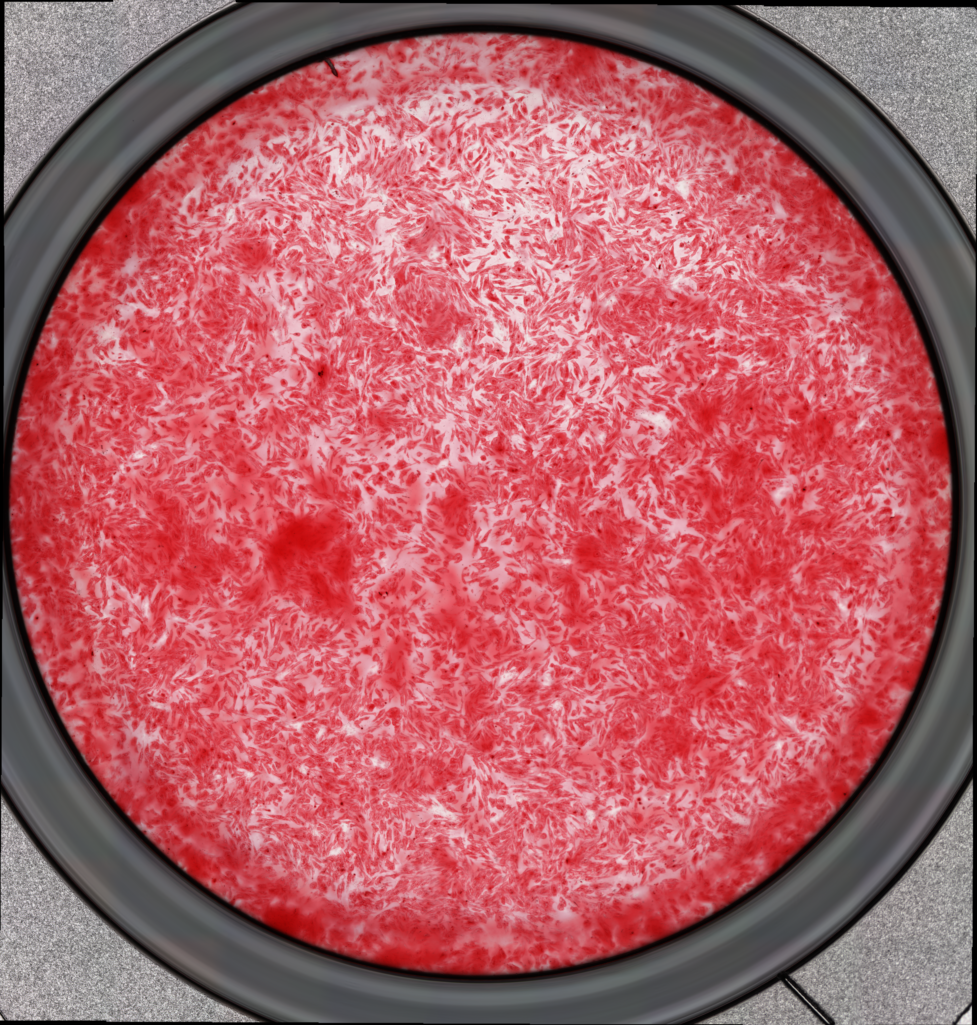

Supplement: Supplementary file 4 — Source data Fig. 3 [file 44318_2024_143_MOESM4_ESM.zip › Figure 3/3F/3F micr image/D170_d14.png]

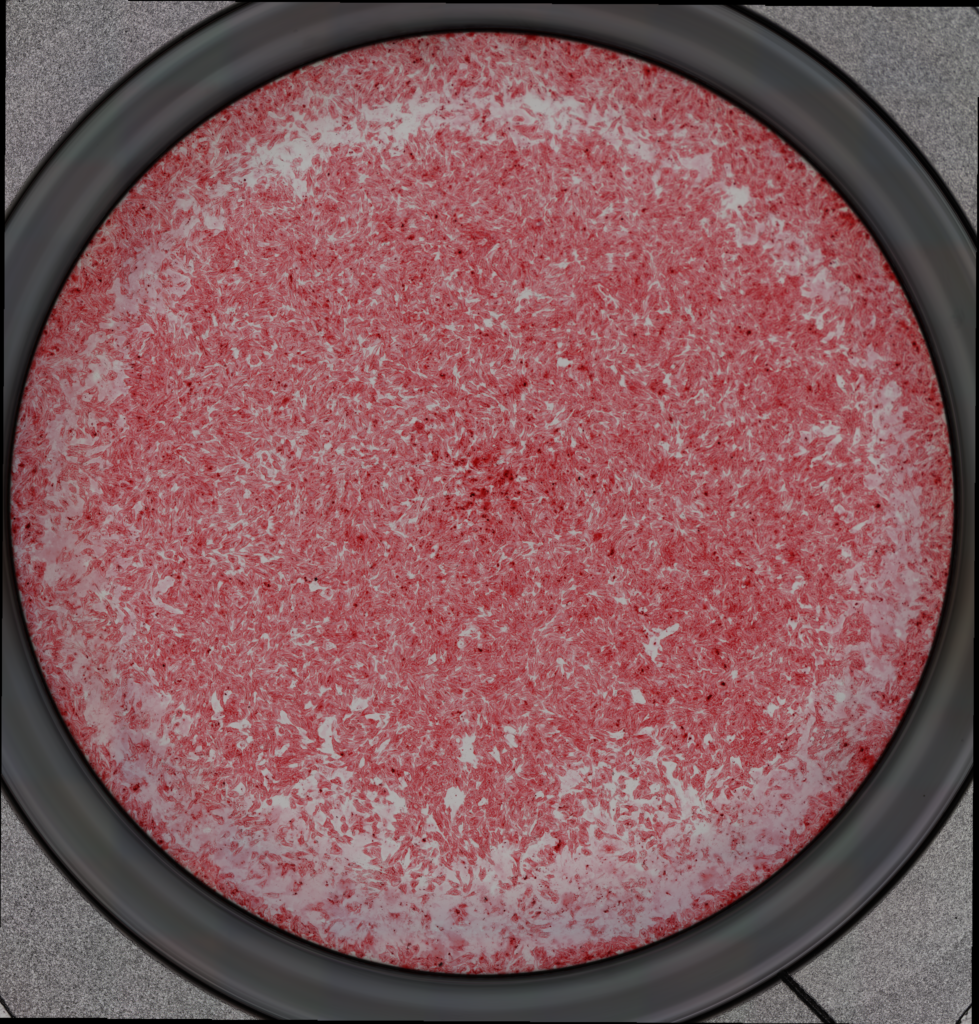

Supplement: Supplementary file 4 — Source data Fig. 3 [file 44318_2024_143_MOESM4_ESM.zip › Figure 3/3F/3F micr image/HUK9_d17.png]

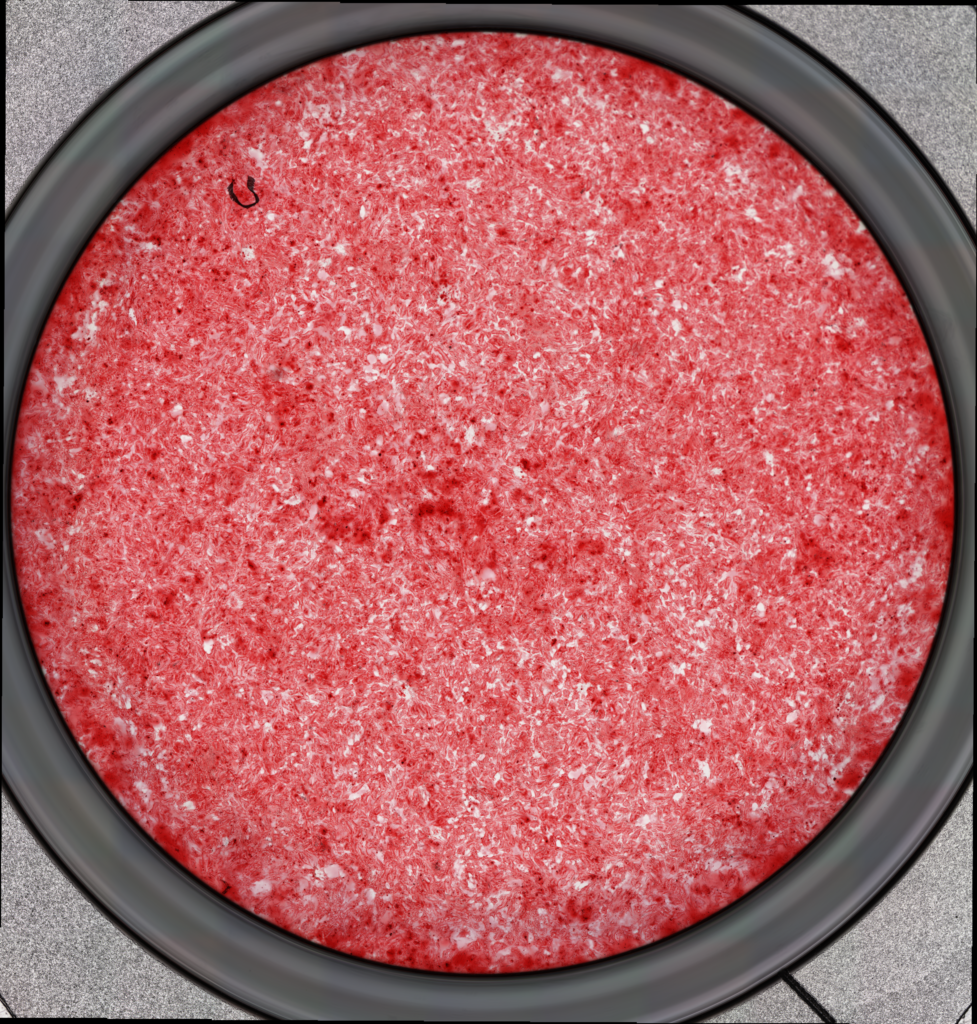

Supplement: Supplementary file 4 — Source data Fig. 3 [file 44318_2024_143_MOESM4_ESM.zip › Figure 3/3F/3F micr image/D239_d14.png]

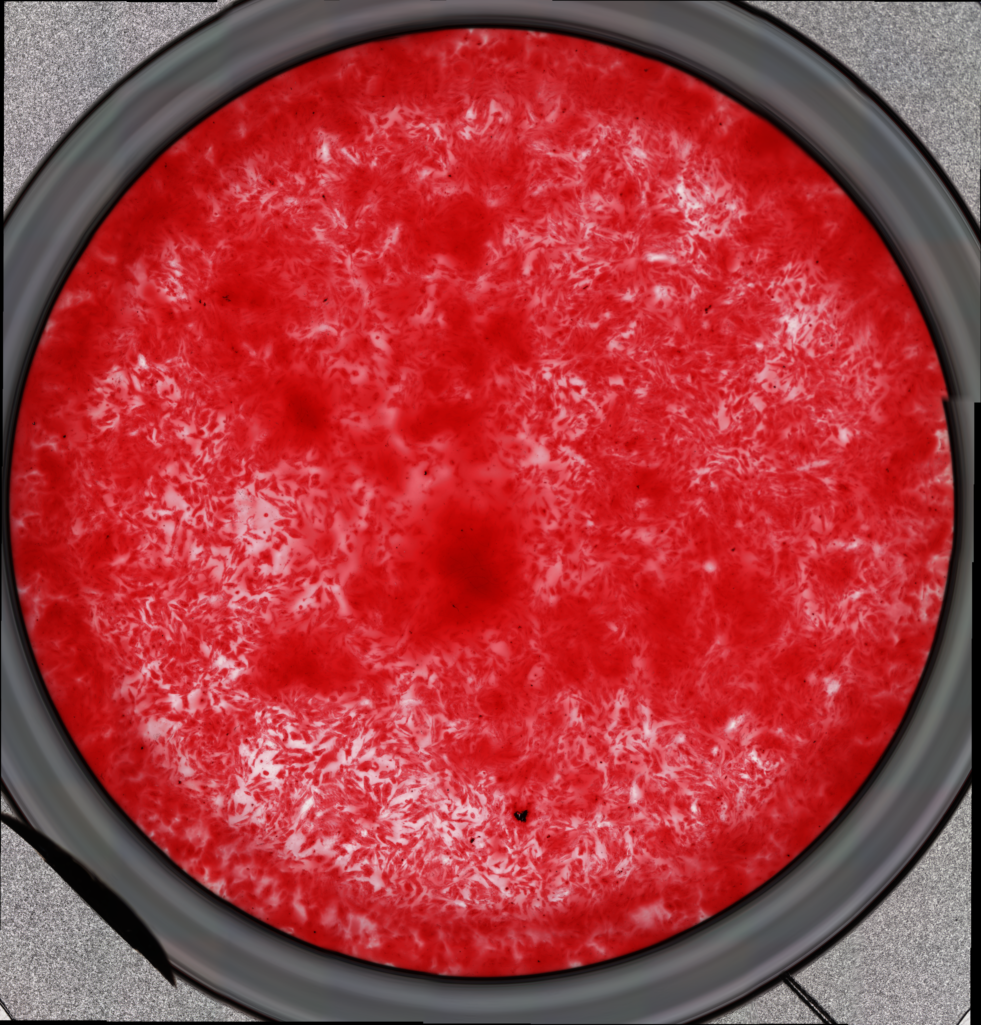

Supplement: Supplementary file 4 — Source data Fig. 3 [file 44318_2024_143_MOESM4_ESM.zip › Figure 3/3F/3F micr image/D170_d17.png]

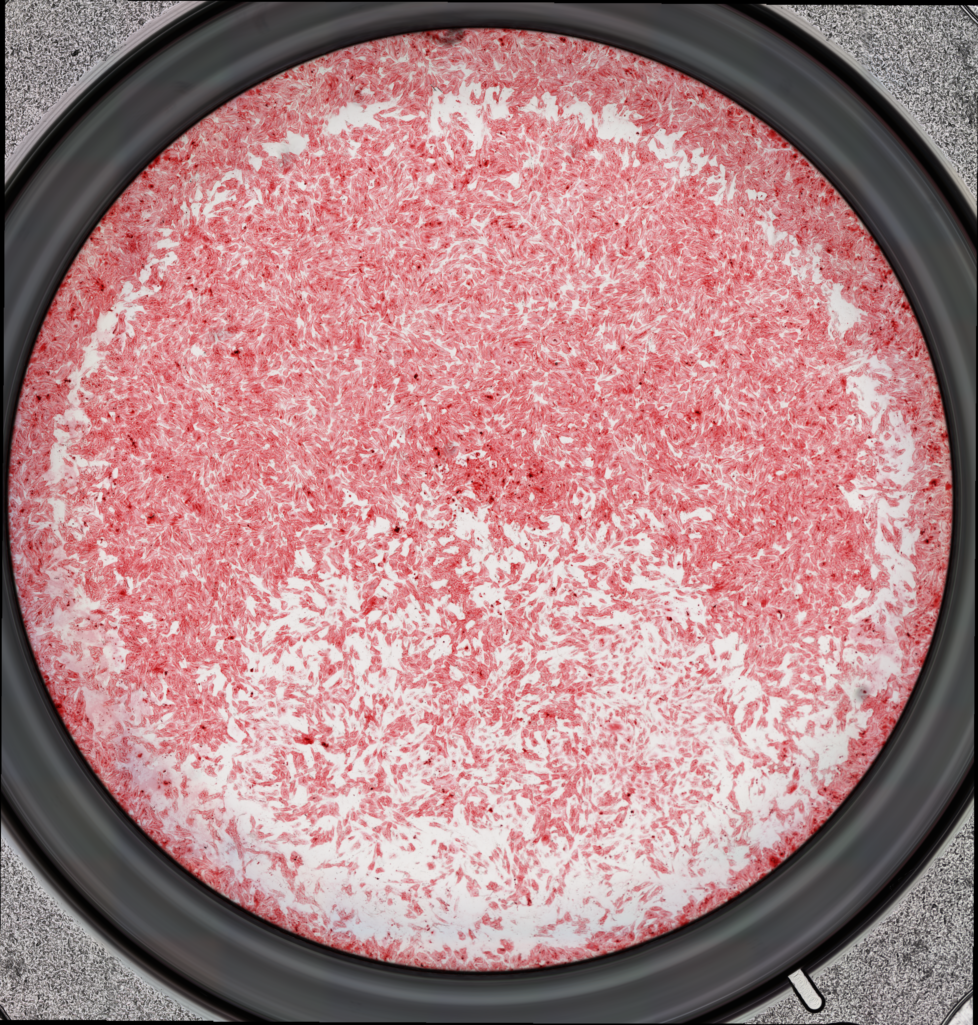

Supplement: Supplementary file 4 — Source data Fig. 3 [file 44318_2024_143_MOESM4_ESM.zip › Figure 3/3F/3F micr image/HUK9_d14.png]

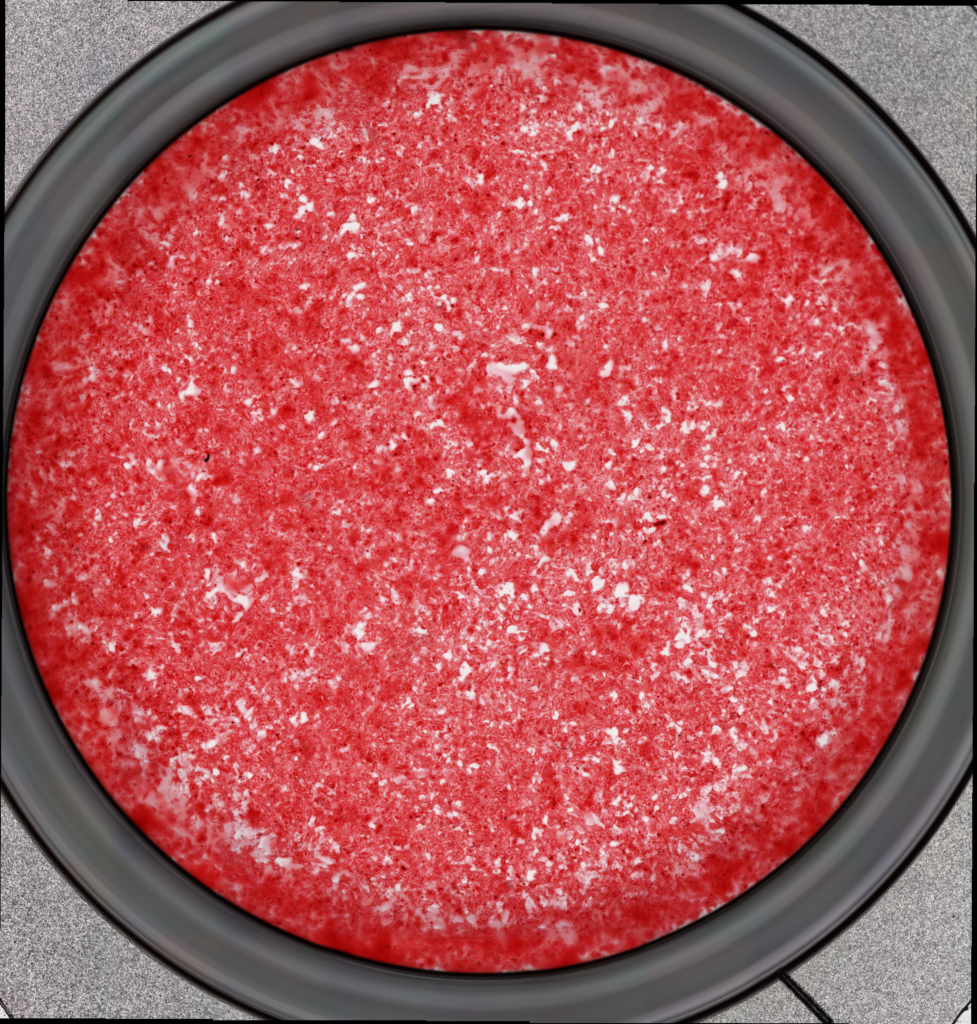

Supplement: Supplementary file 4 — Source data Fig. 3 [file 44318_2024_143_MOESM4_ESM.zip › Figure 3/3F/3F micr image/D239_d17.png]

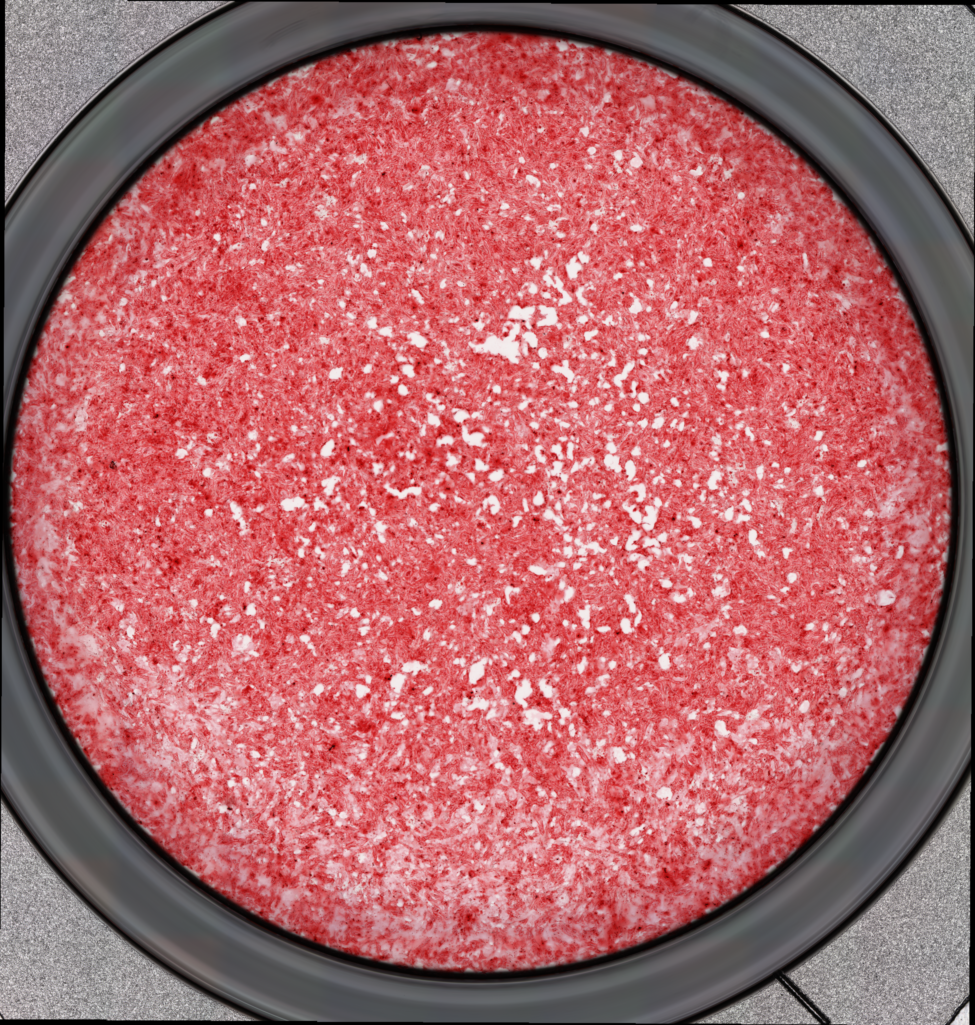

Supplement: Supplementary file 4 — Source data Fig. 3 [file 44318_2024_143_MOESM4_ESM.zip › Figure 3/3F/3F micr image/HUK12_d21.png]

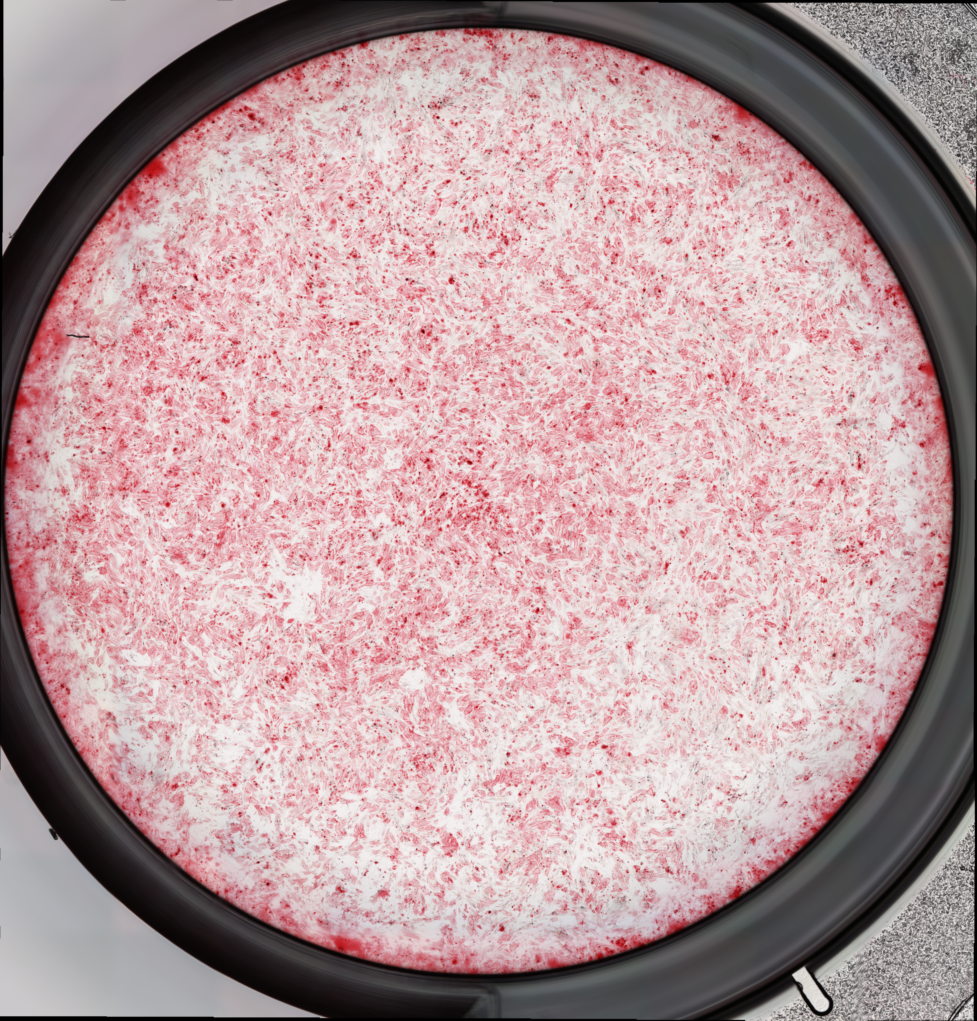

Supplement: Supplementary file 4 — Source data Fig. 3 [file 44318_2024_143_MOESM4_ESM.zip › Figure 3/3F/3F micr image/D188_d14.png]

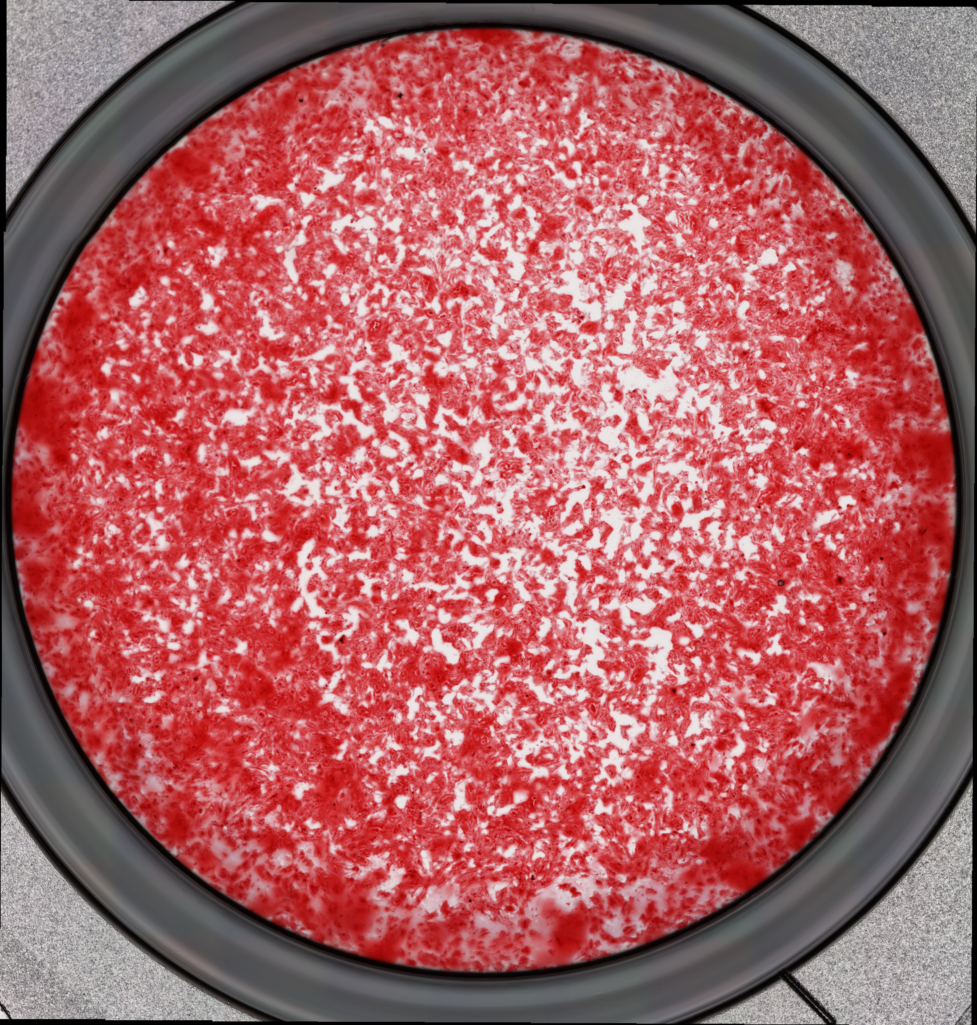

Supplement: Supplementary file 4 — Source data Fig. 3 [file 44318_2024_143_MOESM4_ESM.zip › Figure 3/3F/3F micr image/D247_d14.png]

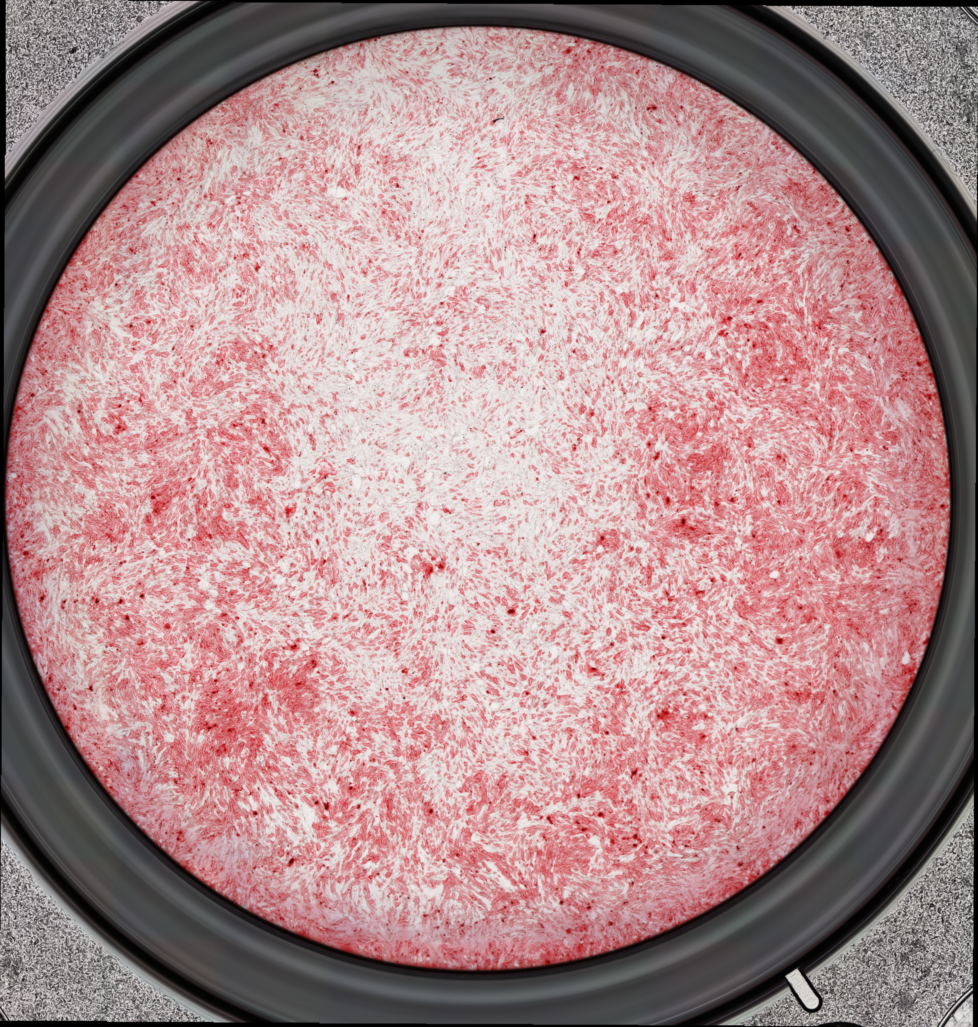

Supplement: Supplementary file 4 — Source data Fig. 3 [file 44318_2024_143_MOESM4_ESM.zip › Figure 3/3F/3F micr image/HUK16_d14.png]

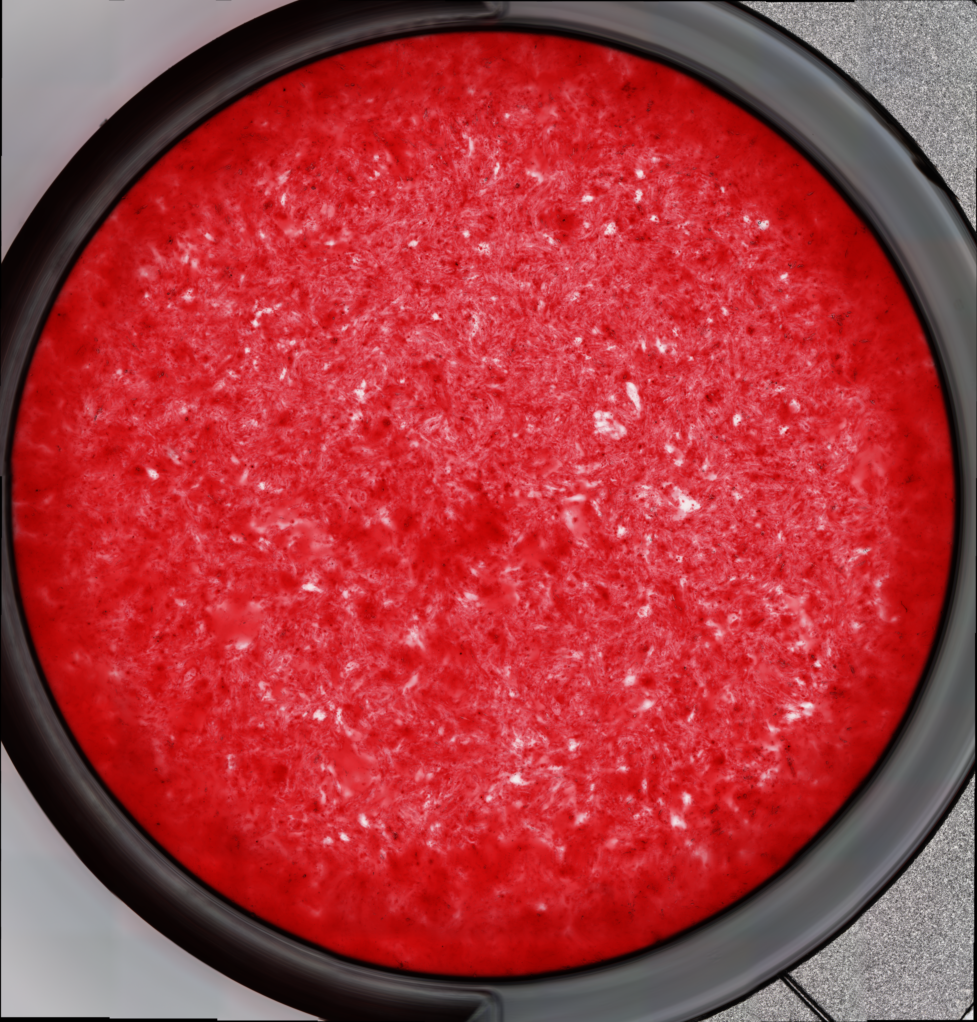

Supplement: Supplementary file 4 — Source data Fig. 3 [file 44318_2024_143_MOESM4_ESM.zip › Figure 3/3F/3F micr image/D188_d17.png]

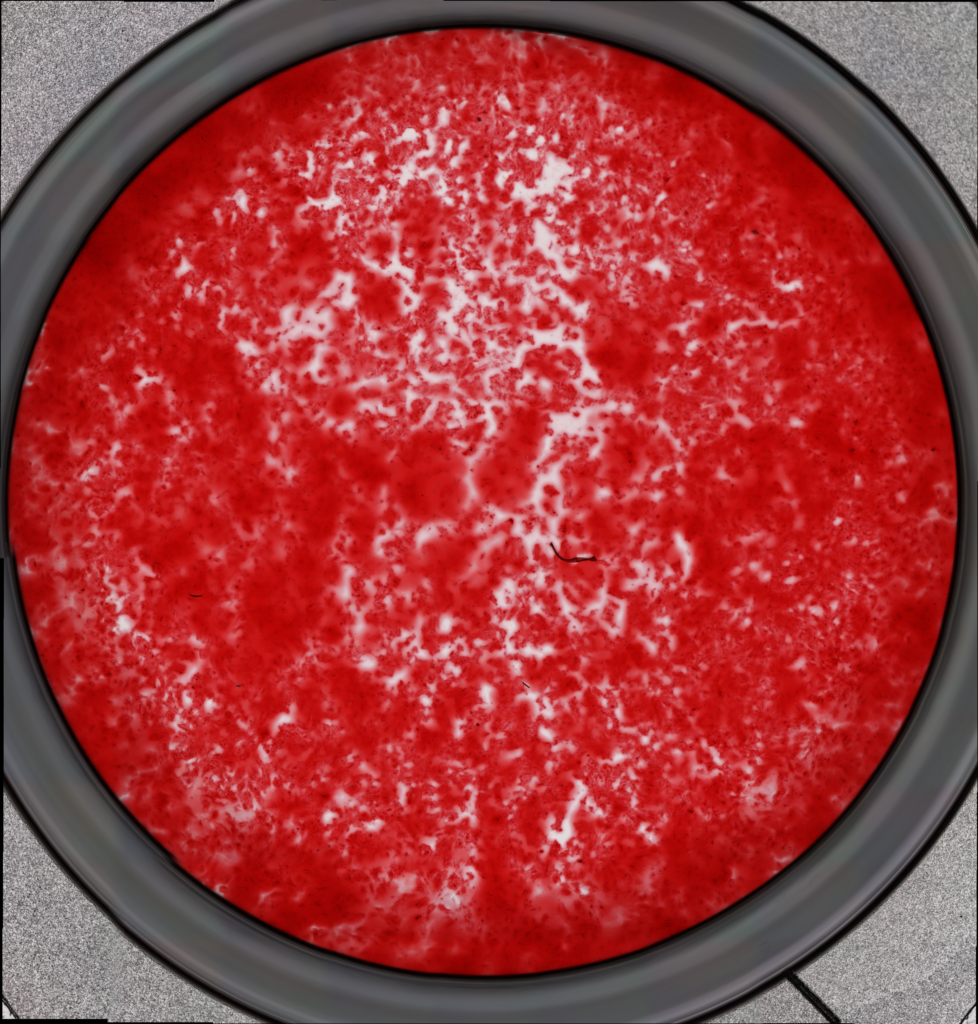

Supplement: Supplementary file 4 — Source data Fig. 3 [file 44318_2024_143_MOESM4_ESM.zip › Figure 3/3F/3F micr image/D247_d17.png]

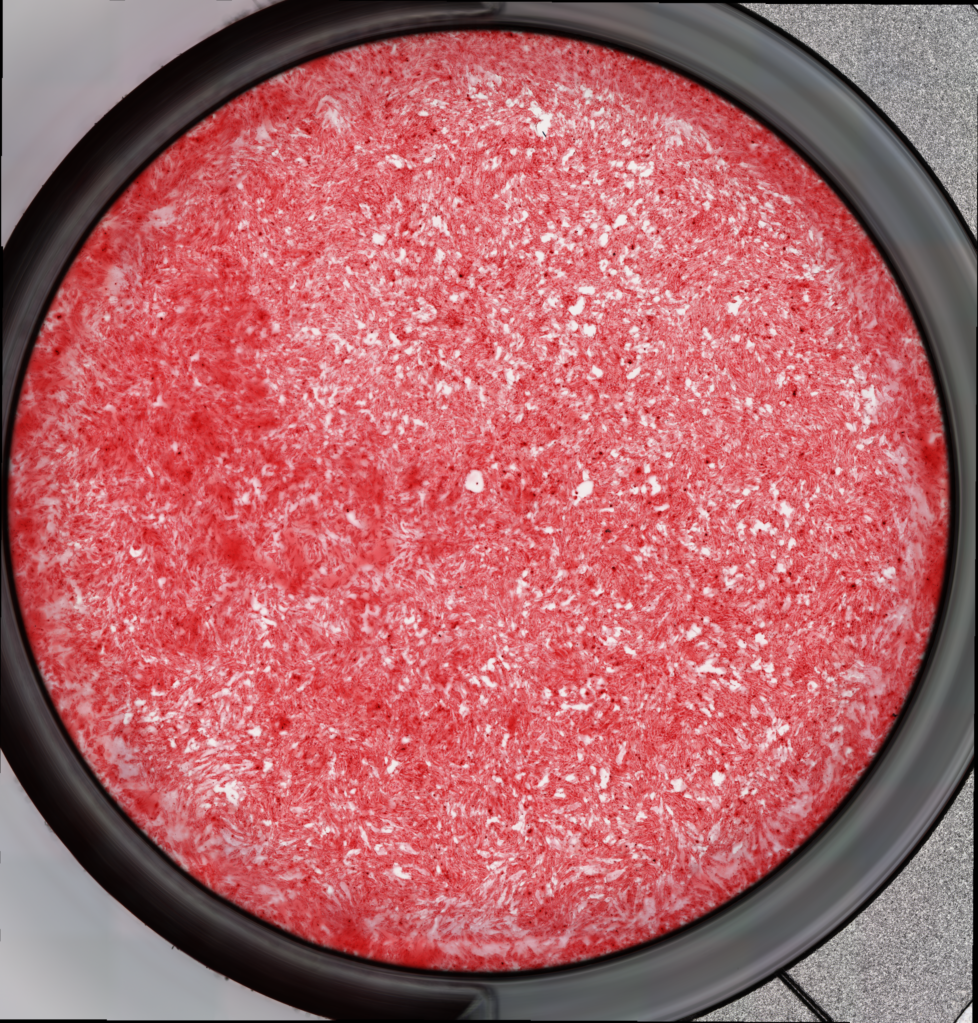

Supplement: Supplementary file 4 — Source data Fig. 3 [file 44318_2024_143_MOESM4_ESM.zip › Figure 3/3F/3F micr image/HUK16_d17.png]

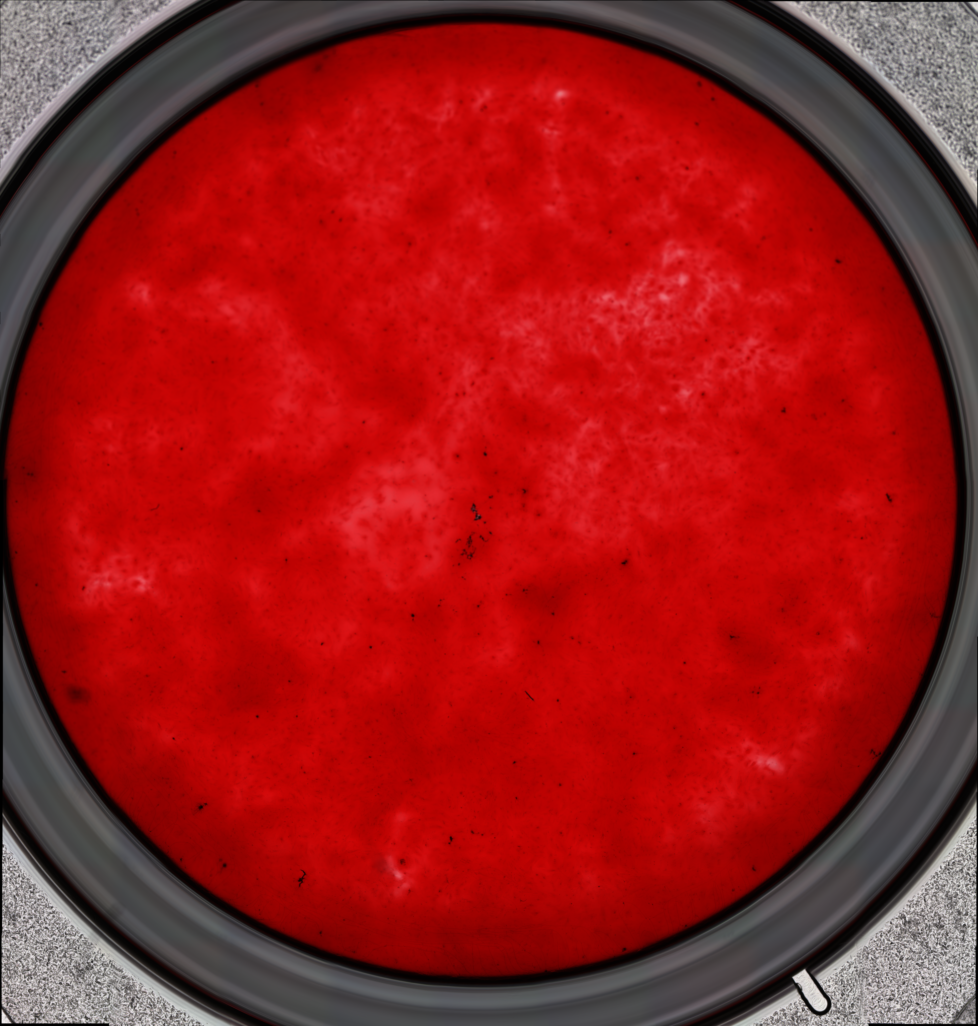

Supplement: Supplementary file 4 — Source data Fig. 3 [file 44318_2024_143_MOESM4_ESM.zip › Figure 3/3F/3F micr image/D170_d21.png]

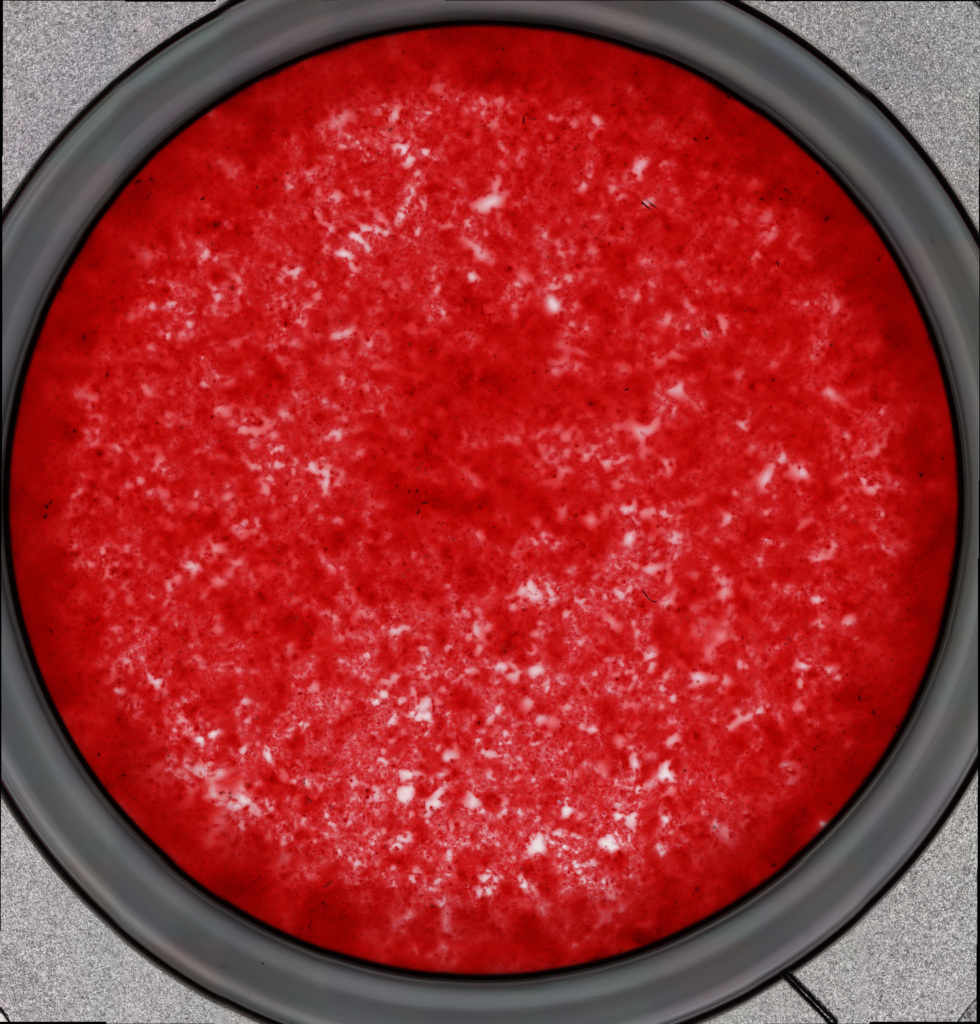

Supplement: Supplementary file 4 — Source data Fig. 3 [file 44318_2024_143_MOESM4_ESM.zip › Figure 3/3F/3F micr image/D239_d21.png]

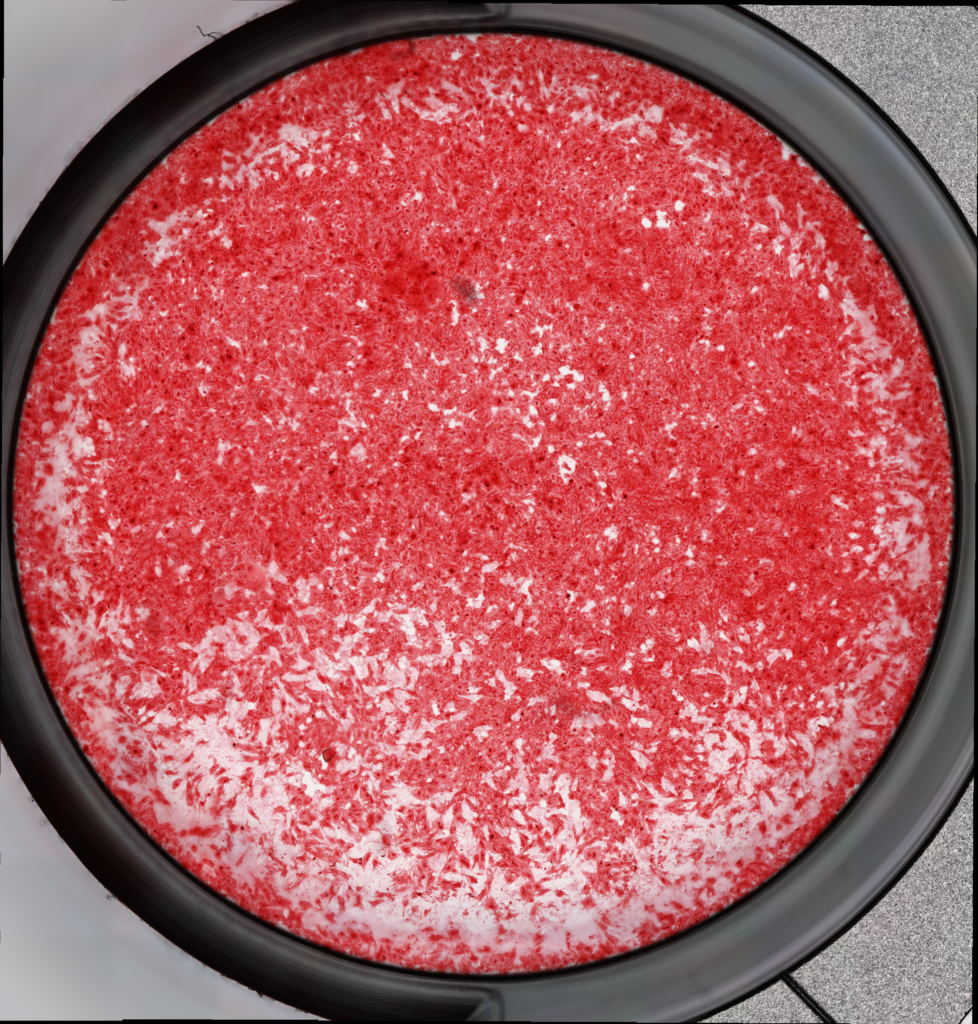

Supplement: Supplementary file 4 — Source data Fig. 3 [file 44318_2024_143_MOESM4_ESM.zip › Figure 3/3F/3F micr image/HUK9_d21.png]

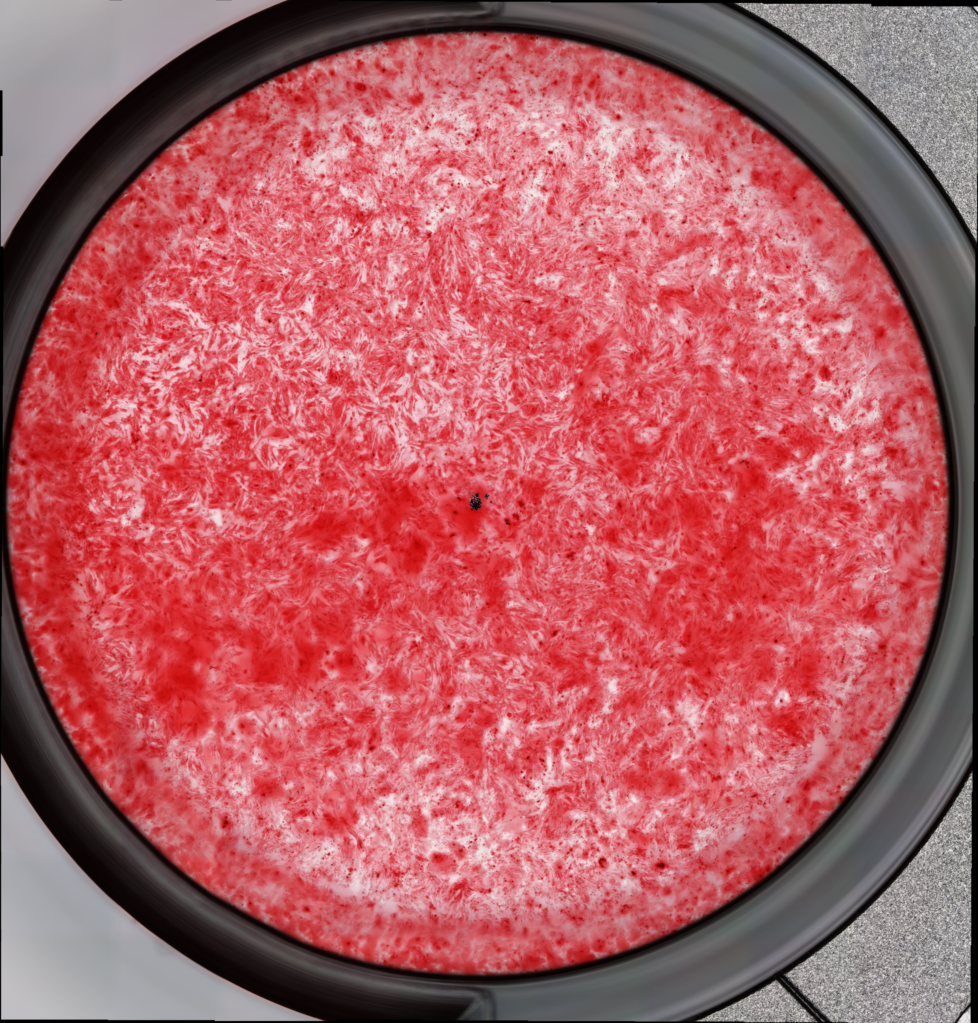

Supplement: Supplementary file 4 — Source data Fig. 3 [file 44318_2024_143_MOESM4_ESM.zip › Figure 3/3F/3F micr image/HUK7_d17.png]

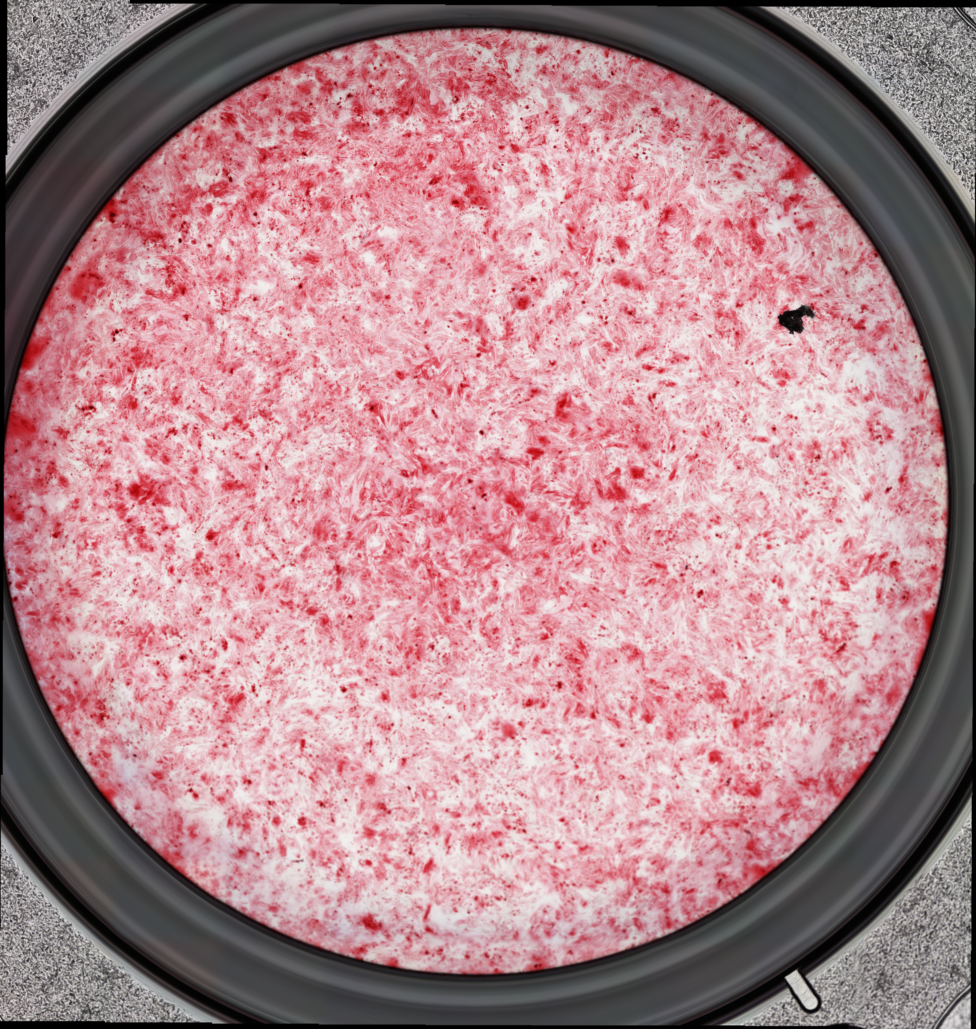

Supplement: Supplementary file 4 — Source data Fig. 3 [file 44318_2024_143_MOESM4_ESM.zip › Figure 3/3F/3F micr image/HUK7_d14.png]

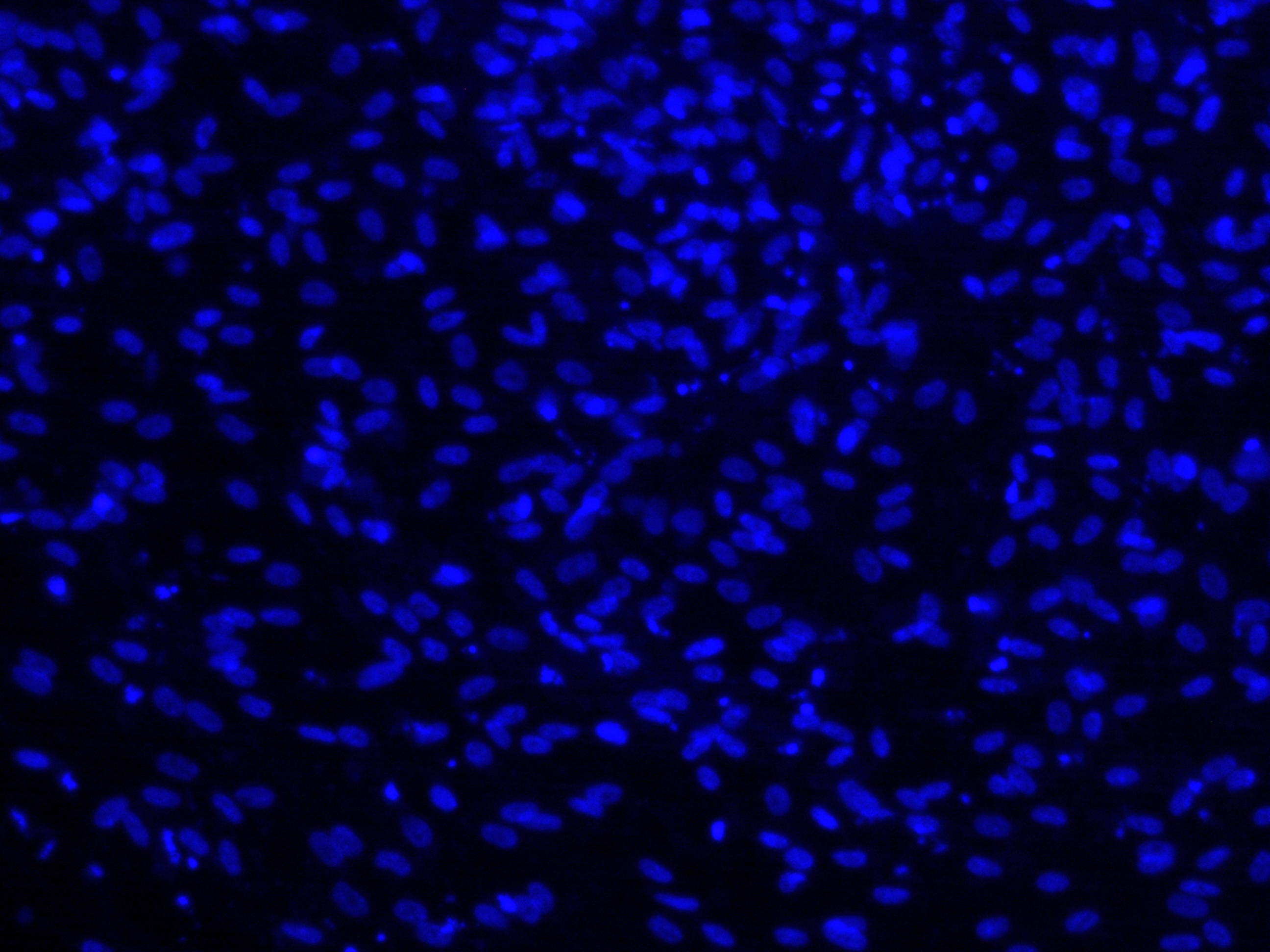

Supplement: Supplementary file 4 — Source data Fig. 3 [file 44318_2024_143_MOESM4_ESM.zip › Figure 3/3G/3G osteoimages/huk16 dapi L1.tif]

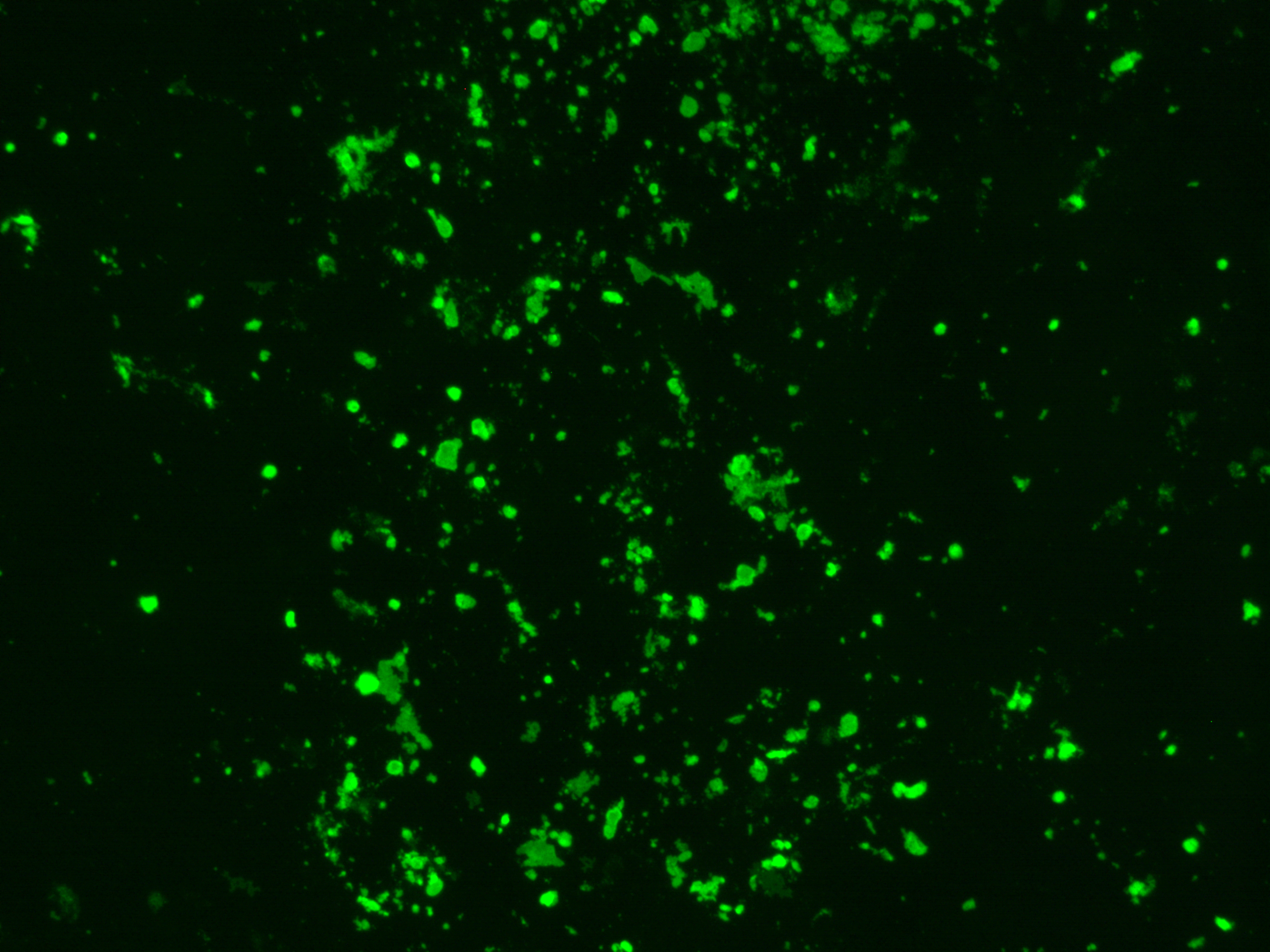

Supplement: Supplementary file 4 — Source data Fig. 3 [file 44318_2024_143_MOESM4_ESM.zip › Figure 3/3G/3G osteoimages/huk12 osteo L1.tif]

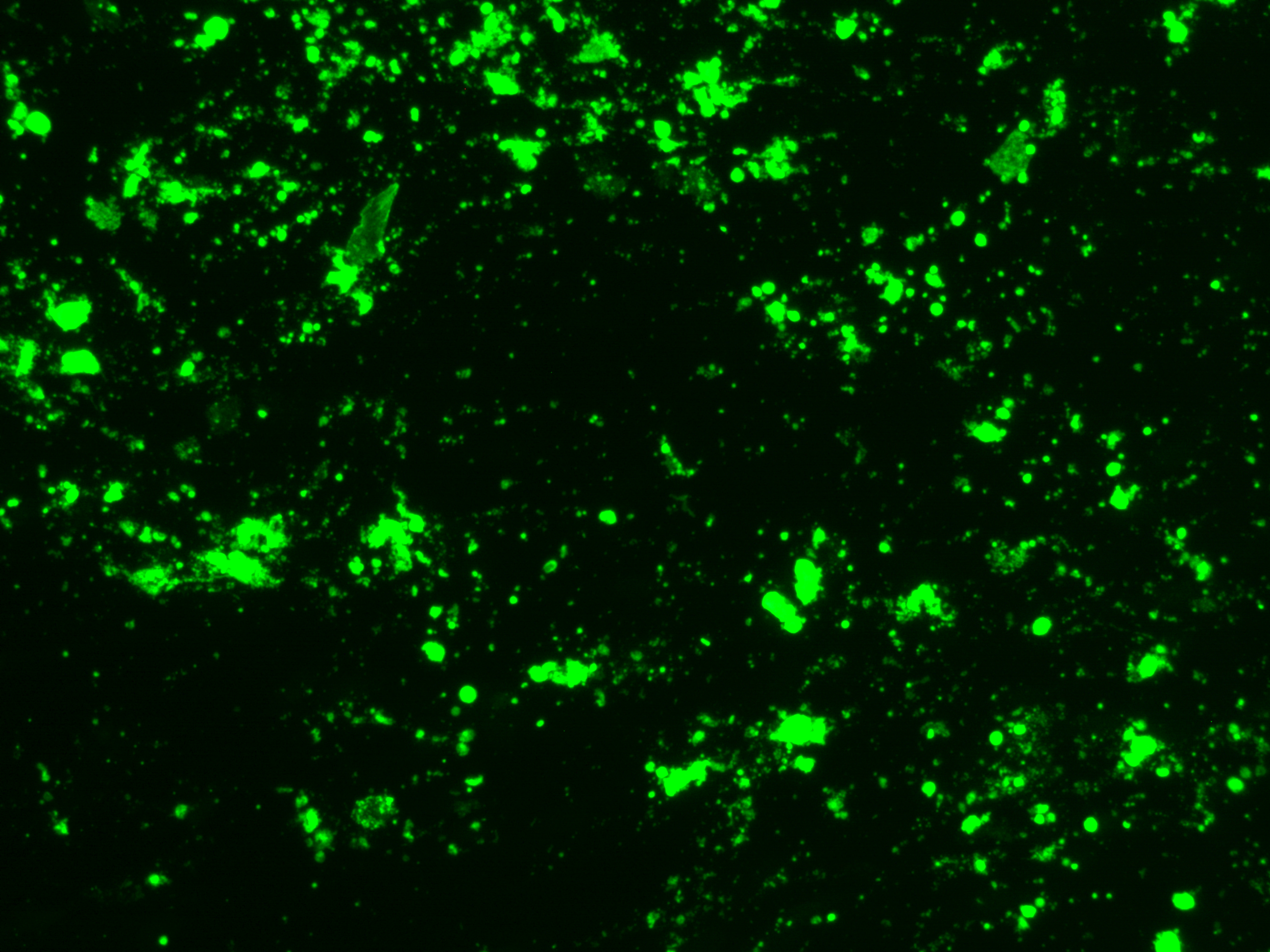

Supplement: Supplementary file 4 — Source data Fig. 3 [file 44318_2024_143_MOESM4_ESM.zip › Figure 3/3G/3G osteoimages/huk9 osteo L1.tif]

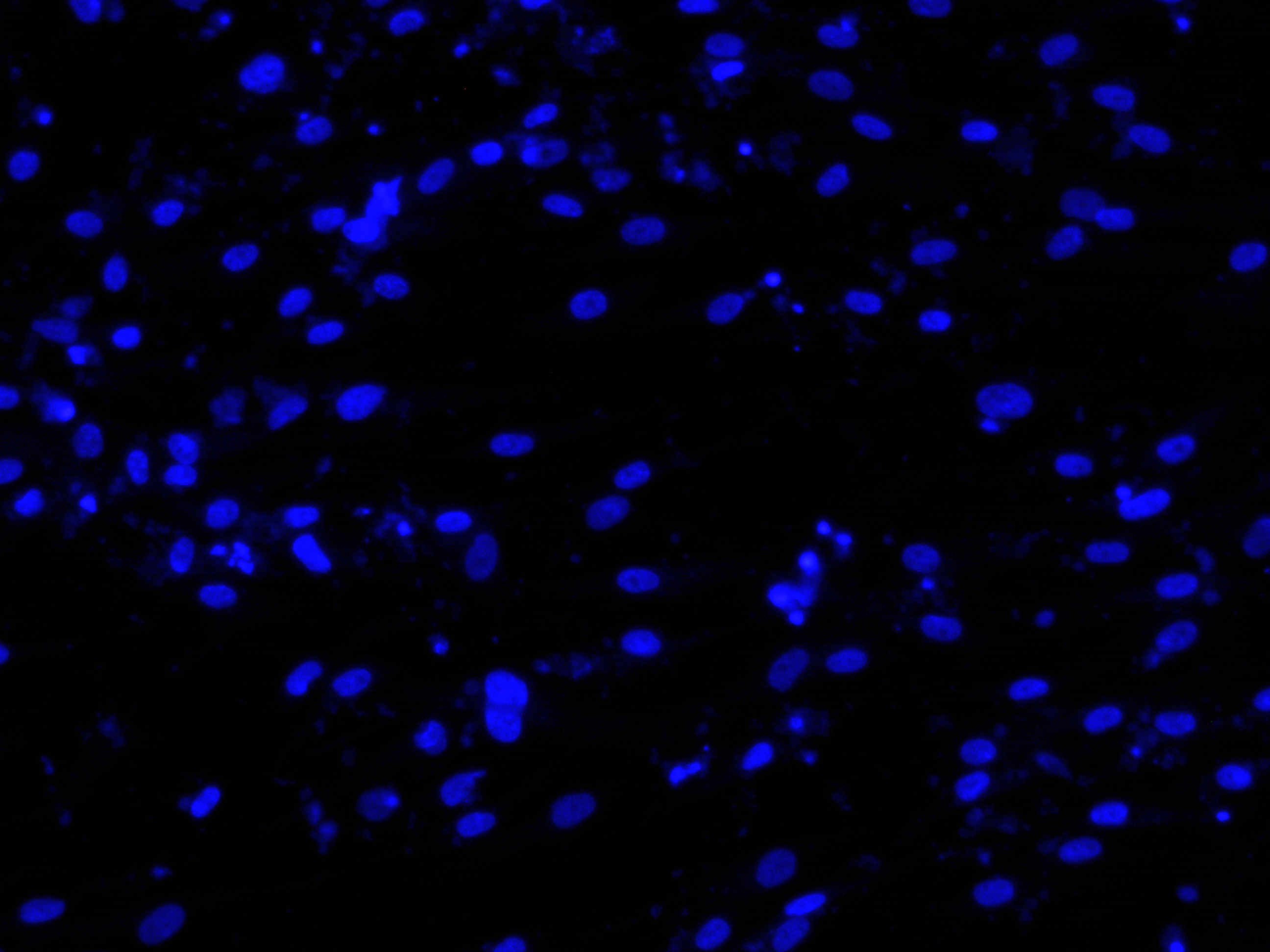

Supplement: Supplementary file 4 — Source data Fig. 3 [file 44318_2024_143_MOESM4_ESM.zip › Figure 3/3G/3G osteoimages/huk9 dapi L1.tif]

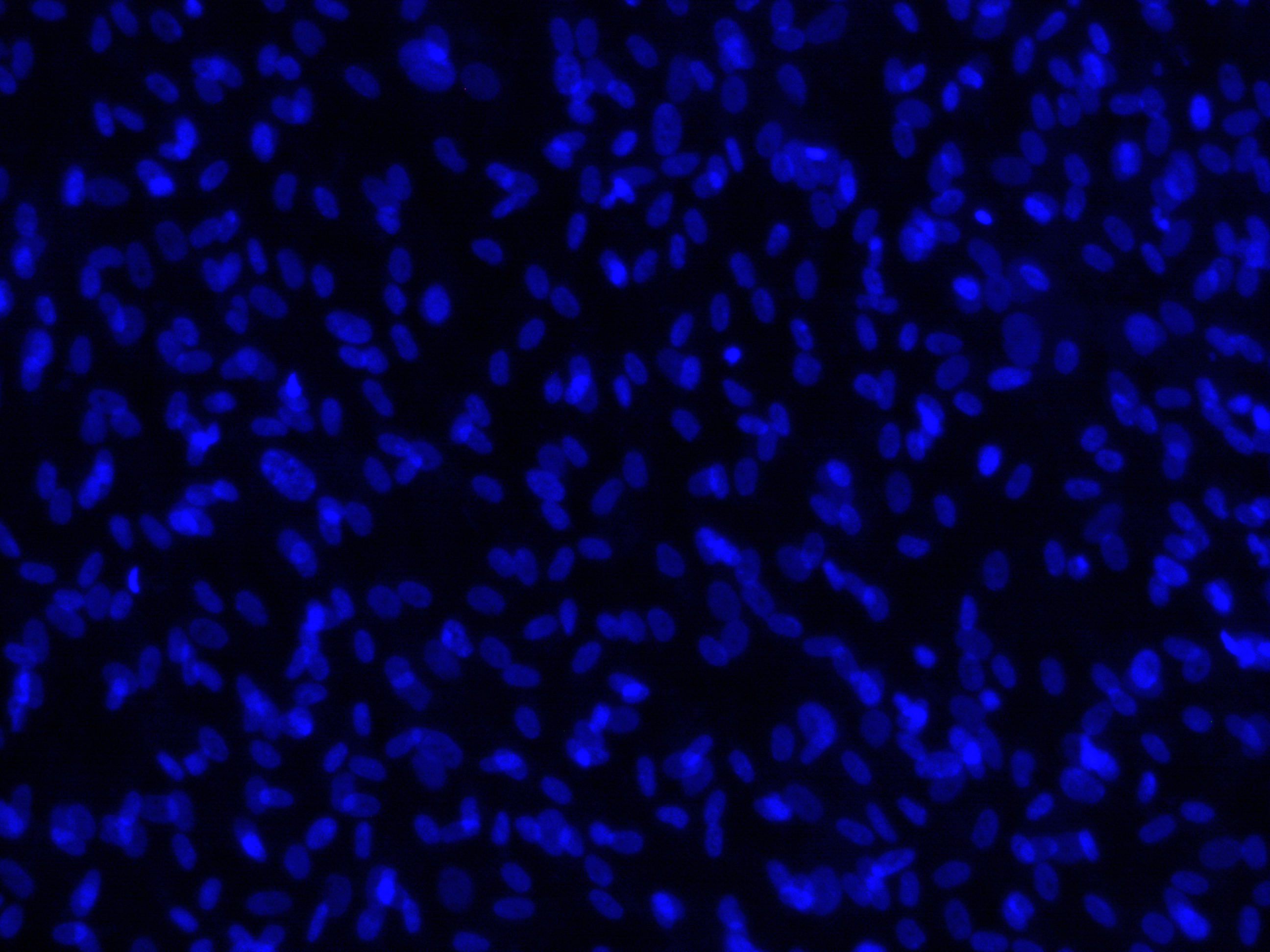

Supplement: Supplementary file 4 — Source data Fig. 3 [file 44318_2024_143_MOESM4_ESM.zip › Figure 3/3G/3G osteoimages/huk16 dapi RFP.tif]

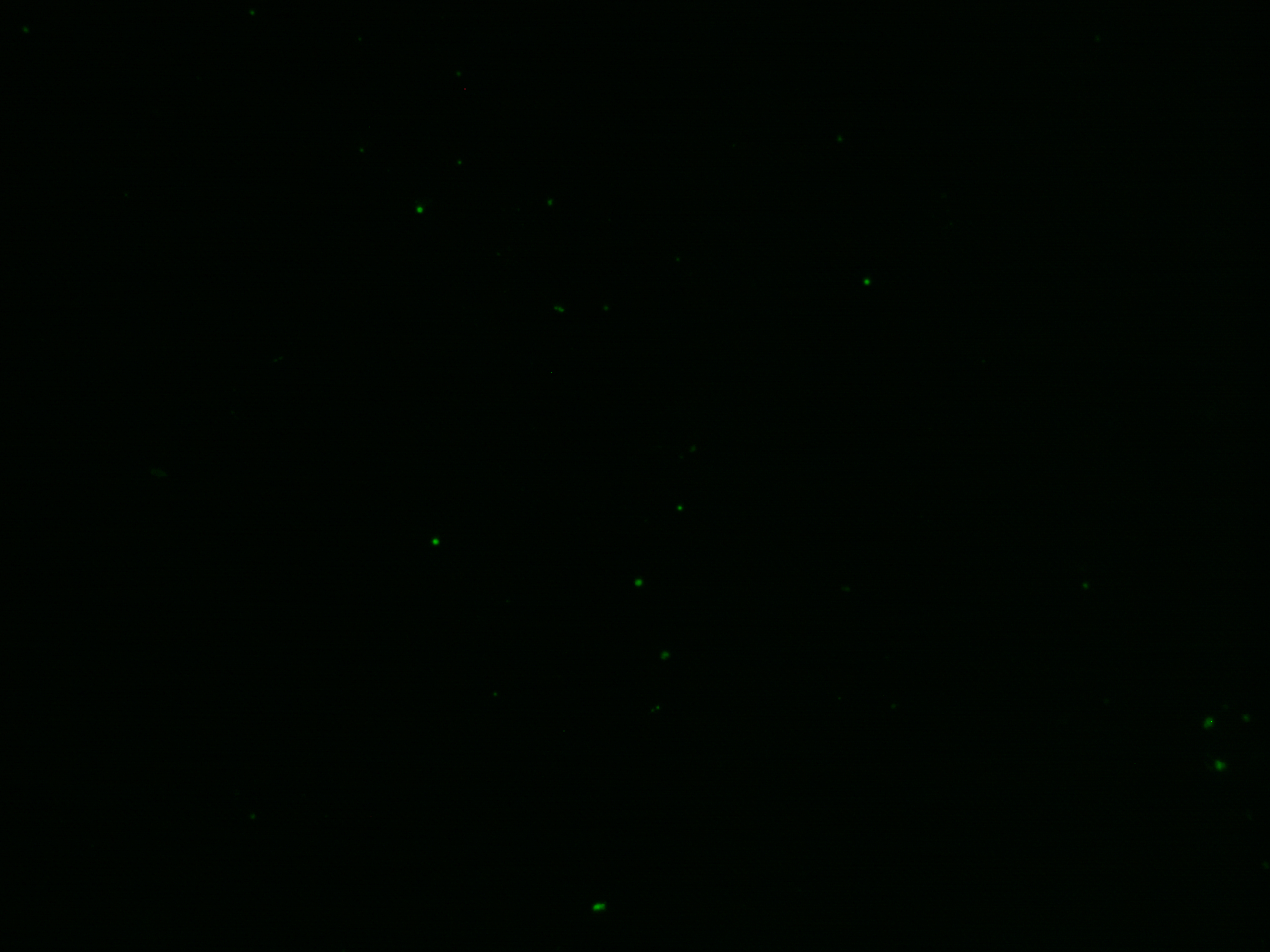

Supplement: Supplementary file 4 — Source data Fig. 3 [file 44318_2024_143_MOESM4_ESM.zip › Figure 3/3G/3G osteoimages/huk12 osteo RFP.tif]

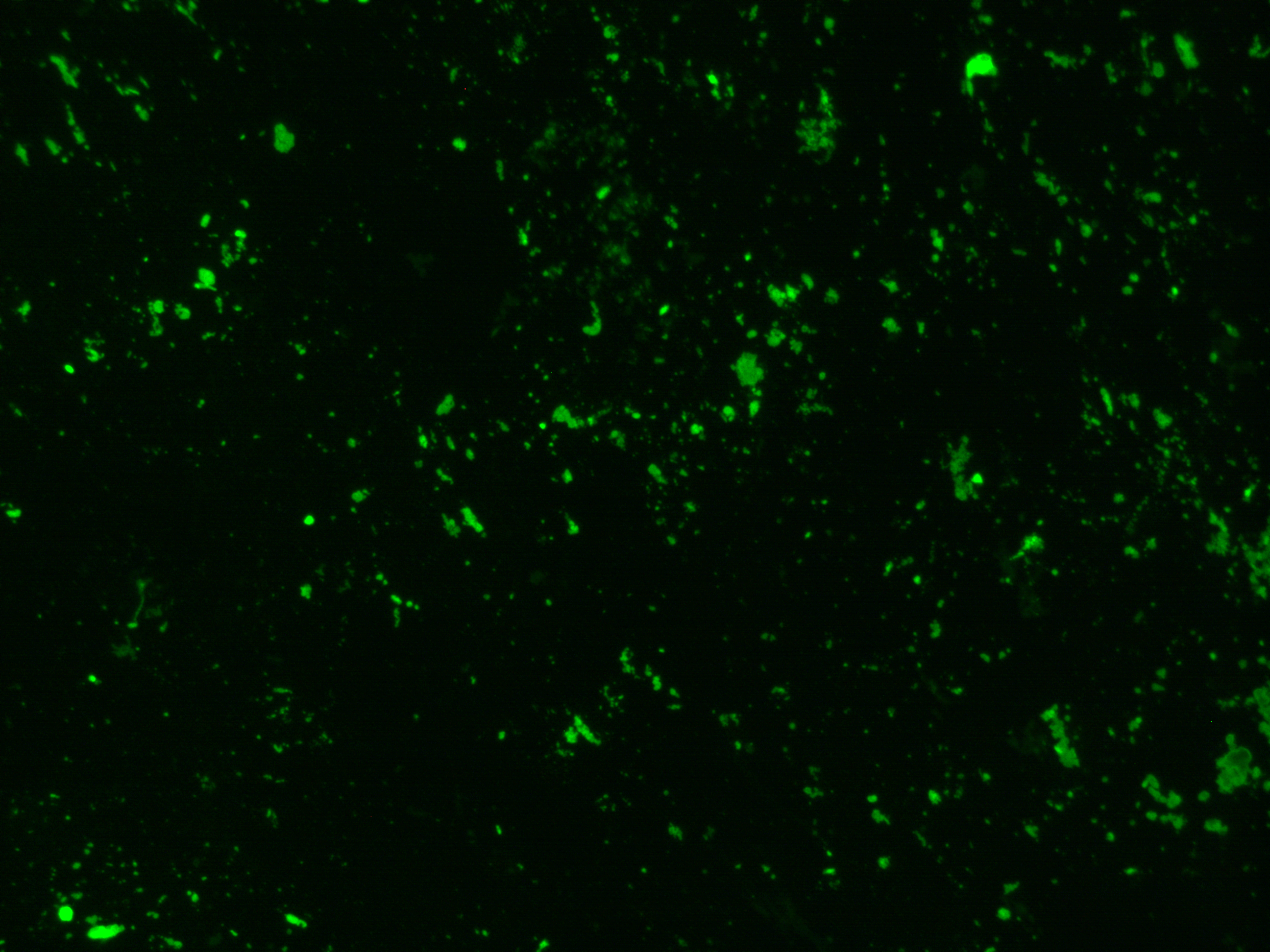

Supplement: Supplementary file 4 — Source data Fig. 3 [file 44318_2024_143_MOESM4_ESM.zip › Figure 3/3G/3G osteoimages/huk16 osteo RFP.tif]

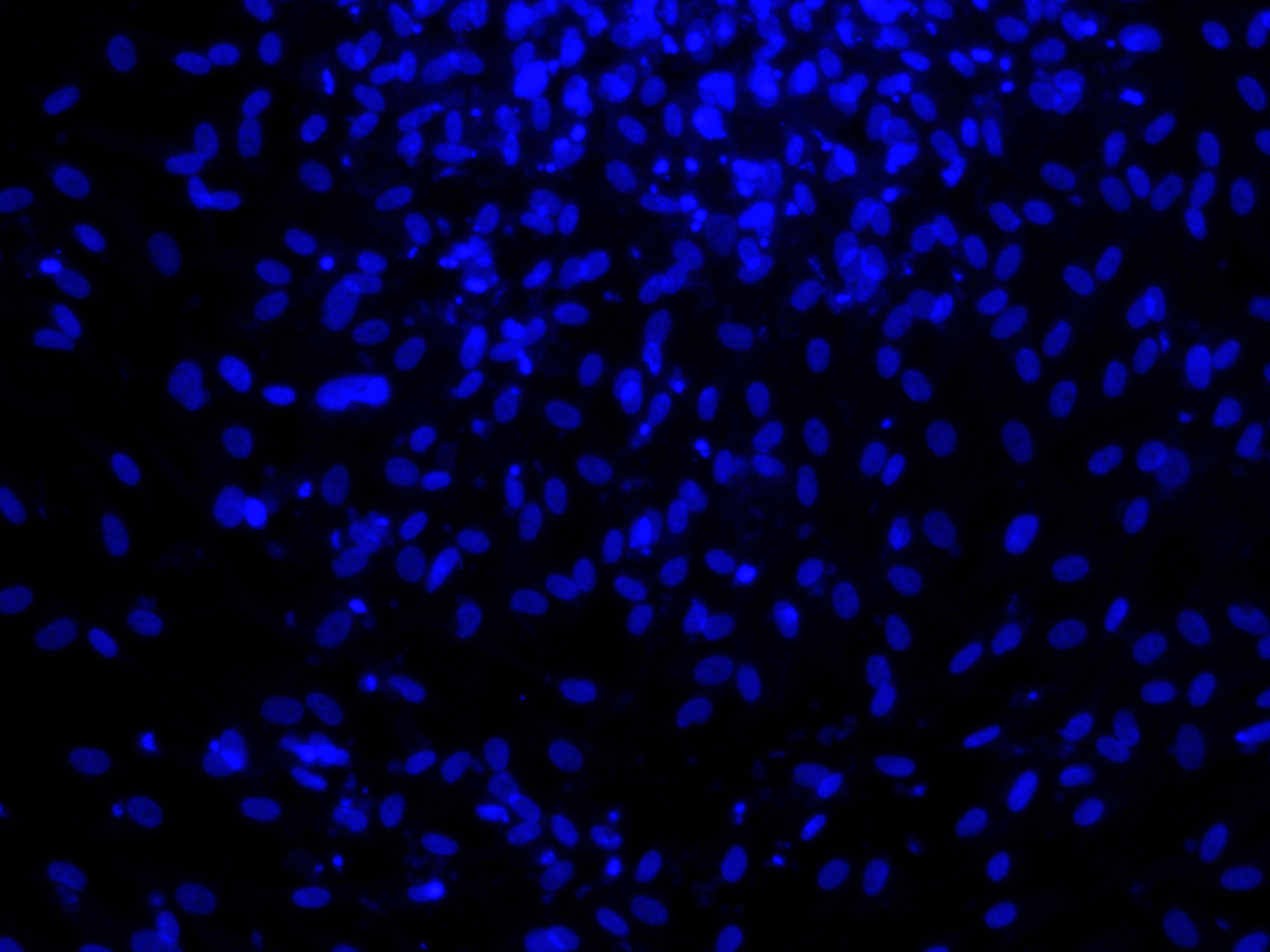

Supplement: Supplementary file 4 — Source data Fig. 3 [file 44318_2024_143_MOESM4_ESM.zip › Figure 3/3G/3G osteoimages/huk12 dapi L1.tif]

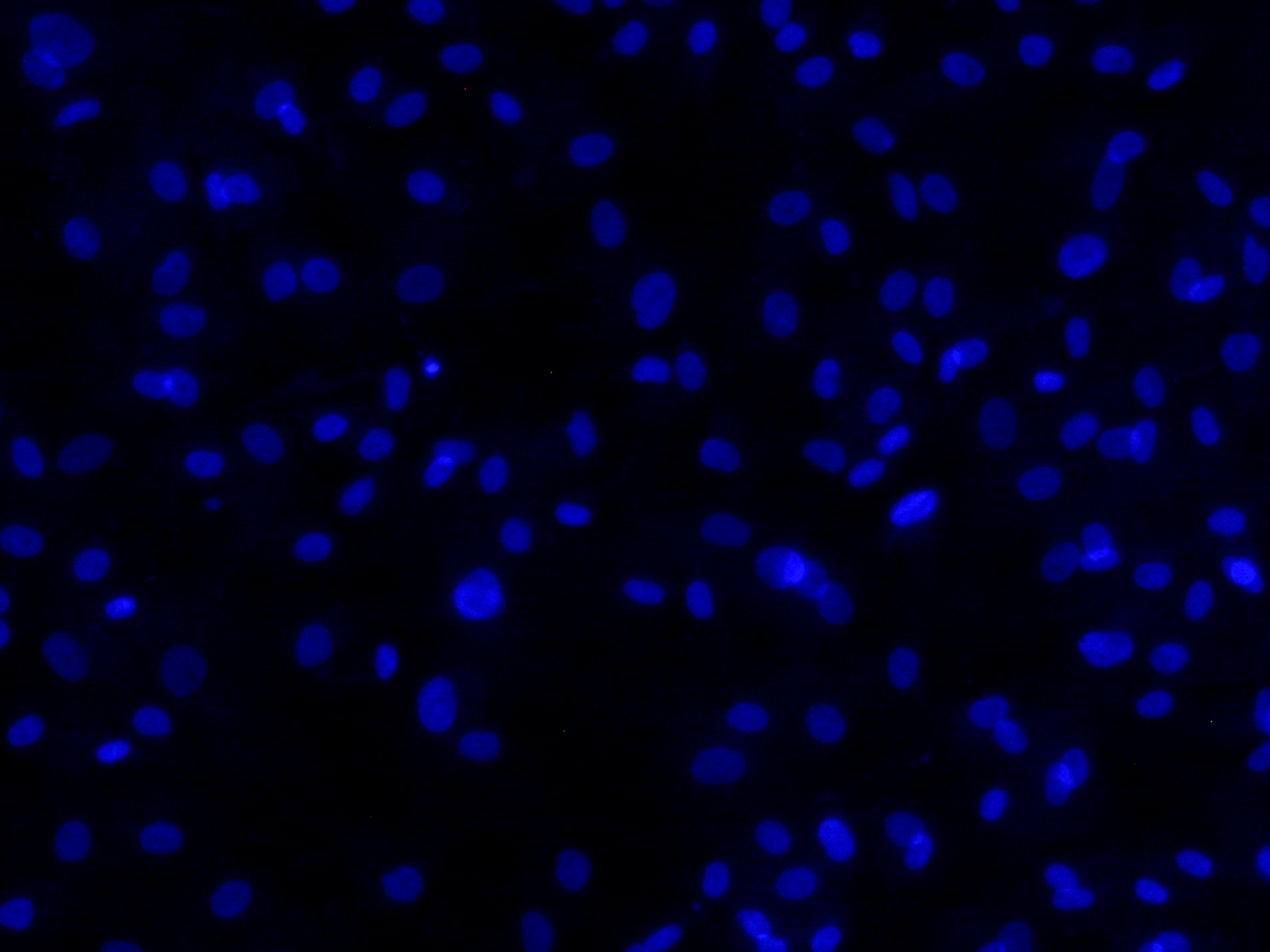

Supplement: Supplementary file 4 — Source data Fig. 3 [file 44318_2024_143_MOESM4_ESM.zip › Figure 3/3G/3G osteoimages/huk9 dapi RFP.tif]

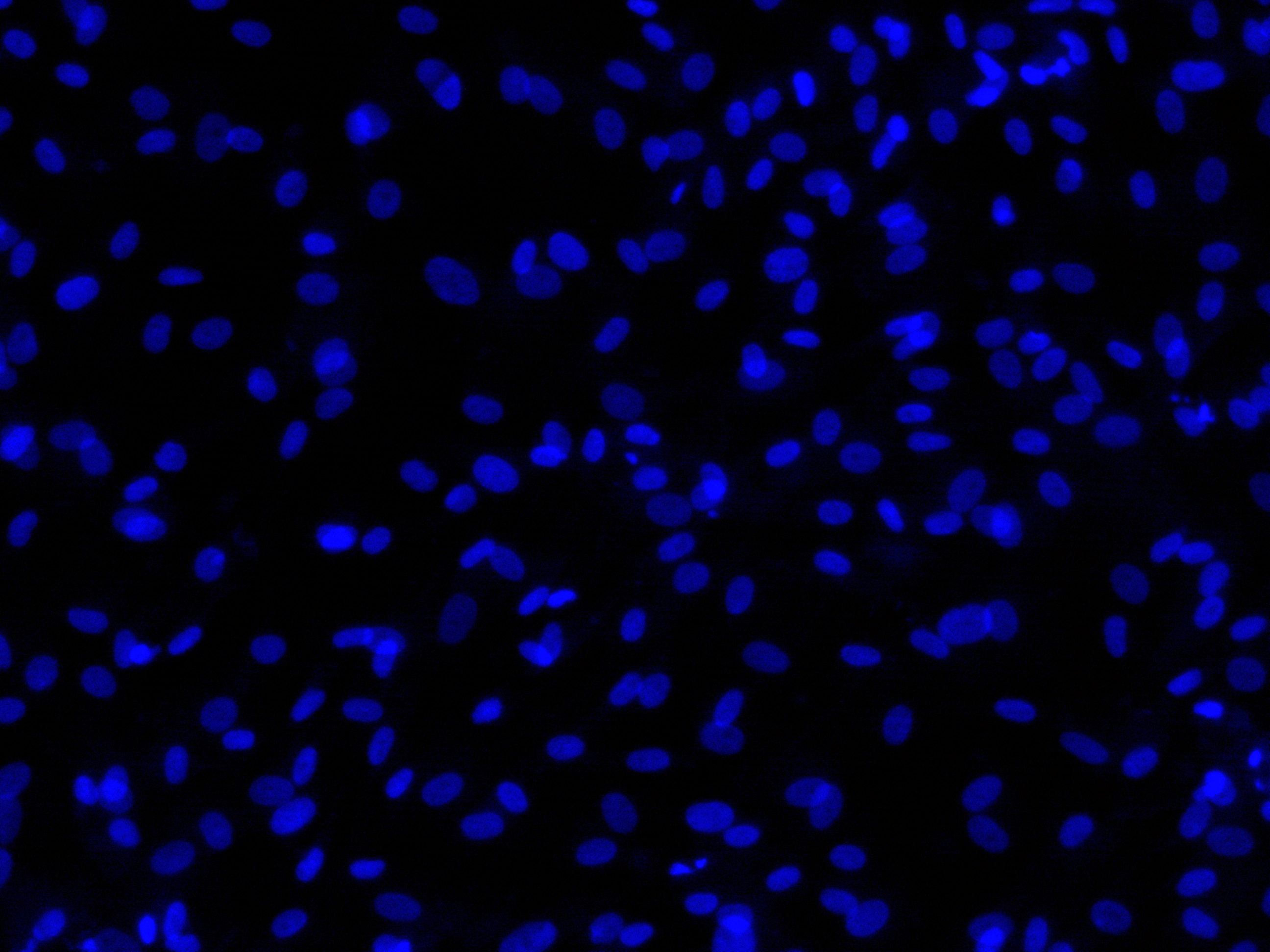

Supplement: Supplementary file 4 — Source data Fig. 3 [file 44318_2024_143_MOESM4_ESM.zip › Figure 3/3G/3G osteoimages/huk12 dapi RFP.tif]

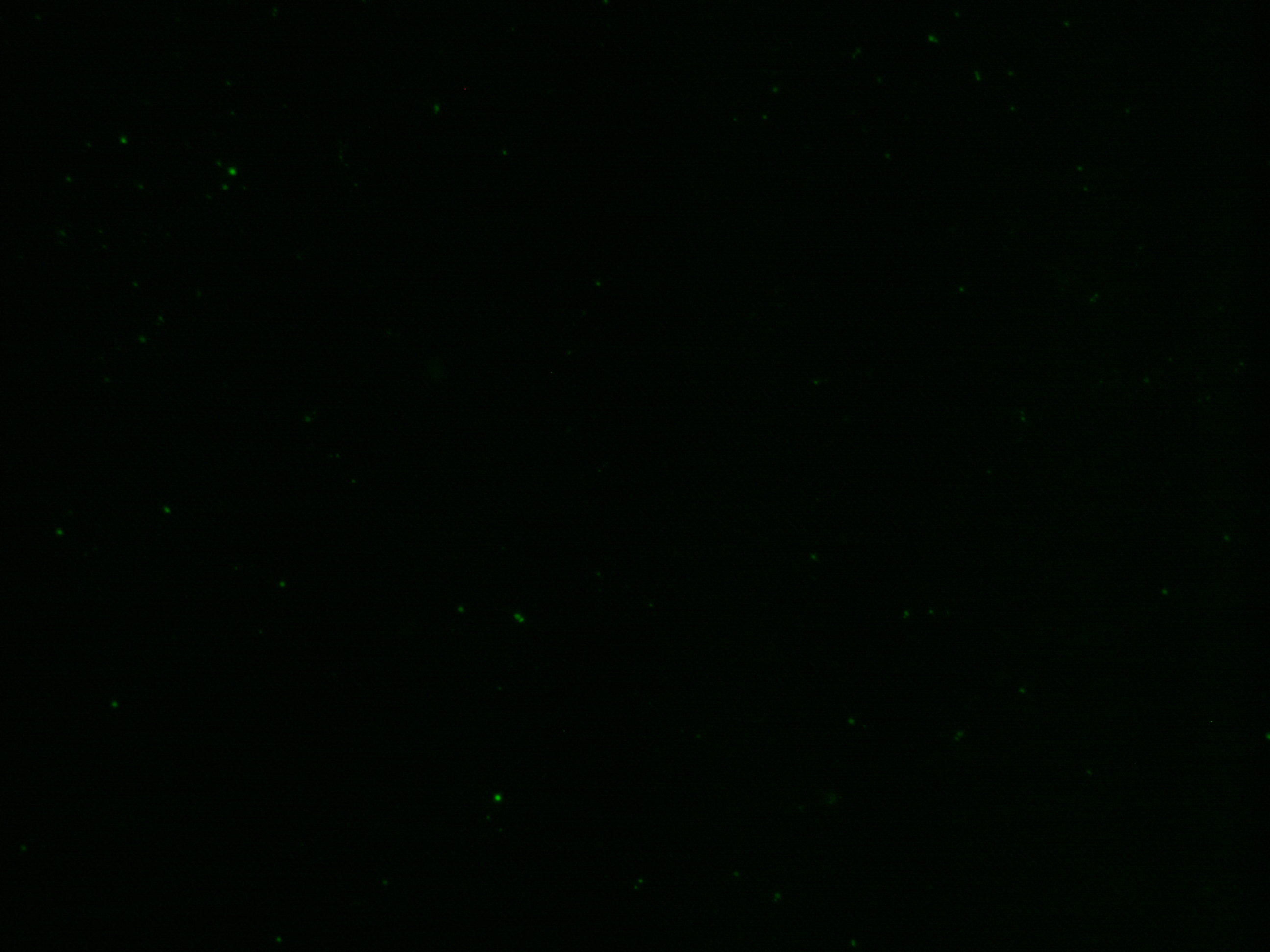

Supplement: Supplementary file 4 — Source data Fig. 3 [file 44318_2024_143_MOESM4_ESM.zip › Figure 3/3G/3G osteoimages/huk9 osteo RFP.tif]

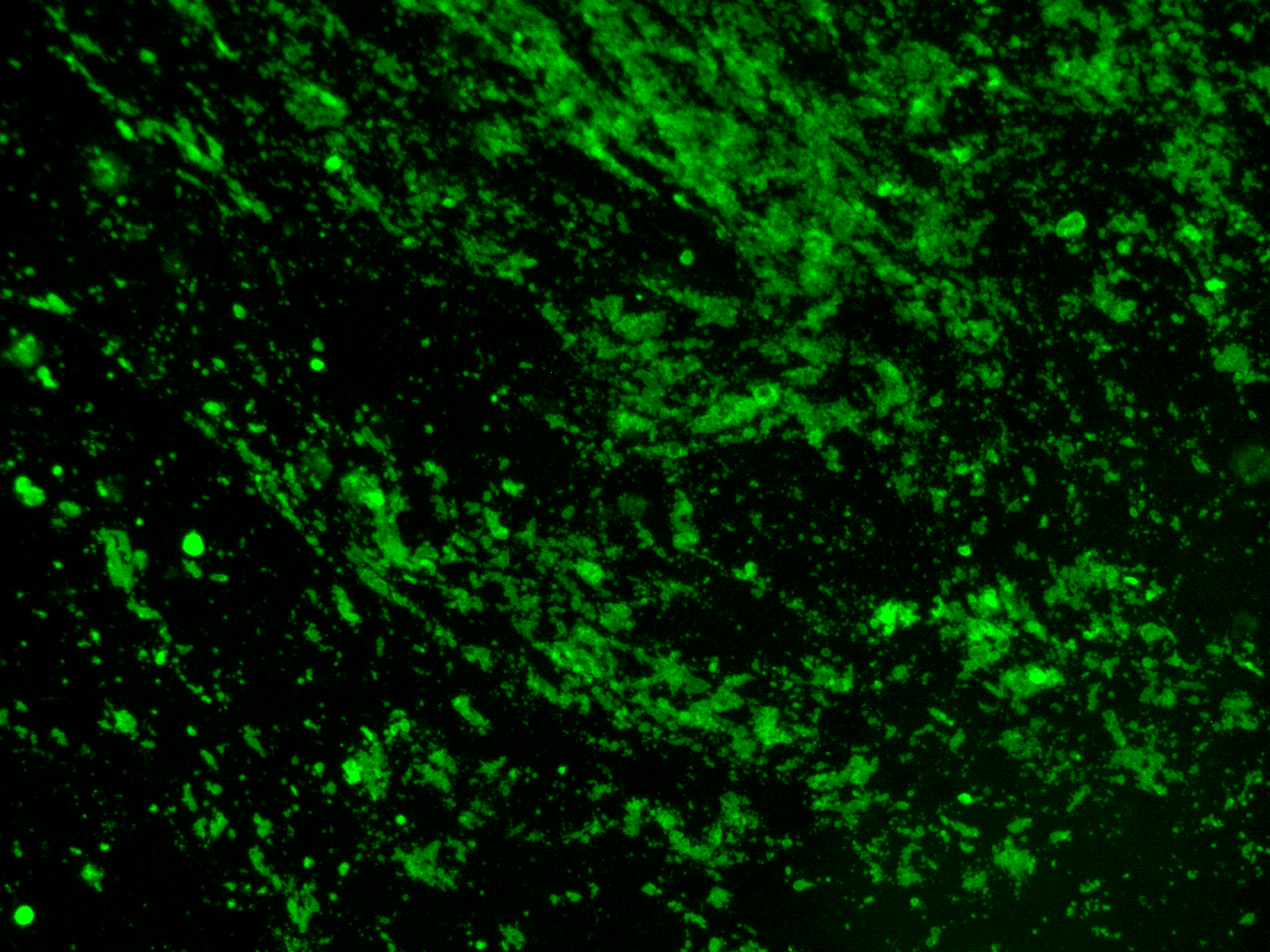

Supplement: Supplementary file 4 — Source data Fig. 3 [file 44318_2024_143_MOESM4_ESM.zip › Figure 3/3G/3G osteoimages/huk16 osteo L1.tif]

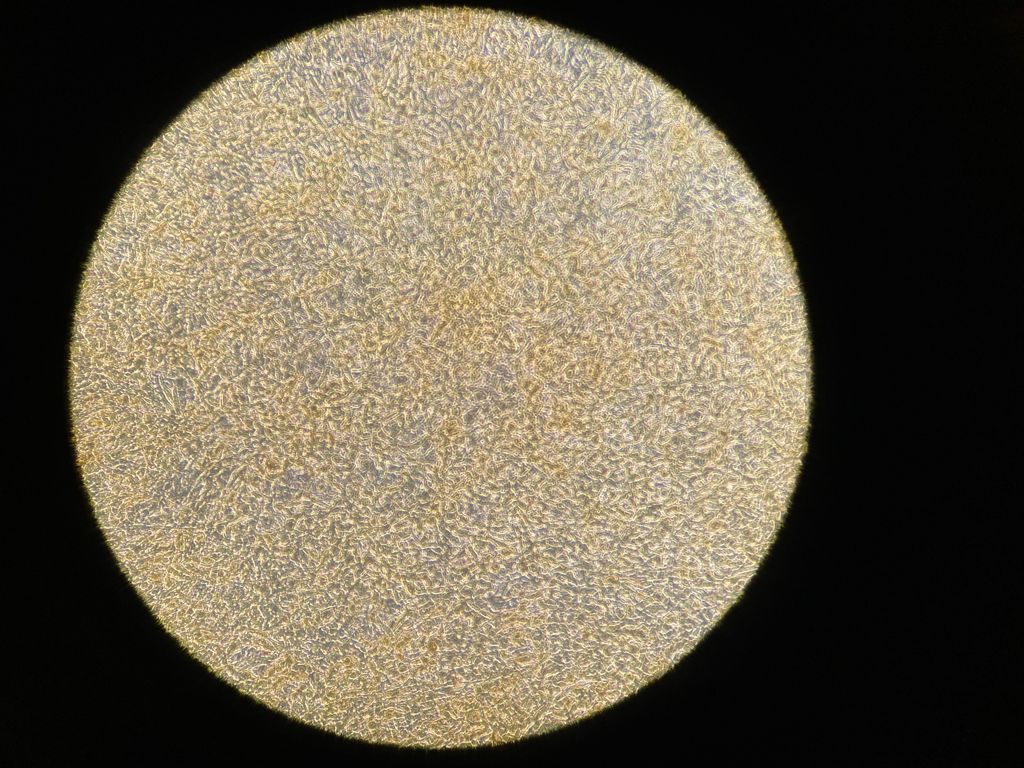

Supplement: Supplementary file 4 — Source data Fig. 3 [file 44318_2024_143_MOESM4_ESM.zip › Figure 3/3G/3G Alizarin images/HUK9 RFP.JPG]

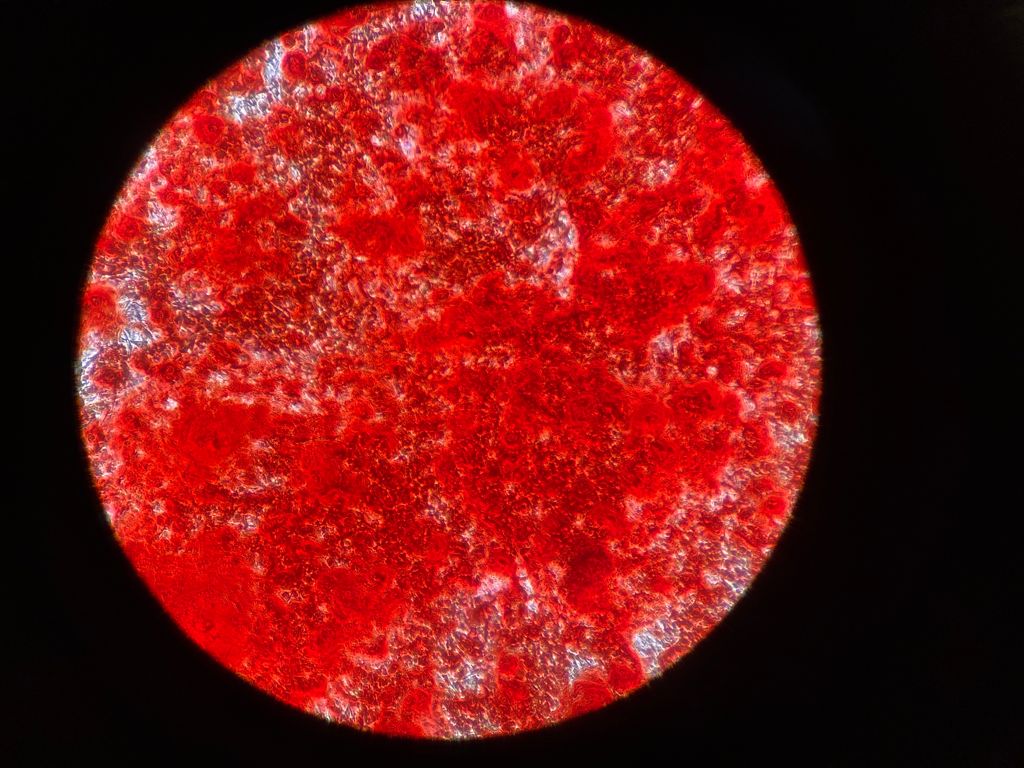

Supplement: Supplementary file 4 — Source data Fig. 3 [file 44318_2024_143_MOESM4_ESM.zip › Figure 3/3G/3G Alizarin images/HUK9 L1.JPG]

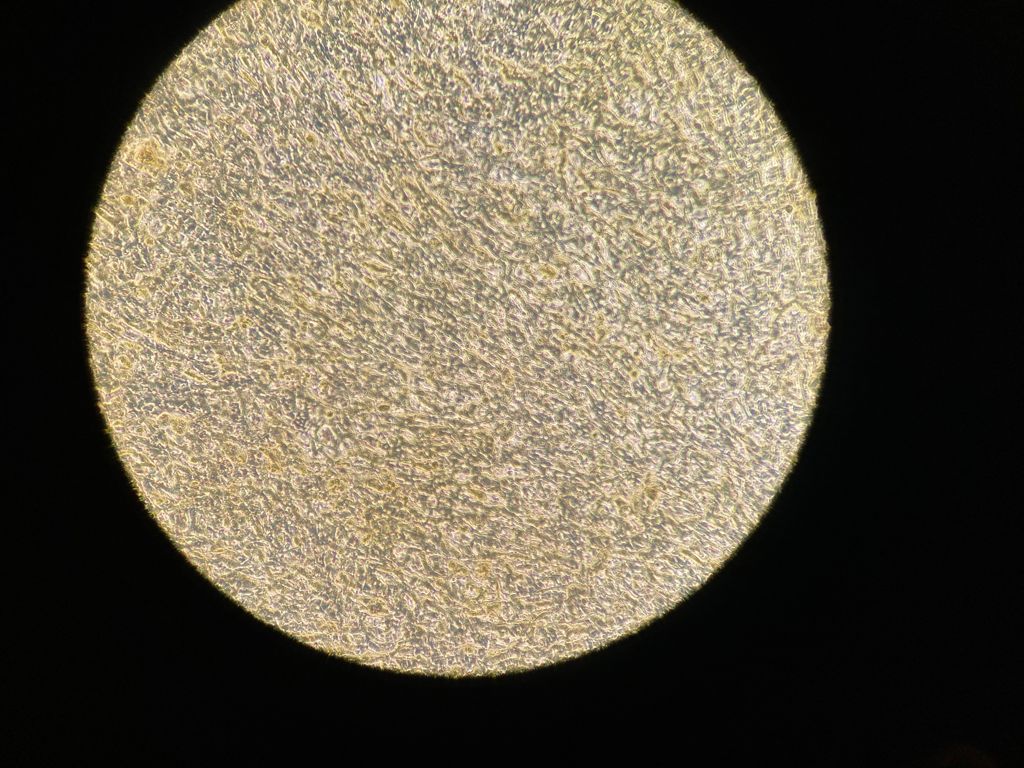

Supplement: Supplementary file 4 — Source data Fig. 3 [file 44318_2024_143_MOESM4_ESM.zip › Figure 3/3G/3G Alizarin images/HUK12 RFP.JPG]

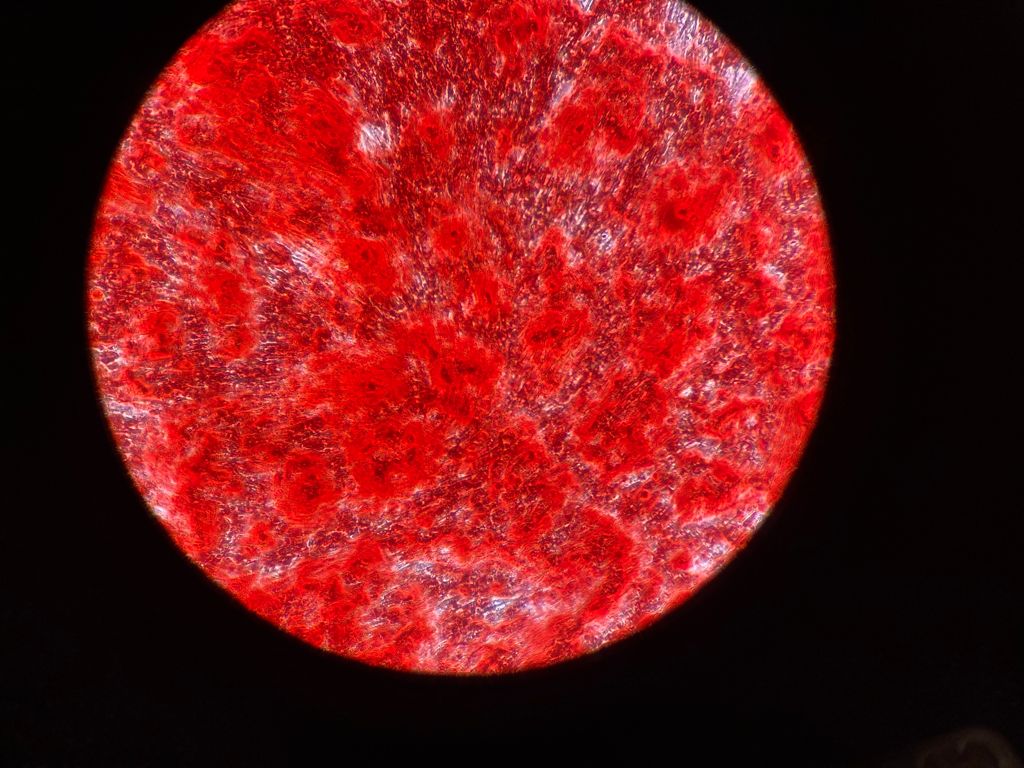

Supplement: Supplementary file 4 — Source data Fig. 3 [file 44318_2024_143_MOESM4_ESM.zip › Figure 3/3G/3G Alizarin images/HUK12 L1.JPG]

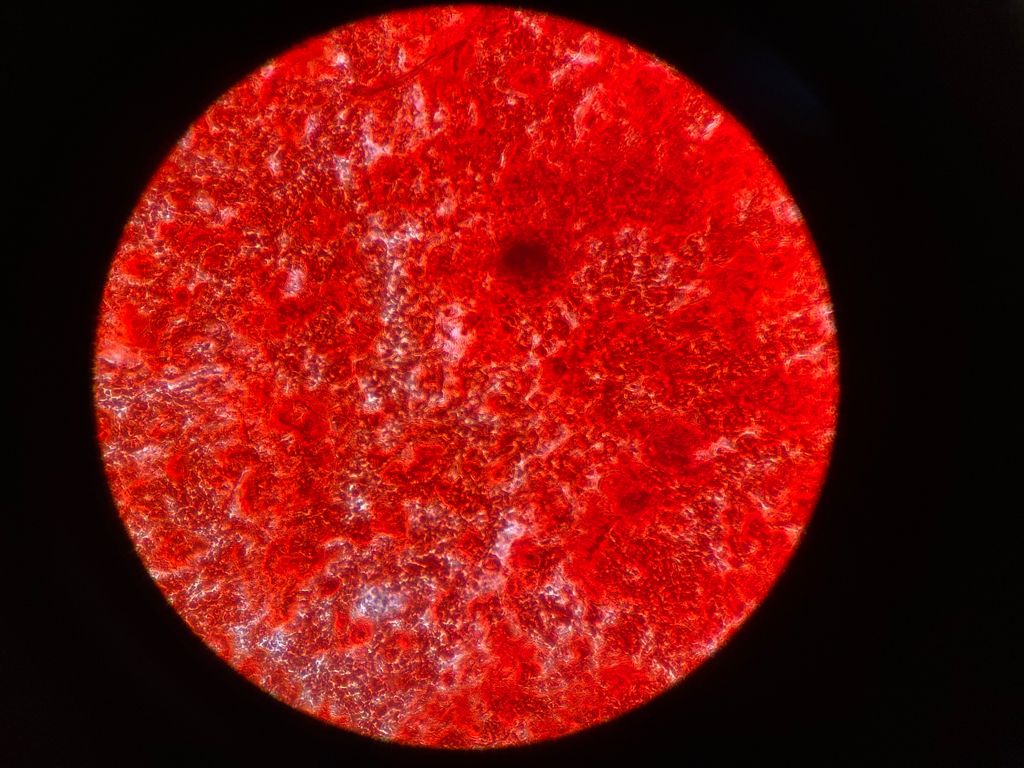

Supplement: Supplementary file 4 — Source data Fig. 3 [file 44318_2024_143_MOESM4_ESM.zip › Figure 3/3G/3G Alizarin images/HUK16 L1.JPG]

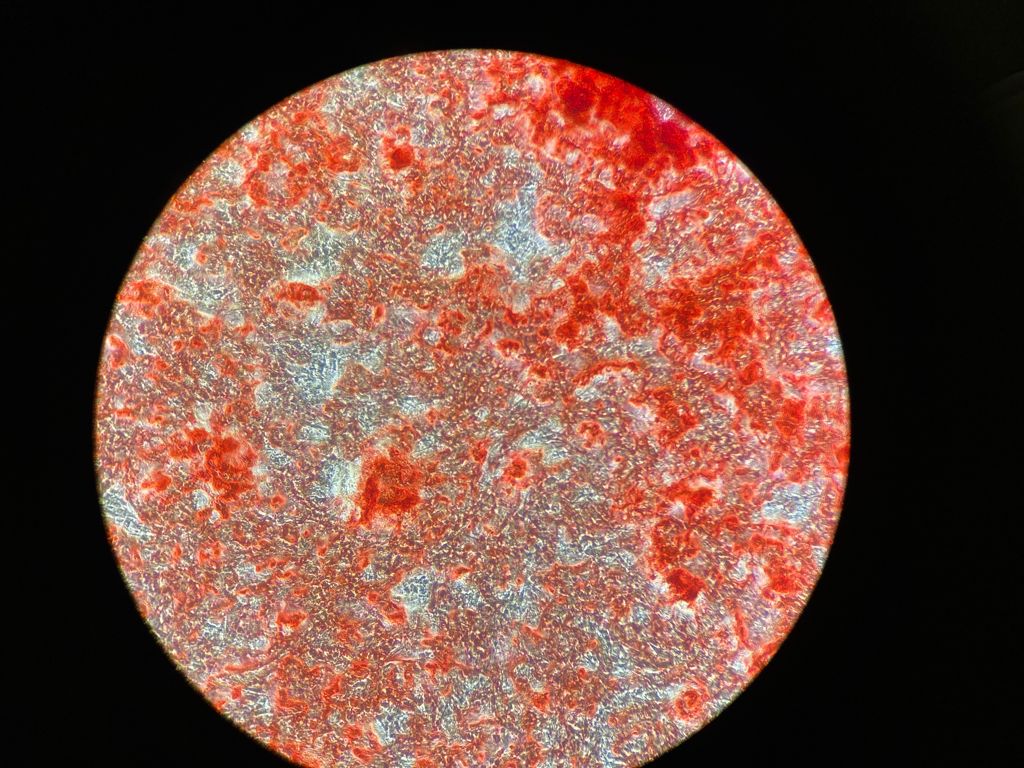

Supplement: Supplementary file 4 — Source data Fig. 3 [file 44318_2024_143_MOESM4_ESM.zip › Figure 3/3G/3G Alizarin images/HUK16 RFP.JPG]

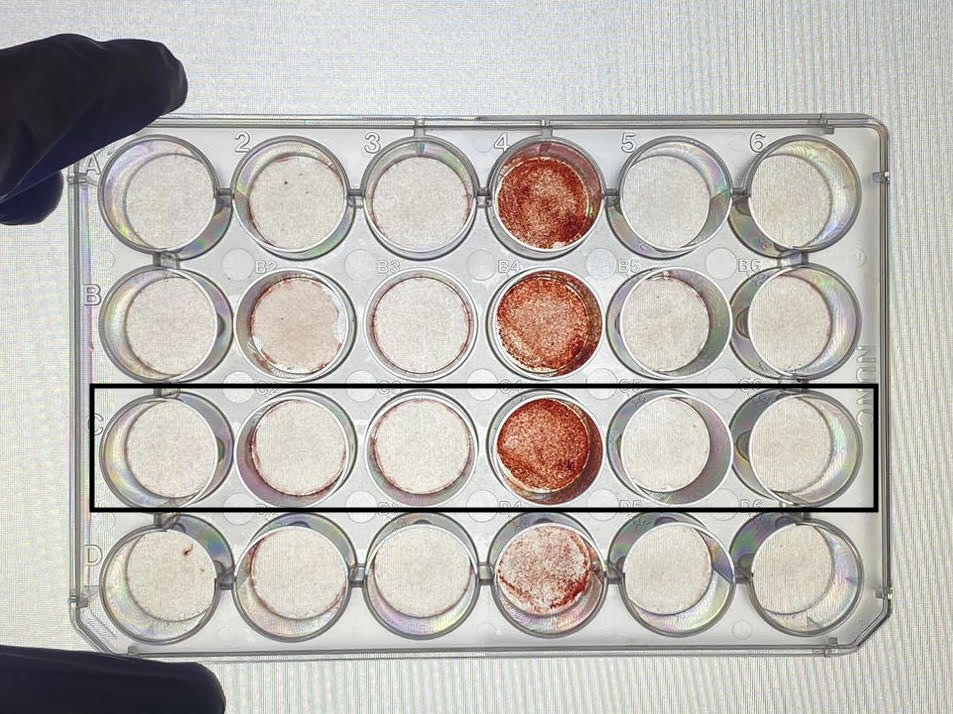

Supplement: Supplementary file 6 — Source data Fig. 5 [file 44318_2024_143_MOESM6_ESM.zip › Figure 5/5F/5F image.jpg]

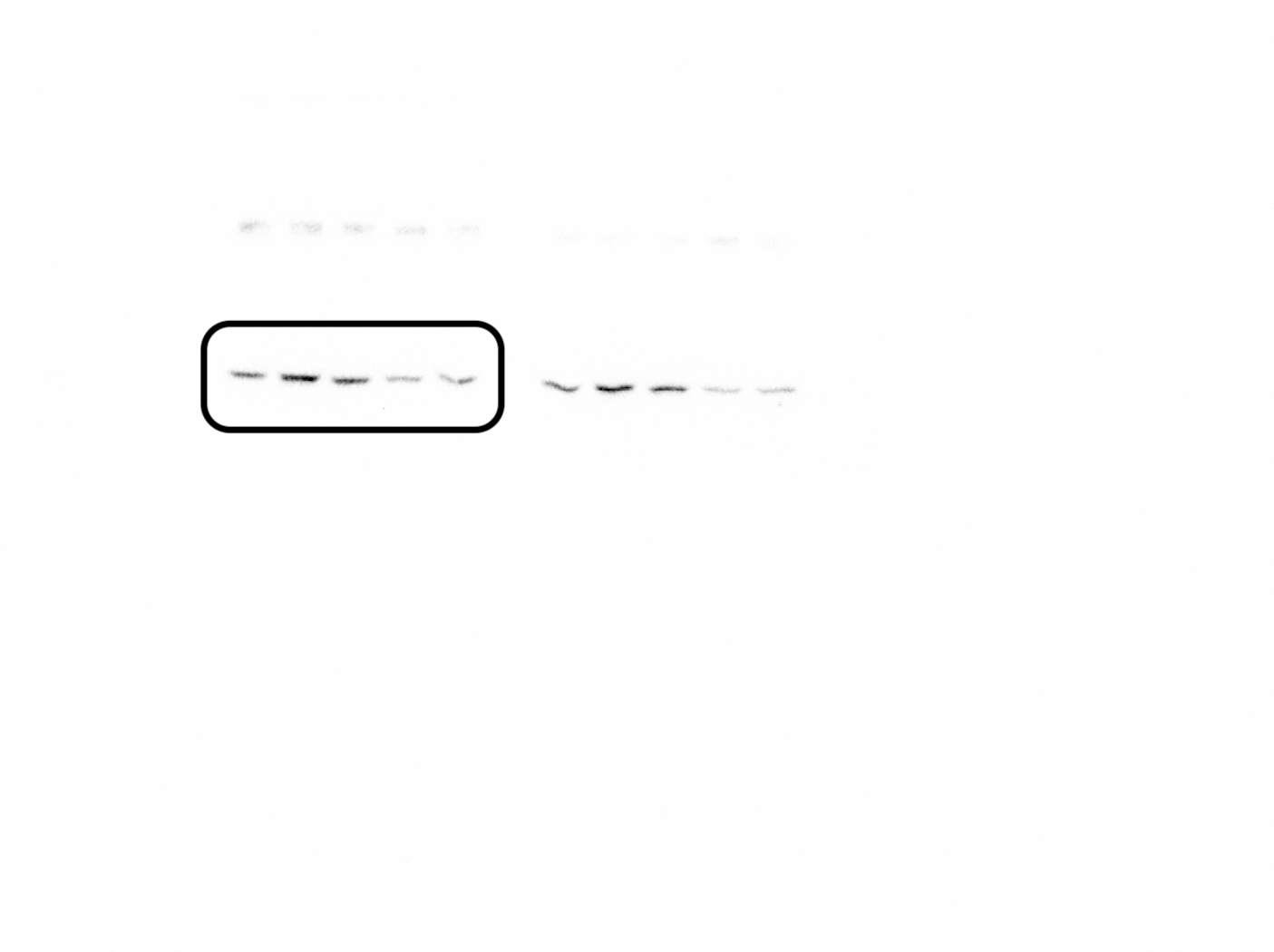

Supplement: Supplementary file 6 — Source data Fig. 5 [file 44318_2024_143_MOESM6_ESM.zip › Figure 5/5C/H3.tif]

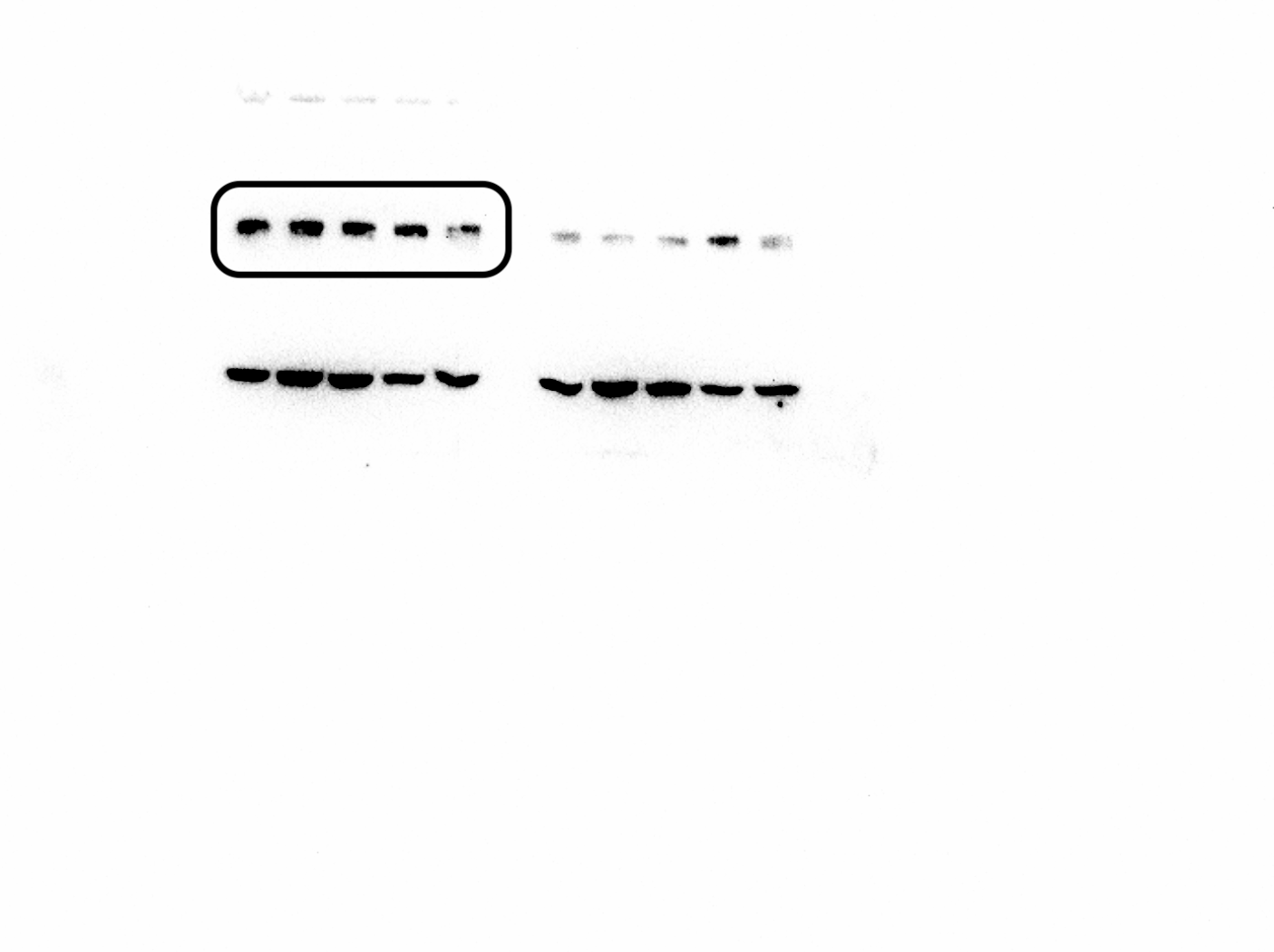

Supplement: Supplementary file 6 — Source data Fig. 5 [file 44318_2024_143_MOESM6_ESM.zip › Figure 5/5C/eIF2alpha.tif]

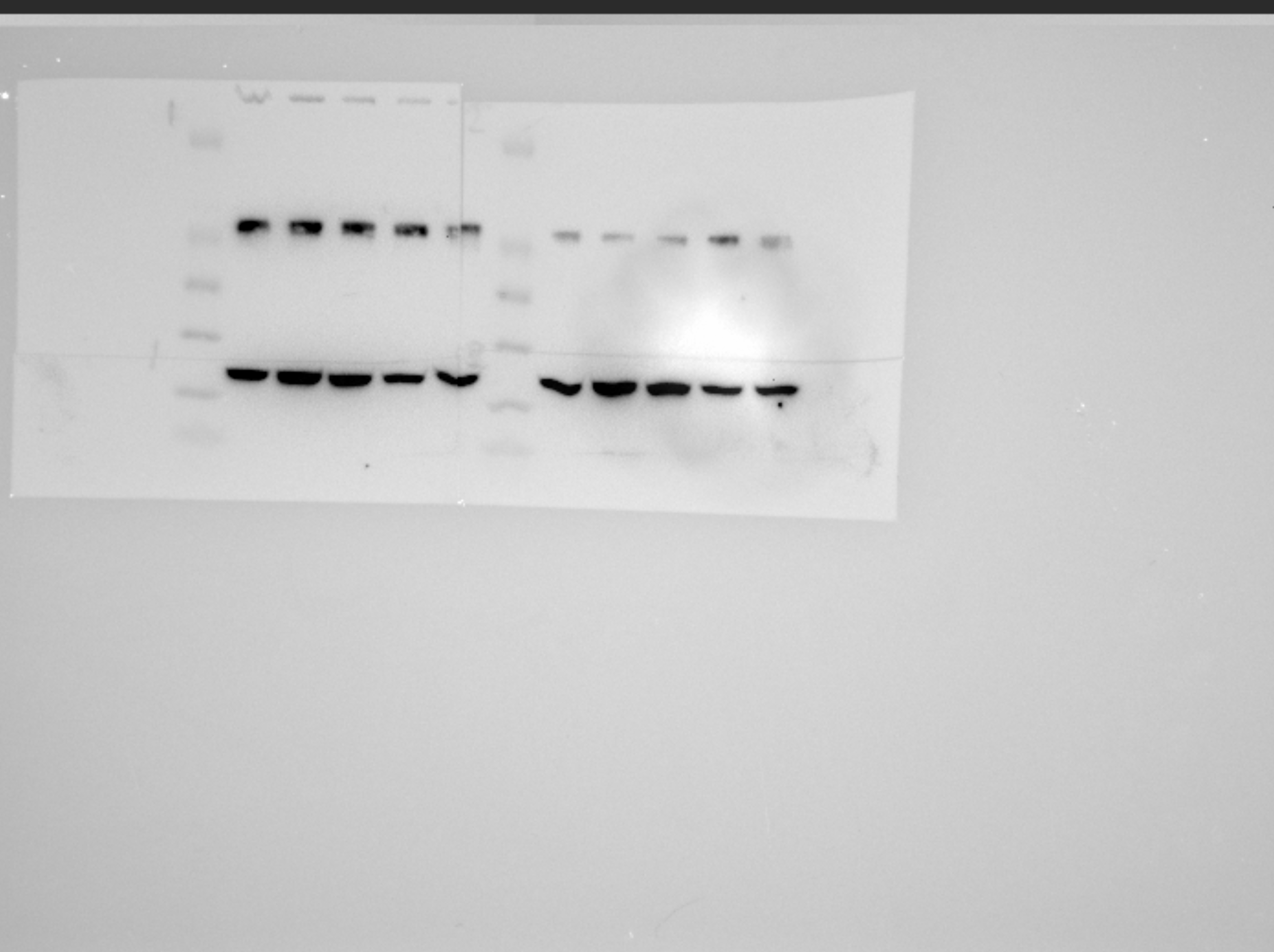

Supplement: Supplementary file 6 — Source data Fig. 5 [file 44318_2024_143_MOESM6_ESM.zip › Figure 5/5C/merge.tif]

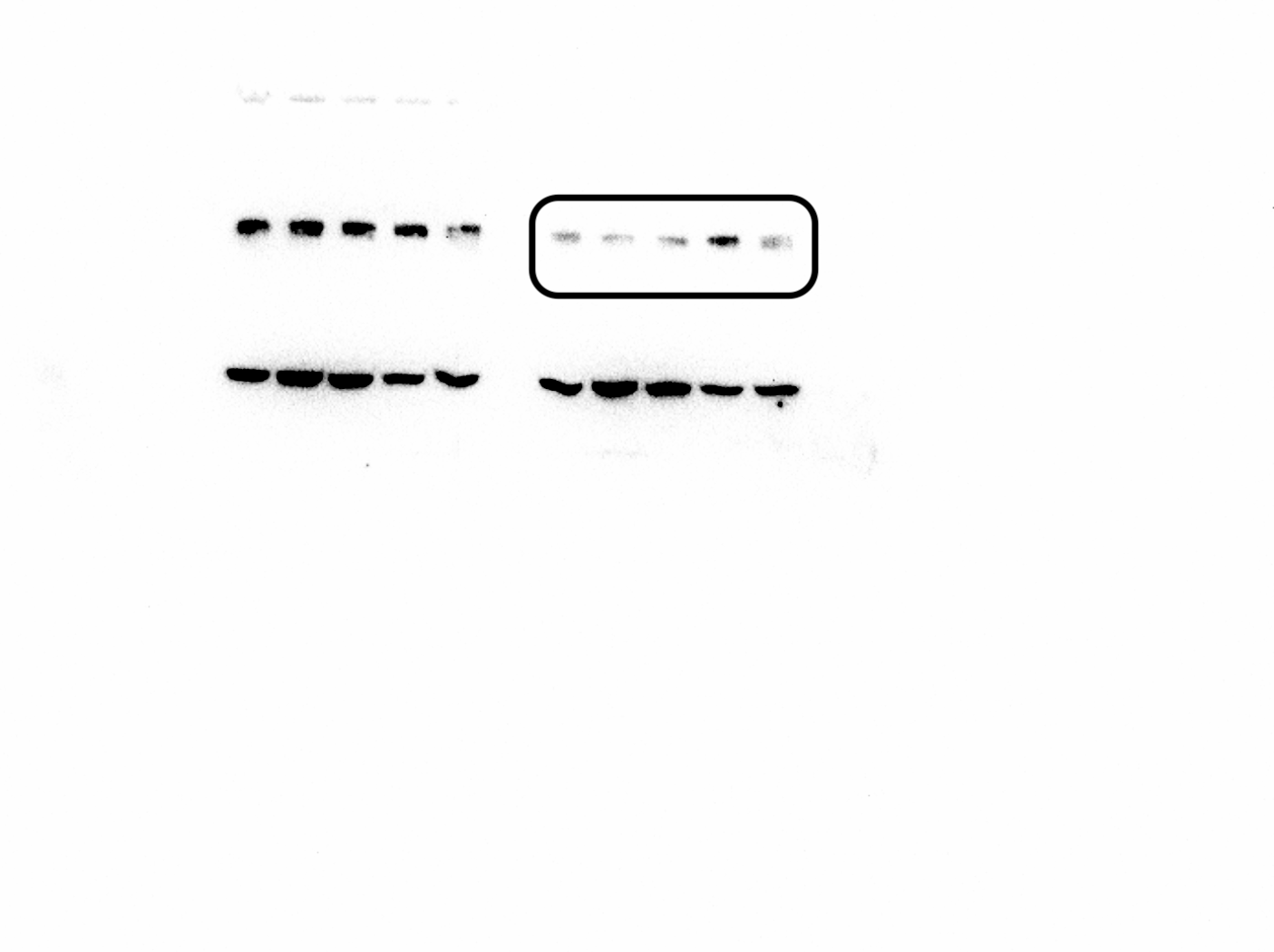

Supplement: Supplementary file 6 — Source data Fig. 5 [file 44318_2024_143_MOESM6_ESM.zip › Figure 5/5C/P-eIF2alpha.tif]

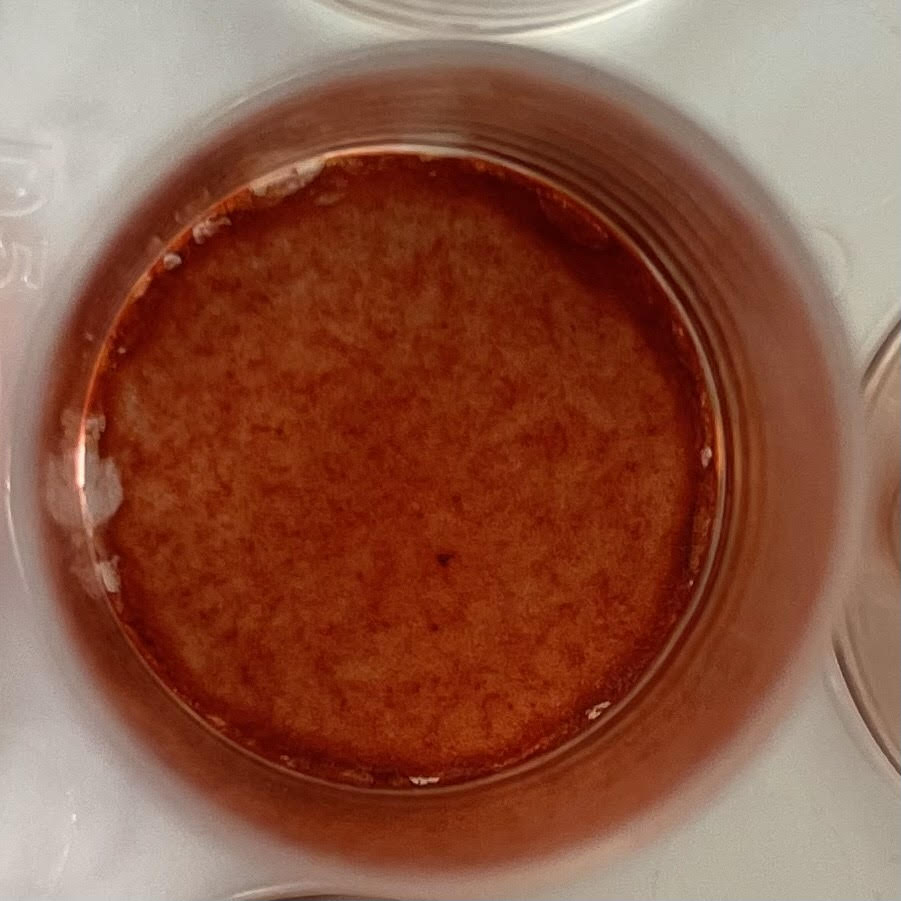

Supplement: Supplementary file 6 — Source data Fig. 5 [file 44318_2024_143_MOESM6_ESM.zip › Figure 5/5A/5A images/L1.jpg]

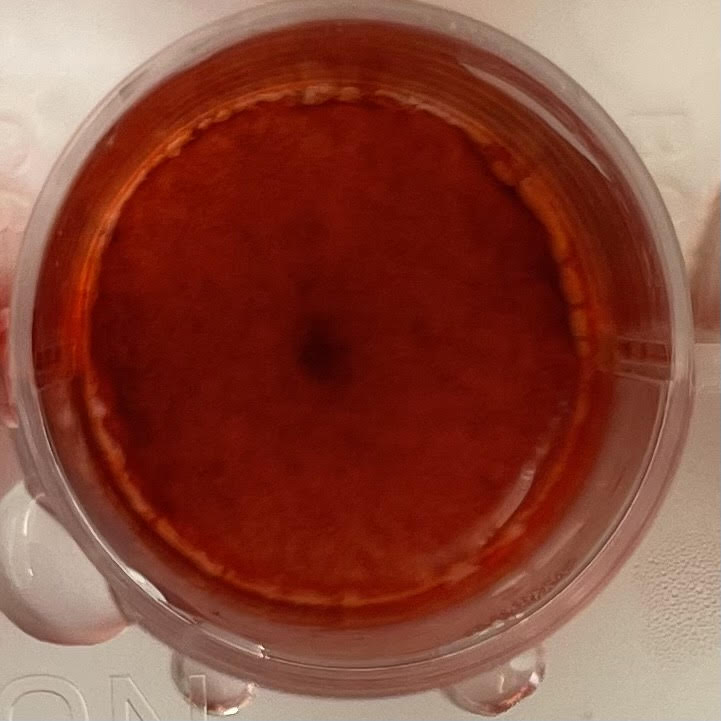

Supplement: Supplementary file 6 — Source data Fig. 5 [file 44318_2024_143_MOESM6_ESM.zip › Figure 5/5A/5A images/L1 3TC.jpg]

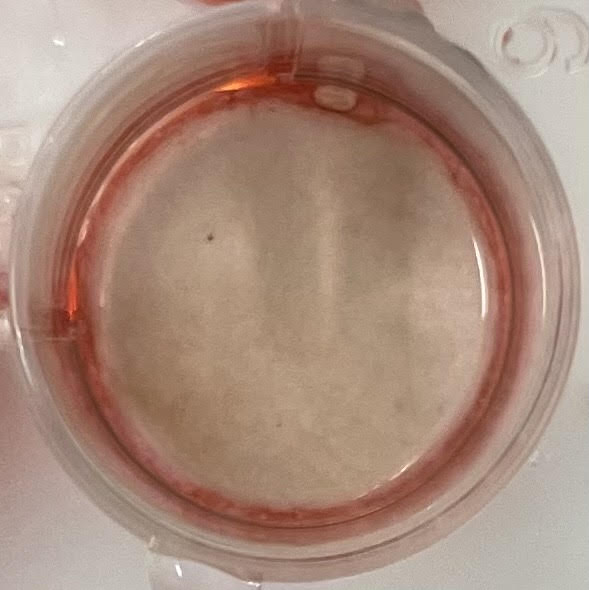

Supplement: Supplementary file 6 — Source data Fig. 5 [file 44318_2024_143_MOESM6_ESM.zip › Figure 5/5A/5A images/3TC.jpg]

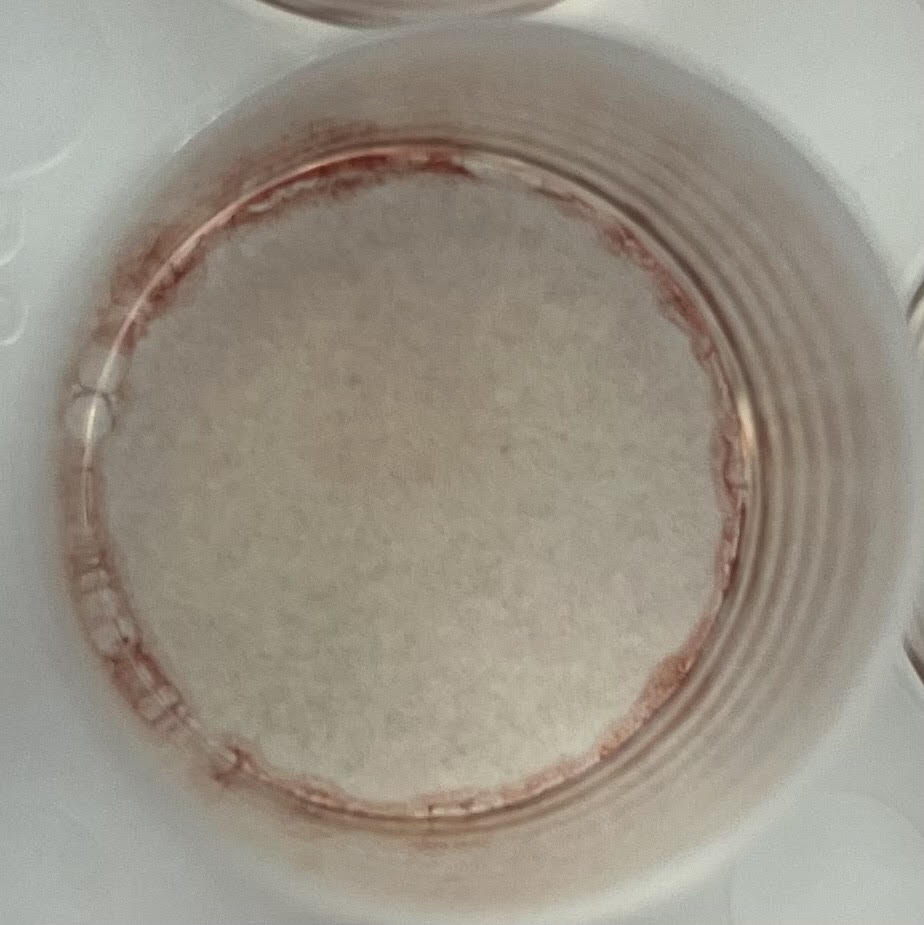

Supplement: Supplementary file 6 — Source data Fig. 5 [file 44318_2024_143_MOESM6_ESM.zip › Figure 5/5A/5A images/G140.jpg]

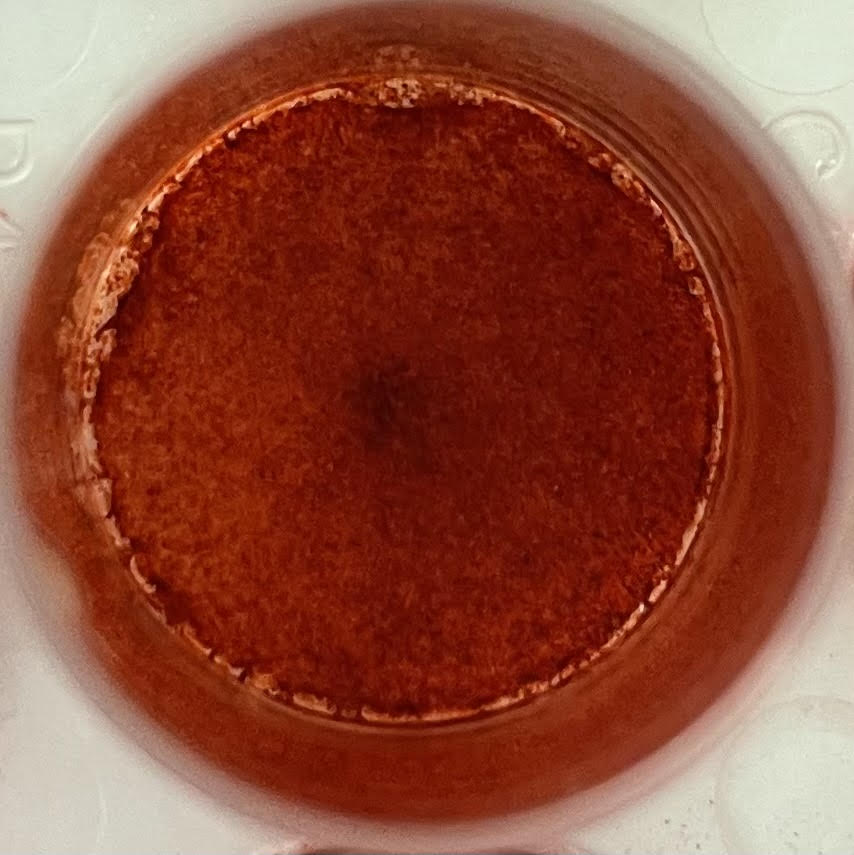

Supplement: Supplementary file 6 — Source data Fig. 5 [file 44318_2024_143_MOESM6_ESM.zip › Figure 5/5A/5A images/L1 G140.jpg]

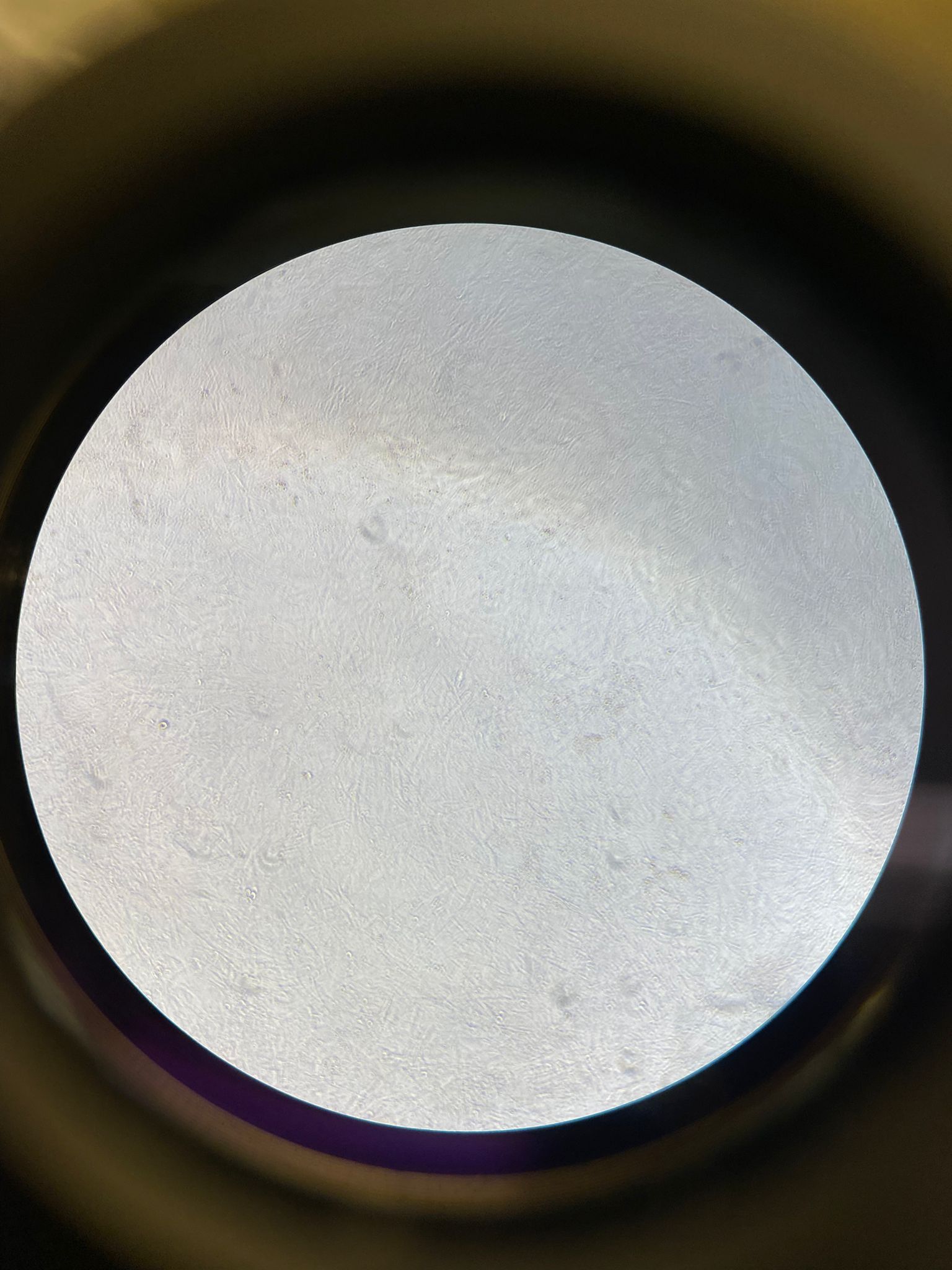

Supplement: Supplementary file 7 — Source data Fig. 6 [file 44318_2024_143_MOESM7_ESM.zip › Figure 6/RFP.JPG]

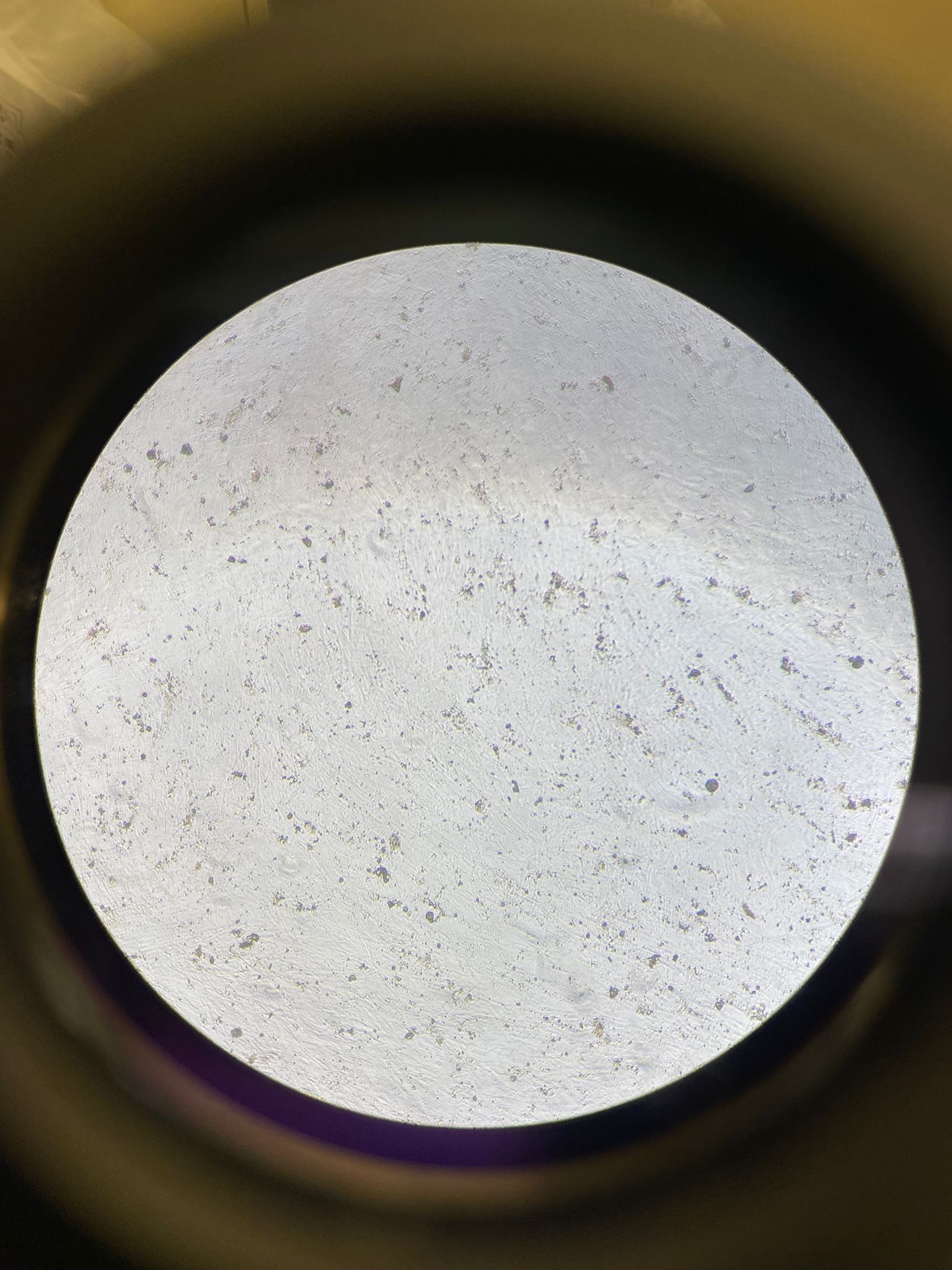

Supplement: Supplementary file 7 — Source data Fig. 6 [file 44318_2024_143_MOESM7_ESM.zip › Figure 6/L1.JPG]

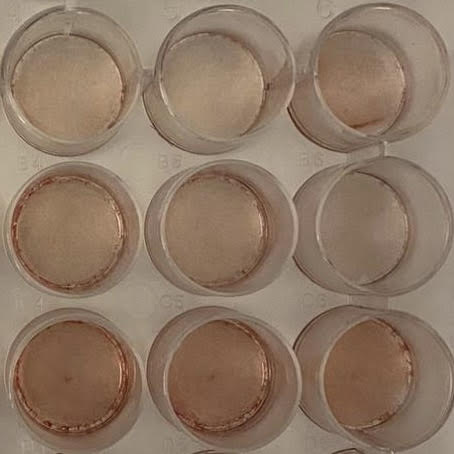

Supplement: Supplementary file 7 — Source data Fig. 6 [file 44318_2024_143_MOESM7_ESM.zip › Figure 6/6A image.jpg]

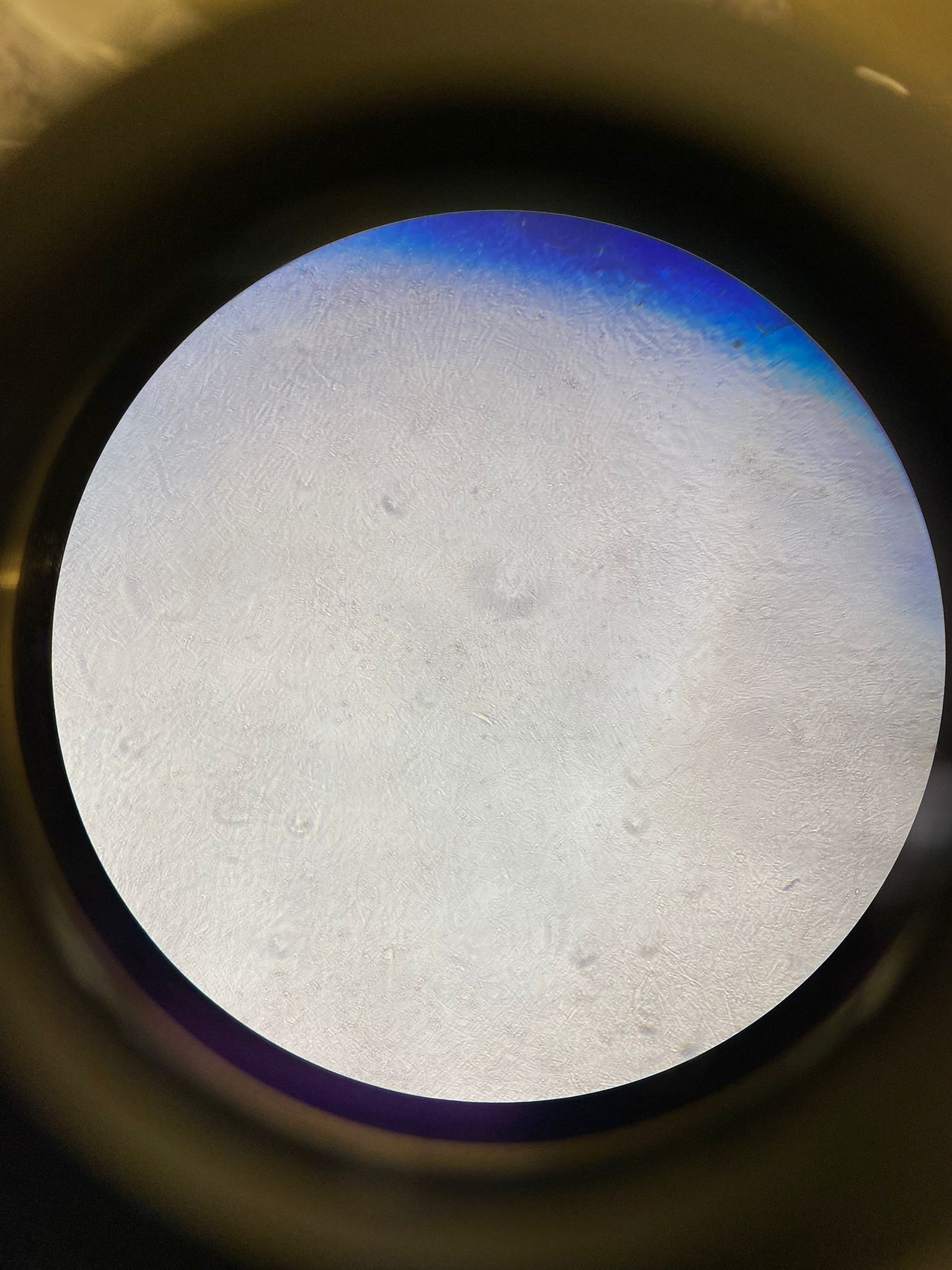

Supplement: Supplementary file 7 — Source data Fig. 6 [file 44318_2024_143_MOESM7_ESM.zip › Figure 6/NT.JPG]
