# Supplementary material for: LINE-1 RNA triggers matrix formation in bone cells via a PKR-mediated inflammatory response
Source: EMBO J. 2024 Jul 1;43(17):3587–603. doi: 10.1038/s44318-024-00143-z (PMC11377738; doi:10.1038/s44318-024-00143-z)

As P-eIF2α and eIF2α have the same molecular weight (36 Kda), we loaded the extract twice in the same gel and we cut the membrane into two pieces:
#1: we hybridized with anti eIF2α Ab (Ab5369)
#2: we hybridized with anti P-eIF2α Ab (Ab32157)
Both the membranes were also hybridized with anti histon H3 Ab

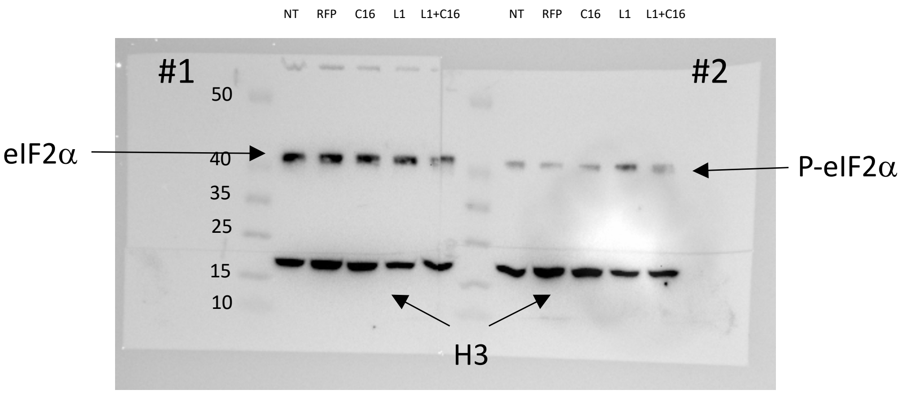

Supplement: Supplementary file 6 — Source data Fig. 5 [file 44318_2024_143_MOESM6_ESM.zip › Figure 5/5C/5C README.pdf]
